# Exquisite Sensitivity of *TP53* Mutant and Basal Breast Cancers to a Dose-Dense Epirubicin−Cyclophosphamide Regimen

**DOI:** 10.1371/journal.pmed.0040090

**Published:** 2007-03-20

**Authors:** Philippe Bertheau, Elisabeth Turpin, David S Rickman, Marc Espié, Aurélien de Reyniès, Jean-Paul Feugeas, Louis-François Plassa, Hany Soliman, Mariana Varna, Anne de Roquancourt, Jacqueline Lehmann-Che, Yves Beuzard, Michel Marty, Jean-Louis Misset, Anne Janin, Hugues de Thé

**Affiliations:** 1 Laboratoire de Pathologie, Assistance Publique/Hôpitaux de Paris, Hôpital Saint Louis, Paris, France; 2 U728, INSERM, Université Paris 7, Paris, France; 3 Laboratoire de Biochimie, Assistance Publique/Hôpitaux de Paris, Hôpital Saint Louis, Paris, France; 4 UMR 7151, CNRS, Université Paris VII, Paris, France; 5 Programme Carte d'Identité des Tumeurs, Ligue Nationale Contre le Cancer, Paris, France; 6 Centre des Maladies du Sein, Assistance Publique/Hôpitaux de Paris, Hôpital Saint Louis, Paris, France; Anderson Cancer Center, United States of America

## Abstract

**Background:**

In breast cancers, only a minority of patients fully benefit from the different chemotherapy regimens currently in use. Identification of markers that could predict the response to a particular regimen would thus be critically important for patient care. In cell lines or animal models, *tumor protein p53 (TP53)* plays a critical role in modulating the response to genotoxic drugs. *TP53* is activated in response to DNA damage and triggers either apoptosis or cell-cycle arrest, which have opposite effects on cell fate. Yet, studies linking *TP53* status and chemotherapy response have so far failed to unambiguously establish this paradigm in patients. Breast cancers with a *TP53* mutation were repeatedly shown to have a poor outcome, but whether this reflects poor response to treatment or greater intrinsic aggressiveness of the tumor is unknown.

**Methods and Findings:**

In this study we analyzed 80 noninflammatory breast cancers treated by frontline (neoadjuvant) chemotherapy. Tumor diagnoses were performed on pretreatment biopsies, and the patients then received six cycles of a dose-dense regimen of 75 mg/m^2^ epirubicin and 1,200 mg/m^2^ cyclophosphamide, given every 14 days. After completion of chemotherapy, all patients underwent mastectomies, thus allowing for a reliable assessment of chemotherapy response. The pretreatment biopsy samples were used to determine the *TP53* status through a highly efficient yeast functional assay and to perform RNA profiling. All 15 complete responses occurred among the 28 *TP53*-mutant tumors. Furthermore, among the *TP53*-mutant tumors, nine out of ten of the highly aggressive basal subtypes (defined by basal cytokeratin [KRT] immunohistochemical staining) experienced complete pathological responses, and only *TP53* status and basal subtype were independent predictors of a complete response. Expression analysis identified many mutant *TP53*-associated genes, including *CDC20, TTK, CDKN2A,* and the stem cell gene *PROM1,* but failed to identify a transcriptional profile associated with complete responses among *TP53* mutant tumors. In patients with unresponsive tumors, mutant *TP53* status predicted significantly shorter overall survival. The 15 patients with responsive *TP53*-mutant tumors, however, had a favorable outcome, suggesting that this chemotherapy regimen can overcome the poor prognosis generally associated with mutant *TP53* status.

**Conclusions:**

This study demonstrates that, in noninflammatory breast cancers, *TP53* status is a key predictive factor for response to this dose-dense epirubicin–cyclophosphamide regimen and further suggests that the basal subtype is exquisitely sensitive to this association. Given the well-established predictive value of complete responses for long-term survival and the poor prognosis of basal and *TP53*-mutant tumors treated with other regimens, this chemotherapy could be particularly suited for breast cancer patients with a mutant *TP53,* particularly those with basal features.

## Abstract 2

Hugues de The and colleagues report that*TP53* status is a predictive factor for responsiveness in breast cancers to a dose-dense epirubicin-cyclophosphamide chemotherapy regimen, and suggests that this regimen might be well suited for patients*TP53* mutant tumors.

## Editors' Summary

**Background.:**

One woman in eight will develop breast cancer during her life. As with other cancers, breast cancer arises when cells accumulate genetic changes (mutations) that allow them to grow uncontrollably and to move around the body. These altered cells are called malignant cells. The normal human breast contains several types of cell, any of which can become malignant. In addition, there is more than one route to malignancy—different sets of genes can be mutated. As a result, breast cancer is a heterogeneous disease that cannot be cured with a single type of treatment. Ideally, oncologists would like to know before they start treating a patient which therapeutic approach is going to be successful for that individual. Recently, researchers have begun to identify molecular changes that might eventually allow oncologists to make such rational treatment decisions. For example, laboratory studies in cell lines or animals indicate that the status of a gene called *TP53* determines the chemotherapy agents (drugs that preferentially kill rapidly dividing cancer cells) to which cells respond. p53, the protein encoded by *TP53*, is a tumor suppressor. That is, in normal cells it prevents unregulated growth by controlling the expression of proteins involved in cell division and cell death. Consequently, p53 is often inactivated during cancer development.

**Why Was This Study Done?:**

Although laboratory studies have linked *TP53* status to chemotherapy responses, little is known about this relationship in human breast cancers. The clinical studies that have investigated whether *TP53* status affects chemotherapy responses have generally found that patients whose tumors contain mutant *TP53* have a poorer response to therapy and/or a shorter survival time than those whose tumors contain normal *TP53*. In this study, the researchers have asked whether *TP53* status affects tumor responses to a dose-intense chemotherapy regimen (frequent, high doses of drugs) given to women with advanced noninflammatory breast cancer before surgery. This type of treatment is called neoadjuvant chemotherapy and is used to shrink tumors before surgery.

**What Did the Researchers Do and Find?:**

The researchers collected breast tumor samples from 80 women before starting six fortnightly cycles of chemotherapy with epirubicin and cyclophosphamide. After this, each woman had her affected breast removed and examined to see whether the chemotherapy had killed the tumor cells. The researchers determined which original tumor samples contained mutated *TP53* and used a technique called microarray expression profiling to document gene expression patterns in them. Overall, 28 tumors contained mutated *TP53*. Strikingly, all 15 tumors that responded completely to neoadjuvant chemotherapy (no tumor cells detectable in the breast tissue after chemotherapy) contained mutated *TP53*. Nine of these responsive tumors were basal-cell–like breast tumors, a particularly aggressive type of breast cancer; only one basal-cell–like, *TP53*-mutated tumor did not respond to chemotherapy. Patients whose tumors were unresponsive to the neoadjuvant chemotherapy but contained mutated *TP53* tended to die sooner than those whose tumors contained normal *TP53* or those with chemotherapy-responsive *TP53*-mutated tumors. Finally, expression profiling identified changes in the expression of many p53-regulated genes, but did not identify an expression profile in the *TP53*-mutated tumors unique to those that responded to chemotherapy.

**What Do These Findings Mean?:**

These findings indicate that noninflammatory breast tumors containing mutant *TP53*—in particular, basal-cell–like tumors—are very sensitive to dose-dense epirubicin and cyclophosphamide chemotherapy. Intensive regimens of this type have rarely been used in previous studies, which might explain the apparent contradiction between these results and the generally poor response to chemotherapy of *TP53*-mutated breast tumors. More tumors now need to be examined to confirm the association between complete response, *TP53* status and basal-cell–like tumors. In addition, although complete tumor responses generally predict good overall survival, longer survival studies than those reported here are needed to show that the tumor response to this particular neoadjuvant chemotherapy regimen translates into improved overall survival. If the present results can be confirmed and extended, dose-dense neoadjuvant chemotherapy with epirubicin and cyclophosphamide could considerably improve the outlook for patients with aggressive *TP53*-mutant, basal-cell–like breast tumors.

**Additional Information.:**

Please access these Web sites via the online version of this summary at http://dx.doi.org/10.1371/journal.pmed.0040090.

- The US National Cancer Institute provides patient and physician information on breast cancer and general information on understanding cancer
- Cancer Research UK offers patient information on cancer and breast cancer
- The MedlinePlus encyclopedia has pages on breast cancer
- Emory University's CancerQuest discusses the biology of cancer, including the role of tumor suppressor proteins
- Wikipedia has pages on p53 (note that Wikipedia is a free online encyclopedia that anyone can edit)

## Introduction

Breast cancers are a heterogeneous group of tumors. While most breast cancer patients receive chemotherapy, less than 20% of those receiving neoadjuvant treatment will reach complete pathological response (disappearance of invasive tumor cells in pathological tissue samples), which strongly predicts long-term survival [1–5]. Predictive molecular determinants for conventionally dosed chemotherapy responses are only emerging [6–8], and very little is known regarding prediction of response to dose-dense treatments.

*Tumor protein p53 (TP53),* the prototypic tumor suppressor gene, is a master gene of stress response that plays a key role in cancer development. TP53 is a transcription factor that controls the expression of many genes implicated in apoptosis *(PUMA* and *BAX)* or cell-cycle regulation *(SFN* and *CDKN1A)*. In animal or cell-line models, *TP53* was shown to play a critical role in the response to DNA damage induced by a number of anticancer therapies [9]. In fact, *TP53* inactivation may promote an exquisite sensitivity to some agents, but resistance to others [10].

The role of *TP53* status in determining the response to a given cytotoxic treatment in patients is largely unsettled, in part because of technical difficulties in establishing *TP53* status in the clinical setting, and because most studies analyzed survival rather than initial tumor response. While tumor disappearance is a direct and unambiguous measure of chemotherapy efficiency, survival is a composite endpoint, which incorporates not only the efficiencies of the different treatments, but also the intrinsic aggressiveness of the disease. In hematological malignancies, several studies have found that mutant *TP53* status is associated with treatment failure [11,12]. Similarly, in breast cancer, patients with *TP53*-mutant tumors often have poor responses to therapy and/or shorter survival than those with normal *TP53* [13–17].

Here, we have used a highly sensitive functional *TP53* assay, which not only determines the functional impact of *TP53* mutations, but also explores other dysfunctions, such as splicing defects [18], to analyze a group of breast cancer patients treated with a neoadjuvant dose-intense chemotherapy regimen.

## Methods

### Patients

Two hundred and six patients with inflammatory and noninflammatory breast cancers treated at a single institution from 1997 to 2003 underwent open incisional biopsy (0.5–3 cm in size) followed by first-line chemotherapy and consented to the study (Tables 1 and S1). One hundred and twenty six patients were not included for the following reasons: inflammatory breast cancer (78 tumors), frozen tumor tissue absent or unsuitable for RNA analysis (29 tumors), absence of further mastectomy (16 cases) or undefined response (three cases: patients P80, P81, P83; see below). The 80 remaining patients were treated with exactly the same regimen (75 mg/m^2^ epirubicin and 1,200 mg/m^2^ cyclophosphamide, delivered every 14 d [19] with granulocyte-colony stimulating factor support in case of febrile neutropenia) and had unambiguous pathological response data. Of the 80 patients, 35 were included in the microarray profiling analysis, as were two additional *TP53*-mutant tumor samples previously excluded from the treatment response analysis as the patients had received another treatment (patient P81) (see Table S1) or had an initial biopsy that may have removed most of the tumor (patient P80). A third additional patient with *TP53* mutant tumor (patient P83) (Table S1) was included for validation studies. Patients were staged IIb, IIIa, IIIb, or IV [20], except four patients (Tables 1 and S1) with a T2N0M0 (stage IIa) disease who had documented tumor doubling time of less than 6 mo (two cases) or who had tumors over 3 cm in size (two cases). The group of 80 patients included in the study was not different from the 48 excluded patients with noninflammatory breast cancer patients in terms of age and TNM Classification of Malignant Tumors stage [20] with, respectively, median age 48 y (24–76 y) and 52 y (29–74 y) and TNM: T1, 0% and 5%; T2, 14% and 19%; T3, 45% and 43%; T4, 41% and 33%; N+, 75% and 71%; and M+, 19% and 13%. Tumors were graded according to the modified Scarff, Bloom, and Richardson system [21]. All patients were screened for metastasis at diagnosis with at least chest x-ray, liver ultrasound, or thoraco-abdomino-pelvic CT scan and bone scintigram. At the time of diagnosis 11 patients had metastases (Table S1). After completion of the planned six cycles of primary chemotherapy—thus approximately 3 mo after diagnosis—the patients underwent mastectomy and axillary lymph node dissection. Pathologically assessed complete response was defined by the complete disappearance of invasive tumor cells in the mastectomy specimen and in the lymph nodes or a single microscopic invasive focus (P4) or residual breast in situ carcinoma (P42, P46, and P69), as previously described [22].

**Table 1:**
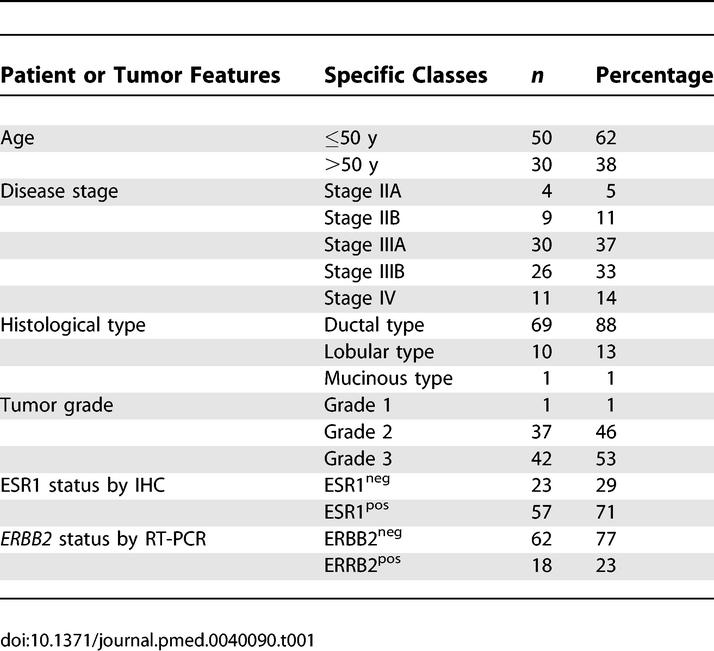
Description of the 80 Patients Analyzed for Chemotherapy Response

### *TP53* Typing

*TP53* status was determined by the yeast functional assay, in which mutant *TP53* transcripts yield red yeast colonies and wild-type transcripts yield white ones [23]. Tumors were considered *TP53* mutant when: (i) more than 15% of the yeast colonies were red, (ii) analysis using the split versions of the test could identify the defect in the 5′ or 3′ part of the gene, confirming the initial determination [24], and (iii) sequence analysis from mutant yeast colonies could identify an unambiguous genetic defect (mutation, deletion, or splicing defects) (Table S2). All tumors with more than 15% red colonies fulfilled these three criteria. Note that the four tumors with low percentage of mutant colonies (15%–25%) all exhibited stop or frame-shift mutations, defects known to be associated with nonsense mediated RNA decay, resulting in low mRNA abundance (Table S2). Prediction of dominant negative activity was performed using IARC software (http://www-p53.iarc.fr/index.html).

### RNA Analysis

RNA was prepared as previously [22], yielding 2–83 μg (mean 17 μg) of RNA. Its quality was verified by Agilent bioanalyzer (http://www.home.agilent.com), and Affymetrix arrays (http://www.affymetrix.com) were performed after a single or a double round of amplification (Table S1). RT-PCR was performed using Gene Expression Assays (Applied Biosystems, http://www.appliedbiosystems.com), except for the *CDKN2A* locus for which we designed specific primers (available upon request).

### Immunohistochemistry

Immunohistochemical analyses were carried out on paraffin sections using antibodies directed against estrogen receptor 1 (ESR1) (clone 6F11) (Novocastra, http://www.vision-bio.com), keratin 5/6 (KRT5/6) (clone D5/16 B4) (DakoCytomation, http://www.dako.com), keratin 17 (KRT17) (clone E3) (DakoCytomation), cyclin-dependent kinase inhibitor 2A (CDKN2A [p16] (clone E6H4) (DakoCytomation), and on frozen sections using antibodies directed against prominin 1 (PROM1 [CD133/1]) (clone AC133) (Miltenyi Biotec, http://www.miltenyibiotec.com). Basal KRT (5/6 or 17) or PROM1 were scored positive if strong cytoplasmic staining was observed in any invasive carcinoma cells [25,26]. For all other antibodies, there was either no staining (negative cases) or a positive staining in more than 10% of tumor cells (positive cases) either in nucleus (ESR1, CDKN2A) and/or in cytoplasm (CDKN2A).

### Statistics and Bioinformatics

Microarray data analysis was based on 37 tumor samples (Table S1): 29 (set S1) processed using the one-cycle target labeling protocol (Affymetrix), and eight (set S2) processed using the two-cycle target-labeling protocol. RT-PCR data analysis was based on 82 tumor samples: 37 from the microarray series (S1 + S2) and 45 new samples (set S3). Except as indicated, all transcriptome analysis was carried out using either an assortment of R-system software (version 1.9.0) packages including those of Bioconductor (version 1.1.1) (http://www.bioconductor.org) or original R code. Raw feature data from Affymetrix HG-U133A GeneChip microarrays were normalized using RMA method (R package *affy* version 1.4.32), which yielded log_2_ intensity expression summary values for each of the 22,283 probe sets. Each set (S1 and S2) was normalized independently. Individual feature data from set S1 and S2 were normalized in batches along with an additional 17 (one-cycle) and 19 (two-cycle) samples unrelated to this study, respectively. Probe sets corresponding to control genes or having an “x” annotation were masked, leaving 19,987 probe sets for further analyses. Clustering analysis of the samples was performed using DNA-Chip Analyzer software (dChip version 1.3, http://www.dchip.org) with (1 − Pearson correlation coefficient) as distance metric and Centroid linkage. The RMA normalized data of 37 samples (S1 + S2) were used in this analysis, based on a subset of 990 probe sets. These 990 probe sets were selected using only set S1 based on the two following criteria: (i) a robust coefficient of variation (rCV) superior to the 95th rCV percentile and below 10, and (ii) a *p*-value of a variance test (see below) less than 0.01. For each probe set, rCV was calculated by first eliminating the highest and lowest expression values for that probe set out of all of the samples. From the remaining samples we divided the standard deviation of the expression values by the mean expression values. This yielded an rCV value for each probe set. The variance calculated for each probe set *P* (variance across all samples) was compared to the median variance from all 19,987 probe sets. The statistic used was ([*n* − 1] × Var(*P*)/Var_med_), where *n* refers to the number of samples. This statistic was compared to a percentile of the Chi^2^ distribution with (*n* − 1) degrees of freedom (this criterion is used in the BRB Array Tools filtering tool, described in the user's manual [http://linus.nci.nih.gov/BRB-ArrayTools.html]) and yielded a *p*-value for each probe set.

We performed all univariate t- and F-tests using BRB Array Tools (version 3.0 b2) on the RMA normalized data for the 19,787 probe sets. We designated a significance level of each univariate test of *p* < 0.001 (except when otherwise indicated). To evaluate the number of false positives (due to multiple testing), we used a Monte Carlo approach (implemented in BRB comparison tool) based on 1,000 random sample label permutations. This method calculates the false discovery rate (with a probability of 90%), on the basis of the number *(n)* of probe sets (*n* first probe sets ordered by their *p*-value from the univariate test) identified where the false discovery is relative to the chosen level of significance for the univariate test (e.g., *p* = 0.001). This method also evaluates the number *n*′ of probe sets (*n*′ first probe sets ordered by their *p*-value from the univariate test) for which the number of false discoveries is less than 10 (with a probability of 90%). See Dataset S1 for the statistics related to the definitions of the genes associated with the C1, C2, and C3 groups.

Based on the t-tests of *TP53* status described above, we sorted the 1,599 obtained probe sets by fold change (ratio of the geometric mean intensity in the 17 *TP53* mutant samples versus the geometric mean intensity in the 12 *TP53* wild-type samples). We then selected the ten well-characterized probe sets having the highest fold change: 204304_s_at *(PROM1),* 205347_s_at *(TMSNB),* 209016_s_at *(KRT7),* 213338_at *(RIS1),* 209803_s_at *(PHLDA2),* 36711_at *(MAFF),* 202912_at *(ADM),* 204822_at *(TTK),* 202870_s_at *(CDC20),* and 207039_at *(CDKN2A)* (219010_at *[FLJ10901]* was filtered, as it is not well characterized). We also selected *ESR1* (second highest inverse fold change). This yielded 12 gene transcripts for further study with RT-PCR (for *CDKN2A* we used two targets: p14 and p16). Due to a lack of sample RNA, only 948 data points (Ct [gene, sample]) from a total of 984 possible data points (12 genes × 82 samples) were available. These Ct values were normalized to yield ddCt values, based on the following calculation: for a given sample *s,* ddCt_gene_ *(s)* = dCt_gene_(*s*) − (Median of dCt_gene_ across all samples), with dCt_gene_ *(s)* = Ct_gene_ *(s)* − Ct_calibrator_ *(s),* using the gene *TBP* (TATA-binding protein) as the reference gene. See Dataset S1 for the statistics associated with the RT-PCR data of these 12 genes.

### Statistical Methods for the Analysis of the Clinical Data

The association between treatment response and potential prognostic variables (age, tumor grade, cytoplasmic staining of CDKN2A, presence of KRT5/6 or 17, *ERBB2* mRNA expression, ESR1 protein expression, T stage from TNM classification, and *TP53* mutation status) was tested using Fisher's exact test. Because the age of the patients represents a continuous variable, patients were divided into two classes using a cutoff at age 50. Stratified association analyses were performed using the Mantel-Haenszel test. Kaplan-Meier survival curves and log-rank *p*-values were calculated on the basis of either event-free survival (considering local relapse or distant metastasis as an event) in the population of patients that were free of metastasis at diagnosis or overall survival on the complete population.

## Results

### All Complete Responses to a Dose-Dense Chemotherapy Harbor *TP53* Mutations

**Figure 1:**
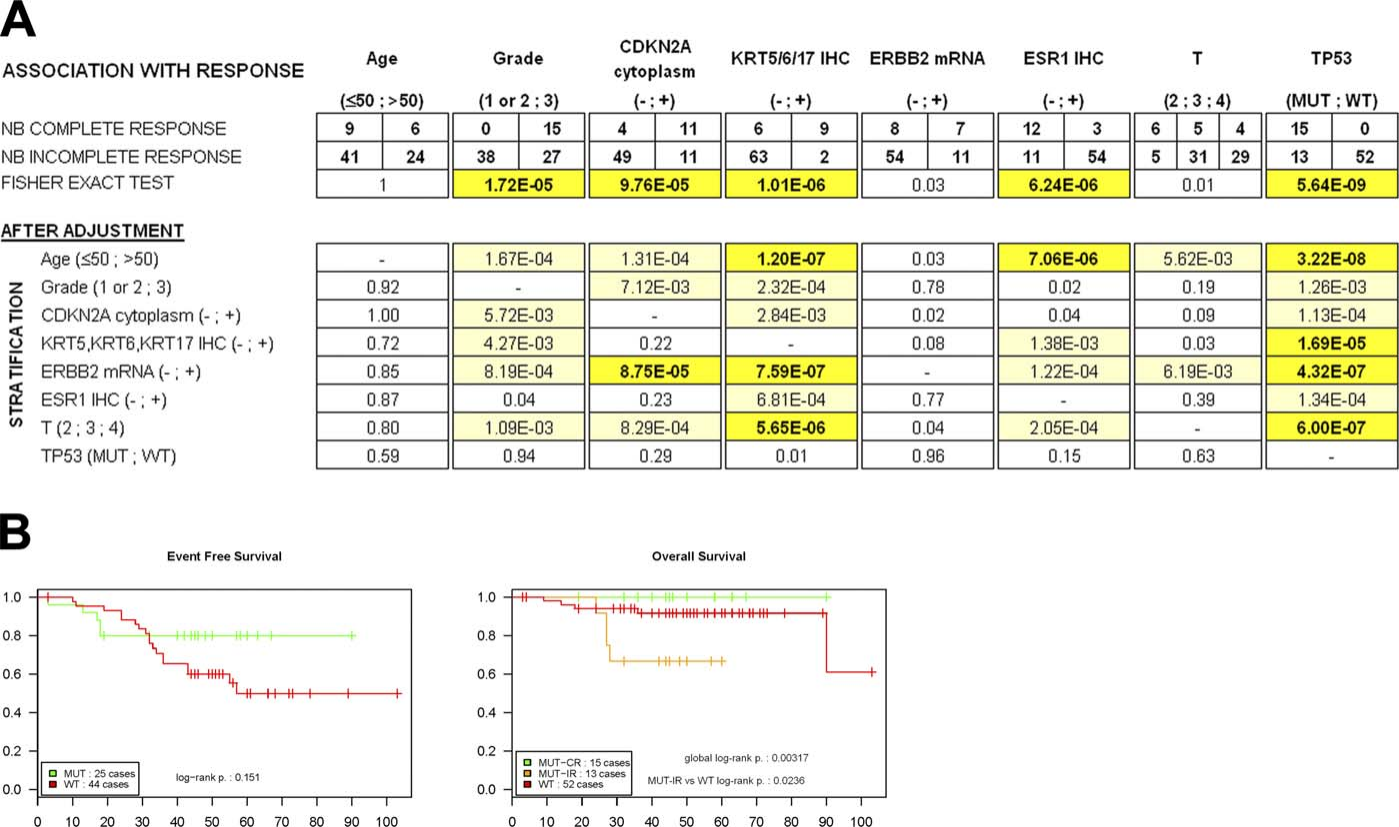
Response to Chemotherapy (A) Top: distribution of the pathological response to chemotherapy of the 80 patients according to *TP53* status as well as other clinical or biological annotations. T, T stage from the TNM classification. *p*-Values are below each contingency table. Bottom: results from adjusted Chi^2^ tests. The variables tested for the prediction of complete response are shown in the columns; the stratification variable is listed in the first column. The *p*-values are color coded (dark yellow and bold type, *p* < 0.0001; light yellow, 0.0001 < *p* < 0.01). (B) Left graph: Kaplan-Meier analysis of the event-free survival of the patients without metastatic extension at diagnosis, all treated with the same regimen (including patient P80, see Table S1). Note that all five relapses in the *TP53* mutant group occurred in the first 18 months, while in the *TP53* wild-type group, relapses were still observed when survival of many patients was censored. Right graph: overall survival stratified by *TP53* status and complete response.

We had previously observed, in 49 of our 206 patients, that complete responses were preferentially achieved when tumors bore *TP53* mutations [22]. Analysis of the consecutive cohort of 31 patients independently validated this association (*p* = 0.0003), which became compelling (*p* = 10^−8^) when the initial subset and the consecutive cohort were pooled (Figure 1A). Indeed, not a single tumor with a normal *TP53* genotype (52 cases) had a complete response, while 15 out of 28 *TP53*-mutant tumors exhibited complete pathological responses. The predicted effects of the observed mutations and the distribution of R72P polymorphism were not significantly different in tumors with complete or incomplete responses (Table S2).

### *TP53* Status and Basal Features Are Independent Predictors of Complete Response

Many studies have demonstrated that mutant *TP53* status is tightly associated with ESR1^neg^ and grade 3 breast cancers. In addition, ESR1^pos^ status was previously associated with chemotherapy resistance [7]. To elucidate the respective contribution of these factors in predicting chemotherapy response, we analyzed the distribution of age, T stage (from TNM classification), tumor grade, *TP53* status, staining for ESR1, KRT5/6 or 17, and *ERBB2* RNA expression among the 80 patients (Figure 1A; Tables 1 and S1). We also included staining for CDKN2A, a major tumor suppressor whose expression is tightly associated with *TP53* status (see below). High transcript and protein levels of CDKN2A are associated with cytoplasmic staining (Figure S1). Therefore we used the cytoplasmic staining pattern as the criteria for deeming a sample CDKN2A positive or negative, as in previous studies [27–29]. No or weakly significant differences in age, T stage, or *ERBB2* status were found between complete and incomplete responses. In contrast, grade, *TP53* status, ESR1, KRT5/6 or 17, and cytoplasmic CDKN2A (p16) staining were all strongly associated with response (Figure 1A). As there are no complete responses in the *TP53* wt group, the estimation of a regression coefficient using the multivariate logistic regression model is impossible, this coefficient being theoretically infinite in such a situation, precluding a multivariate analysis. Yet, *TP53* status remained significantly linked to a response in univariate analyses, irrespective of the stratification (age, T stage or tumor grade, KRT5/6 or 17 presence, CDKN2A cytoplasmic staining [p16], *ERBB2* mRNA, or ESR1 protein expression) in Mantel-Haenszel tests (Figure 1A). In contrast, with the exception of basal features (KRT5/6 or 17 positivity), all the other individually significant variables significantly associated with complete response prior to stratification for *TP53* (Figure 1A) no longer remained significant after stratification for *TP53* status (Figure 1A). Moreover, among the patients with *TP53* mutations, the 15 tumors with a complete response had sizes and grades (six T2s, five T3s, and four T4s, all grade 3) comparable to the 13 who did not reach a complete response (three T2s, five T3s, and five T4s, all but one grade 3). Altogether, despite the impossibility of a formal multivariate regression analysis, it appears that *TP53* and, to a lesser extent, basal features are the only important predictors of complete pathological response.

### *TP53* Status and Survival

When patients with metastatic disease at diagnosis were excluded from the analysis, most patients with a *TP53* mutant tumor had favorable event-free survival, although with the current median follow-up (46 mo, range 3–103 mo), it did not reach statistical significance (Figure 1B, left graph; Table S1). Patients with a basal phenotype or complete responses also had favorable outcomes (Table S1), in line with the well-known association between a complete response and prolonged survival [1–5]. This observation is particularly remarkable, because *TP53* mutant tumors have been reported in several studies to have a shorter survival than *TP53* wild-type ones [13,14,30,31]. Patients with wild-type *TP53* tumors had a relatively favorable overall survival, despite the lack of complete response (Figure 1B, right graph, red curve). Interestingly, patients with *TP53*-mutant tumors who did not reach complete response had a significantly shorter overall survival than other tumors (*p* = 0.02) (Figure 1B, right graph, orange curve). Again, absence of complete responses in the wild-type *TP53* group precluded a multivariate survival analysis.

### Can Transcriptional Profiling Predict Complete Response?

**Figure 2:**
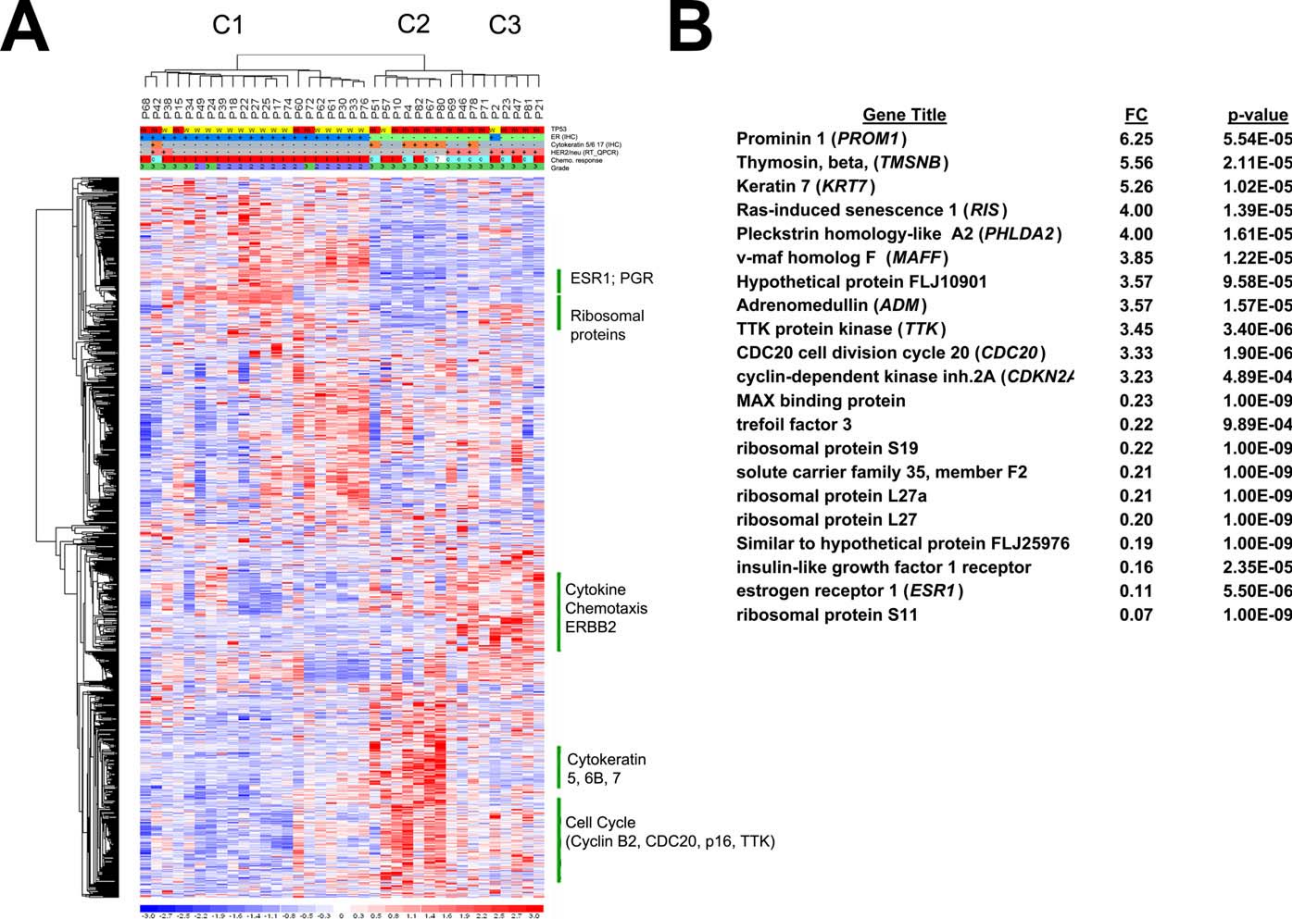
Microarray Data (A) Hierarchical clustering based on the 990 most varying genes in 37 tumors with Affymetrix-grade RNA. C1, C2, and C3 denote the three tumor clusters. Annotations: *TP53* status (red, mutant; yellow, wild-type); ESR1 (immunohistochemistry) (blue, positive; green, negative); basal cytokeratins (KRT5/6 or 17, immunohistochemistry) (orange, positive; gray, negative); *ERBB2* (RT-PCR) (pink, positive; gray, negative); complete pathological response to chemotherapy (blue, complete; red, incomplete); and tumor grade (green, grade 3; purple, grade 1 or 2). For chemotherapy response, patients treated with other regimens are indicated by a question mark. P1, P2, etc. refer to the patient's references in Table S1. (B) Genes linked to *TP53* status (t-tests): the top and bottom genes (classified by fold changes [FC]) are shown.

In an attempt to better characterize the responsive tumors, we performed expression analyses using microarrays. Of the 83 (three additional *TP53* mutant tumor samples were considered for the profiling study, see Methods) breast tumors considered for gene-profiling analysis, 37 had enough high-quality RNA extracted from biopsies before chemotherapy to be analyzed with the arrays, leaving the remaining 46 samples for validation studies. Consistent with previous reports [32], hierarchical cluster analyses of these 37 cancers identified three robust clusters of patients (C1, C2, and C3), as well as the genes defining these patients' clusters (Figure 2A; Tables S3–S5).

To define genes specifically associated with complete responses, we then performed t-tests based on the Affymetrix data on the 28 tumors (nine complete responses and 19 incomplete responses) processed using a single amplification step (see Methods). We identified 77 probe sets using a *p*-value threshold of 0.001, with a high false discovery rate of 26% (Table S6). Using the same sample population, we identified 1,599 *TP53* mutant–associated genes, with a false discovery rate less than 0.1%. As expected, the great majority (54 of 77) of genes associated with complete response were also *TP53-*associated genes, the others most likely being false positive (Table S7). Importantly, within the subgroup of *TP53* mutant tumors, t-tests failed to identify more genes associated with complete responses than would be expected by chance.

**Figure 3:**
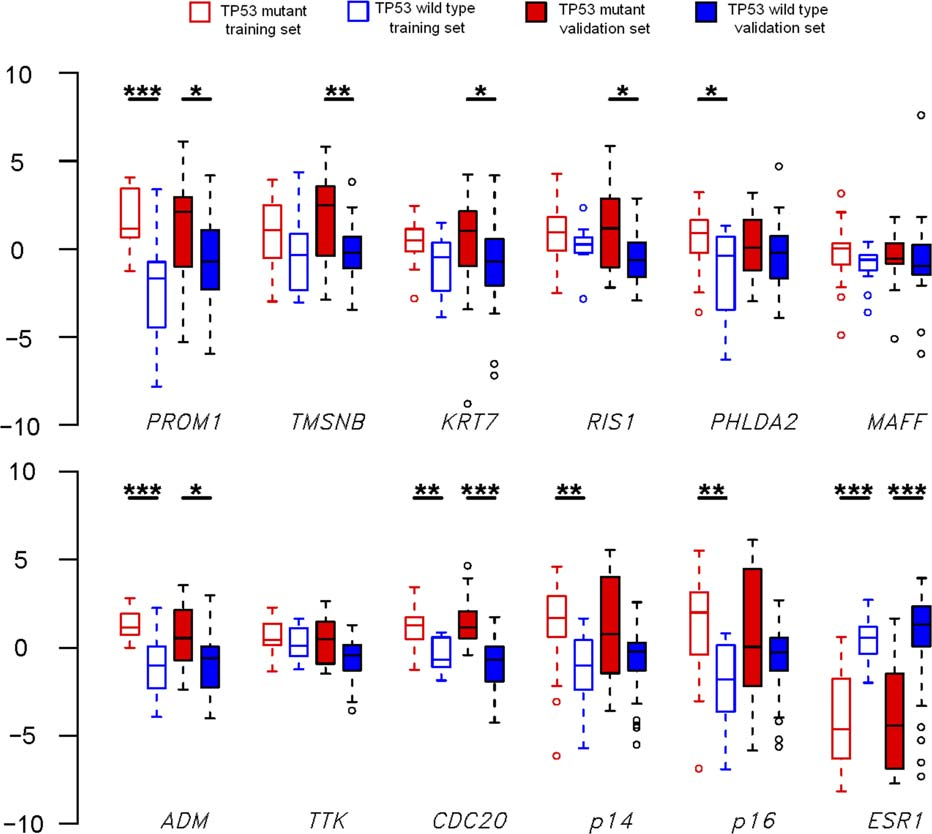
Validation of Mutant *TP53* Profile in Breast Cancers For each of the 12 most varying genes (11 overexpressed and one underexpressed), four box plots are shown, representing the distribution of the ddCt values for the four following groups of samples: (i) *TP53* mutant from the training set (empty red box), (ii) *TP53* wild type from the training set (empty blue box), (iii) *TP53* mutant from the validation set (filled red box), and (iv) *TP53* wild type from the validation set (filled blue box). *p < 0.05; **p < 0.01, ***p < 0.001 (t-tests). Small circles indicate outliers.

Examining the genes associated with *TP53* status, we found that, as expected, wild-type *TP53* tumors overexpressed many *ESR1*-associated genes [30,32,33], as well as ribosomal protein genes (Figure 2B). In contrast, *TP53* mutant tumors overexpressed, among others, a number of master genes (such as *PROM1, cell division cycle 20 homolog [CDC20], CDKN2A, TTK protein kinase [TTK],* and *adrenomedullin [ADM]*) (Figure 2B; Table S7). Most of these mutant *TP53* associated genes did not overlap with the ones described in a recent study that used direct *TP53* sequencing and a different chip [14]. To exclude the possibility that the genes associated with *TP53* mutant status might merely reflect ESR1^neg^ status, we repeated this analysis within the subgroup of ESR1^pos^ tumors. We again found six out of the top ten genes previously identified among 15 top genes differentiating *TP53*-mutant versus wild-type tumors (Table S8) within the *ESR1^pos^* subgroup, demonstrating that the *TP53*-mutant genes that we have identified do not simply reflect ESR1^neg^ status. In order to validate these mutant *TP53*-associated genes, we then assessed by quantitative RT-PCR the levels of expression of the first ten genes with the largest fold difference (Figure 2B; Table S7), as well as *ESR1*. Most of the 12 gene transcripts tested showed significant differences between *TP53*-mutant or wild-type tumors, not only in the tumor set used to select those genes, but also in the rest of the samples, the ESR1^pos^ subgroup, and the complete population (Dataset S1; Figure 3).

To characterize the highly chemosensitive tumors that express the KRT5/6 or KRT17 proteins by immunohistochemistry, we performed t-tests based on the Affymetrix data of *TP53* mutant tumors and found overexpression of basal cytokeratins and many genes previously identified in myoepithelial cells, consistent with the proposed origin of these tumors (Table S9) [26].

Lastly, we compared transcript levels to the immunohistochemical detection of ESR1, CDKN2A (p16), PROM1, KRT5, and KRT17 in all samples, which demonstrated very significant differences in mRNA levels between immunohistochemistry (IHC)+ and IHC− tumors (*p* < 0.001) (Figure S1). In particular, in most of the *TP53*-mutant and nine out of ten of the basal tumors (defined as KRT5- or KRT17-staining), PROM1 staining was strongly positive in a few cancer cells that could be breast cancer stem cells.

## Discussion

We show that in noninflammatory breast cancers, *TP53* mutations are highly predictive of complete responses to a dose-intense neoadjuvant epirubicin–cyclophosphamide chemotherapy regimen. Why did previous studies fail to identify this relationship? We believe that the results reported here reflect the use of a very aggressive DNA-damaging regimen and of a highly efficient technique to determine *TP53* status, which detects not only mutations and deletions, but also abnormal splicing events [18]. It is also possible that some previous studies were biased by the presence of inflammatory breast cancers in the study population.

As *TP53* is a master gene controlling acute DNA-damage response, its functional integrity was expected to control cell survival, particularly after a dose-dense regimen triggering both double-strand breaks and DNA cross-links. *TP53* activation induces a plethora of biological responses (transient cell cycle arrest, senescence, and/or apoptosis) that may have opposing effects on cancer therapy responses [34]. *TP53* or *CDKN1A* deficiencies, which impede chemotherapy-induced cell-cycle arrest, dramatically increase anthracyclin-induced cell death ex vivo [10]. TP53-induced cell-cycle arrest should also protect cells from cyclophosphamide, which, in vivo, only kills rapidly cycling cells [35]. Note that one of the few genes induced by chemotherapy in breast cancers is *p21 CDKN1A* [36]. In contrast, *TP53*-mutant cells that cannot arrest in the cell cycle would subsequently progress towards mitotic catastrophe [10,37]. *Topoisomerase II A (TOP2A)* expression is an important determinant of epirubicin response [38]. When we investigated the expression of *TOP2A* by RT-PCR in all the samples, no significant differences in transcript levels were observed between responsive and unresponsive tumors, independently of their *TP53* status (unpublished data). We suggest that the very high dose of cyclophosphamide used here bypasses the influence of *Top2A* in controlling response to epirubicin. Whatever the exact mechanism(s) involved, the exquisite chemosensitivity of *TP53* mutant breast cancers strongly favors the hypothesis that here, *TP53* activation principally blocks cell cycle progression, rather than triggering a cell death program [34]. In line with our observations, transient pharmacological inhibition of *TP53* was recently proposed to boost chemotherapy efficacy [39].

The expression studies performed here have identified and validated a transcriptional profile associated with mutant *TP53* status (and hence indirectly to response), but failed to identify, within the *TP53* mutant group, a specific profile associated with complete response. Yet, many of these *TP53* mutant–associated genes would be helpful tools in characterizing the pathophysiology of these tumors. *PROM1,* a marker of normal or cancer stem cells [40], is the gene with the highest fold-difference in *TP53-*mutant breast tumors (Figure 2B) or several other tumor types (unpublished data). Basal cancers, which were proposed to have a stem cell origin, are virtually all *TP53* mutant and express high levels of *PROM1* transcript and protein (Figures 3 and S1). Very high expression of *CDKN2A* transcript was associated with cytoplasmic p16 staining (Figure S1) and was almost exclusively observed in *TP53* mutant tumors, consistent with the absence of selection pressure for silencing of the *CDKN2A* locus when *TP53* is mutated. Note that several studies have shown that cytoplasmic p16 was associated with poor survival [27–29], while here it predicts responsiveness (Figure 1A). Finally, a large number of cell-cycle checkpoint genes *(CDKN2A, CKS2, TTK, CDC20, CENPA, CCNB1, CCNB2,* and *BUB1)* were overexpressed in *TP53*-mutant tumors, which may facilitate the execution of mitotic catastrophe upon chemotherapy exposure [41].

Several studies have repeatedly demonstrated worse outcomes for patients with *TP53*-mutant breast tumors when treated with a standard regimen, implying that mutant *TP53* is a poor prognostic factor [13,14,16,30]. In our study, mutant *TP53* was the major independent predictive factor of complete pathological responses, which itself is the major predictor of long-term survival in patients previously treated with the same protocol [3]. Accordingly, *TP53*-mutant patients from this study appeared to have a more favorable event-free survival than those with a wild-type *TP53* (Figure 1B). However, if patients with *TP53*-mutant tumors failed to reach a complete response, their survival was significantly worse than those with *TP53* wild-type, unresponsive tumors (*p* = 0.02, Figure 1B), consistent with previous studies. The coexistence, among *TP53*-mutant tumors, of two very different types of tumors (i.e., the responsive ones with good prognosis and the unresponsive ones with very poor prognosis) may account for the conflicting results of the literature regarding the prognostic value of *TP53* status in breast cancer. The apparent paradox between the results of our study and previous ones is likely treatment-related, as the regimen used here had a dose intensity two to four times higher than most others, with only two-week intervals between courses. However, some recent studies are in line with our data: ESR1^neg^ breast cancers (which are mostly *TP53* mutant) were found to be more sensitive to neoadjuvant chemotherapy [7] and, in a pilot study, *TP53* staining was associated with favorable responses to preoperative anthracyclins [42]. Despite the small number of cases analyzed, our data also suggest that basal cancers, described as having a dismal prognosis in all other studies [25,26,33,43], are exquisitely sensitive to this association, possibly because of their rapid proliferation, which favors cyclophosphamide-induced cell death [35,43]. Thus, *TP53*-mutant tumors may have a poor outcome when treated with conventionally dosed chemotherapies, but may be highly responsive to dose intensification. Hence, in the neoadjuvant, or even in the adjuvant settings [44], this type of dose-intense regimen could be particularly suited for patients with basal and *TP53-*mutant tumors.

## Supporting Information

Alternative Language Abstract S1 Translation of the Abstract into Arabic by A. Bazarbachi

Alternative Language Abstract S2 Translation of the Abstract into Chinese by B. Chen and J. Zhu

Alternative Language Abstract S3 Translation of the Abstract into French by P. Bertheau and H. de Thé

Alternative Language Abstract S4 Translation of the Abstract into German by N. Schwartz and J. Lehmann-Che

Alternative Language Abstract S5 Translation of the Abstract into Japanese by H. Murata and Y. Takahashi

Alternative Language Abstract S6 Translation of the Abstract into Portuguese by J. Pedroso de Oliveira

Alternative Language Abstract S7 Translation of the Abstract into Romanian by M. Varna

Alternative Language Abstract S8 Translation of the Abstract into Spanish by H. Pisonero and P. Santa Olalla

Dataset S1 Supplementary Statistical Data (290 KB PDF)

Figure S1 Protein Validation Studies Left: example of tumors scored positive or negative by immunohistochemistry. Right: mean RNA levels for tumors positive and negative for ESR1, KRT5, KRT17, PROM1, and CDKN2A (p16). Pos, positive; neg, negative; cyt, cytoplasmic; nuc, nuclear; ***, *p* < 0.001 when comparing the levels of mRNA between IHC+ and IHC− tumors. Bars indicate 50 μm.
(1.1 MB PDF)

Table S1 Characteristics of Patients and Tumors Sets: S1, single amplification for microarray; S2, double amplification for microarray; S3, tumors not analyzed by microarray, but used for RT-PCR and/or immunohistochemical studies. The raw data for quantitative RT-PCR analysis (ddCT) or other immunohistochemical tests are highlighted in yellow. C, complete; DOD, died of disease; EC, epirubicin/cyclophosphamide; ET, epirubicin/docetaxel; KRT, cytokeratin; NA, not applicable; NC, not complete; nd, not done; wd, without disease; META, metastatic; METADiag, metastatic at diagnosis; TNM, tumor, node, metastasis.
(52 KB PDF)

Table S2 Summary of the Data on the 28 *TP53* Mutations in Patients Studied for Pathological Response to Chemotherapy FASAY, functional analysis of separated allele in yeast [23,24] codon 72; R, arginine; P, proline.
(28 KB PDF)

Table S3 Genes Specific for Patient Cluster 1 Probe set, ratios of mean values, *p*-values, gene symbol, and gene name are indicated. Class specific gene lists were generated by the intersection of all group-wise *t*-tests with an F-test of all three classes (i.e., C1-specific genes, intersection of C1 versus C2, C1 versus C3 and F test, etc.).
(68 KB PDF)

Table S4 Genes Specific for Patient Cluster 2 For details, see legend of Table S3.
(86 KB PDF)

Table S5 Genes Specific for Patient Cluster 3 For details, see legend of Table S3.
(59 KB PDF)

Table S6 List of the 77 Probe Sets That Are Differentially Expressed between Tumors That Exhibited a Complete Response to Chemotherapy and Those That Did Not Probe sets associated with *TP53* status are indicated. The false discovery rate in this experiment is 26%.
(26 KB PDF)

Table S7 List of the Probe Sets with the Highest Differential Expression in *TP53*-Mutant Versus *TP53* Wild-Type Tumors Fold ratio and *p*-values are indicated. A total of 1,599 significant genes were identified.
(46 KB PDF)

Table S8 List of Probe Sets Associated with *TP53* Mutant Status in the Subgroup of ESR1^pos^ Tumors Note that several of the top genes (when ordered by decreasing fold-change values) were previously identified in the complete population (Table S7). Genes in common with Table S7 are highlighted.
(31 KB PDF)

Table S9 Genes Identified by t-Tests of *TP53*-Mutant KRT5/6^pos^ or KRT17^pos^ Tumors Versus KRT5/6 ^neg^ and KRT17^neg^ Tumors (42 KB PDF)

### Accession Numbers

The primary data for the microarrays were deposited at the European Bioinformatics Institute (EBI) Web site (http://www.ebi.ac.uk) (ArrayExpress number E-TABM-43). Accession numbers for the genes and corresponding proteins cited in the main text are given below in PubMed format (gene, NM_; protein, NP_): *PROM1* (NM_006017, NP_006008); *TMSNB* (NM_021992, NP068832); *KRT7* (NM_005556, NP_005547); *RIS1* (NM_057212, NP_476560); *PHLDA2* (NM_003311, NP_003302); *MAFF* (NM_010755, NP_034885); *ADM* (NM_001124, NP_001115); *TTK* (NM_003318, NP_003309); *CDC20* (NM_001255, NP_001246); *CDKN2A* (NM_000077, NP_000068); *CDKN2A* (NM_058195, NP_478102); *ESR1* (NM_000125, NP_000116); *ERBB2* (NM_001005862, NP_001005862); *KRT5* (NM_000424, NP_000415); *KRT6* (NM_080747, NP_542785); *KRT17* (NM_000422, NP_000413); *TOP2A* (NM_001067, NP_001058.2); *PUMA* (NM_014417 , NP_055232); *BAX* (NM_138763, NP_620118); *TP53* (NM_000546,NP_006133); *BUB1* (NM_004336, NP_000380); *CCNB2* (NM_004701, NP_004692); *CDKN1A* (NM_000389, NP_000380); *CCNB1* (NM_031966, NP_114172); *CENPA* (NM_001809, NP_001800); and *CKS2* (NM_001827, NP_001818.1).
