# Supplementary material for: Exquisite Sensitivity of TP53 Mutant and Basal Breast Cancers to a Dose-Dense Epirubicin−Cyclophosphamide Regimen
Source: PLoS Med. 2007 Mar 20;4(3):e90. doi: 10.1371/journal.pmed.0040090 (PMC1831731; doi:10.1371/journal.pmed.0040090)
Supplement: Alternative Language Abstract S1 [file pmed.0040090.sd001.pdf]

تمهيد أو خلفية البحث

هناك أقلية من مريضات سرطان الثدي اللواتي يستفدن، الى حد أقصى، من العلاجات الكيميائية المتوفرة حاليا. لذلك، فإن السعي لتحديد المؤشرات التي تتنبيء بمدى فعالية نظام من أنظمة العلاج يرتدي أهمية كبرى في مجال نجاح العلاج. ولقد أظهرت التجارب على الخلايا المزروعة أو على النماذج المخبرية الحيوانية أن العامل TP53 يلعب دورا حاسما في مجال تحديد الاستجابات الى العلاج بواسطة المضادات الجينية. بحيث يؤدي تنشيطه ردا على مدمرات الحمض النووي (DNA) الى واحد من ردي فعل متناقضين: إما الى إطلاق عملية الذبول الخلوي (Apoptosis) أو وقف دورة الأنقسام الخلوي. ولحد الآن، لم تتمكن الدراسات المركزة من الوصول الى إمكانية تحديد رابط نهائي بين العامل TP53 ومردود العلاج الكيميائي للسرطان. لهذا كان من المعروف أن سرطانات الثدي المصحوبة بطفرة (mutation) في العامل TP53 هي بشكل عام عصية على العلاج ولكن لا يمكن الجزم بكون ذلك يعود الى قصور في فعالية العلاج الكيميائي أو الى عوامل داخلية تزيد من عدوانية الورم الخبيث.

الطرق المعتمدة والنتائج

تعتمد الدراسة على تحليل ثمانين حالة من سرطانات الثدي، غير الألتهابية، ونتائج التقنيات الكيميائية المتقدمة (neoadjuvant) المعتمدة لعلاجها. تم تشخيص الورم بناء على فحوص خزعات (biopsies) ثم عزلها قبل العلاج ومن ثم خضعت المريضات لست دورات من العلاج المكثف والذي يعتمد على إعطاء 75 ملغ/م^2 (75 mg/m^2) من مادة الايبيروبيسين (Epirubicin) و 1200 ملغ/م^2 (1200 mg/m^2) من مادة السيكلوفوسفاميد (Cyclophosphamide) كل 14 يوم. وعند نهاية العلاج، خضعت المريضات لعملية إستئصال للثدي (mastectomy) للتأكد مخبريا من مدى فعالية العلاج. وجرى إعتماد نتائج دراسة الخزعات، ما قبل العلاج، لتحديد حالة العامل TP53.

ومما يلفت النظر هو أن جميع الحالات الـ 15 من الأستجابة الكلية للعلاج كانت من بين الحالات الثمانية والعشرين التي ثبت فيها حدوث طفرة للعامل TP53 في الورم الخبيث. يضاف الى ذلك، أن 9 من 10 حالات من الطفرة في العامل TP53 للأورام الشديدة العدوانية أظهرت تجاوبا كليا مع العلاج وأن وضعية العامل TP53 كانت تشكل مؤشرا دقيقا لمدى نجاح الأستجابة للعلاج.

ولقد تم تحديد العديد من نماذج الطفرات في جينات العامل TP53، منها على سبيل المثال CDC20, TTK, CDKN2A وجينة الخلايا الجذعية PROM1 ولكن التحاليل لم تنجح في مجال تحديد نموذج الانتساخ (Transcription Profile) المميزة لطفرة العامل TP53 التي تميزت بالاستجابة الكاملة للعلاج. وفي حالات عدم الاستجابة للعلاج، كان نموذج الطفرة في العامل TP53 مؤشرا لقصر المدة المتبقية من حياة المريض. بالمقابل، فإن الخمس عشرة مريضة ممن أصبن بورم يتميز بطفرة TP53 مستجيبة، تماثلن الى الشفاء مما يوحي بأن نموذج العلاج الكيميائي من شأنه أن يتخطى خطورة الأصابة التي تصحب عادة الطفرة في العامل TP53.

النتيجة

تظهر نتائج هذه الدراسة أنه في حالة سرطان الثدي غير الالتهابية، تعتبر وضعية العامل TP53 كمؤشر دقيق يسمح بتوقع مردود العلاج الكيميائي المكثف بمزيج الايبيروبيسين- السيكلوفوسفاميد. وإذا ما قارنا بين دقة التوقعات بنجاح هذا العلاج، والنتائج المحبطة للعلاجات الكيميائية الأخرى للأورام الملازمة لطفرة في العامل TP53، فإن العلاج المقترح يتميز بفعالية أفضل لهذا النوع من سرطانات الثدي.
