# Supplementary material for: Exquisite Sensitivity of TP53 Mutant and Basal Breast Cancers to a Dose-Dense Epirubicin−Cyclophosphamide Regimen
Source: PLoS Med. 2007 Mar 20;4(3):e90. doi: 10.1371/journal.pmed.0040090 (PMC1831731; doi:10.1371/journal.pmed.0040090)
Supplement: Alternative Language Abstract S2 [file pmed.0040090.sd002.pdf]

伴TP53突变以及基底组织型乳腺癌对高浓度表柔比星-环磷酰胺化疗方案的特异敏感性

摘　　要

背景：

目前，只有很少的乳腺癌患者能够通过不同的化疗得到彻底治愈。发现能够预示治疗反应的特异标志物对患者选择特殊治疗方案的意义非凡。在细胞株和动物模型中，*TP53*基因对于调节基因组毒性药物的反应至关重要，由于DNA损伤而导致其激活，从而引发细胞完全相反的二种命运：细胞凋亡或细胞周期阻滞。然而在患者中，*TP53*的突变状态和化疗反应之间的关系至今未能明确建立。在乳腺癌中，伴*TP53*突变的患者同样表现出极差的预后。但*TP53*突变究竟导致了疗效降低还是增加了肿瘤的恶性侵袭性还不得而知。

方法和结果：

我们分析了80例确诊后直接应用(新佐剂)化疗方案的非炎症性乳腺癌病例。经治疗前活组织切片检查确诊为乳腺癌后，患者接受6轮高浓度(75mg/m^2)表柔比星（epirubicin）和1200mg/m^{2}环磷酰胺（cyclophosphamide）联合治疗，间期14天。疗程结束后，所有患者都接受了乳房切除术，进一步评估化疗疗效。治疗前的活检样本通过一种高效酵母功能试验和RNA转录组检测，被用以进行*TP53*基因突变检查。我们惊奇地发现，所有15例完全缓解的病例都来自于28例*TP53*突变病例组。在伴*TP53*突变的肿瘤病例，10例经细胞角蛋白免疫组化染色证实为高侵袭性基底组织型中有9例获得了完全缓解。而且只有*TP53*突变状态和基底组织型

可以作为完全缓解的独立的预测标志。在伴*TP53*突变的病例中，我们检测到了不少*TP53*突变相关基因如*CDC20*，*TTK*，*CDKN2A*和干细胞基因*PROM1*的表达，但并不能证实其与化疗反应性的关系。在对化疗无反应的病例中，*TP53*突变预示生存期明显缩短。然而，15例对化疗有反应的*TP53*突变病例预后良好，提示这个化疗方案可以逆转通常情况下与*TP53*突变相关的不良预后。

结论：

我们的研究证实了在非炎症性乳腺癌中，*TP53*的突变状态对于高浓度表柔比星-环磷酰胺化疗方案的疗效是一个关键的预示标志，尤其是基底组织型特别敏感。众所周知，通过化疗获得完全缓解往往预示生存期较长和预后良好，而经其它化疗方案治疗的伴 *TP53*突变和基底组织型乳腺癌，通常预后不良。而我们所运用的高浓度表柔比星-环磷酰胺化疗方案恰恰能在*TP53*突变的乳腺癌患者中取得良好效果，尤其是基底组织型。
