# Supplementary material for: Exquisite Sensitivity of TP53 Mutant and Basal Breast Cancers to a Dose-Dense Epirubicin−Cyclophosphamide Regimen
Source: PLoS Med. 2007 Mar 20;4(3):e90. doi: 10.1371/journal.pmed.0040090 (PMC1831731; doi:10.1371/journal.pmed.0040090)
Supplement: Alternative Language Abstract S3 [file pmed.0040090.sd003.pdf]

# Extrême chimio-sensibilité des cancers du sein ayant une mutation *TP53* ou de type basal à une association dose-intense d'épirubicine et de cyclophosphamide.


**Résumé :**

**Contexte :**
Seule une minorité des patientes ayant un cancer du sein bénéficient pleinement de la chimiothérapie. L'identification de marqueurs pouvant prédire la réponse à un traitement donné serait donc un élément critique pour la prise en charge de ces patientes. Sur des cellules en cultures ou dans des modèles animaux, TP53 joue un rôle critique dans la modulation de la réponse aux agents génotoxiques. Son activation en réponse à des lésions de l'ADN peut entraîner soit l'apoptose, soit l'arrêt du cycle cellulaire, ce qui a des effets opposés sur le devenir de la cellule. Pourtant, à ce jour, les études menées chez l'homme n'ont pas démontré de lien entre *TP53* et la réponse à la chimiothérapie. Il est bien connu que le pronostic des cancers du sein avec mutation de *TP53* est défavorable, mais on ne sait pas si cela est du à une mauvaise réponse aux traitements ou à une plus grande agressivité intrinsèque de ces tumeurs.

**Méthodes et résultats :**
Nous avons étudié 80 cancers du sein non-inflammatoires traités par chimiothérapie néo-adjuvante. Après une biopsie chirurgicale diagnostique, les patientes ont reçu toutes les deux semaines une association d'épirubicine (75mg/m2) et de cyclophosphamide (1200mg/m2) pour un total de 6 cures. A l'issue de ce traitement, toutes les patientes ont eu une mastectomie permettant une analyse fiable de la réponse histo-pathologique. Ces biopsies ont aussi permis de rechercher les mutations de *TP53* à l'aide d'un test fonctionnel dans la levure, et de réaliser des profils d'expression. De manière inattendue, les 15 réponses histo-pathologiques complètes ont toutes été observées dans le groupe des 28 tumeurs avec mutation de *TP53*. De plus, au sein des tumeurs mutées, 9 parmi 10 carcinomes de type basal (définis par la présence en immuno-histochimie des cytokératines basales) ont présenté une réponse complète au traitement. Seuls le statut *TP53* et le sous-type basal étaient des marqueurs prédictifs indépendants de la réponse complète. L'étude d'expression a identifié de nombreux gènes associés à la mutation de *TP53* tels que *CDC20*, *TTK*, *CDKN2A* et le gène de cellules souches *PROM1*, mais n'a pas retrouvé de profil d'expression associé à la réponse complète parmi les tumeurs avec mutation de *TP53*. Chez les patientes avec des tumeurs n'ayant pas répondu au traitement, la présence d'une mutation de *TP53* était associée à une survie plus courte. Pourtant, les 15 patientes présentant une réponse complète ont eu une évolution favorable, suggérant que cette chimiothérapie permet de contrecarrer l'effet délétère de la mutation de *TP53* sur l'évolution ces tumeurs.

**Conclusion :**
Cette étude démontre que, dans les cancers du sein non-inflammatoires, le statut de *TP53* est un marqueur clef de la réponse à une chimiothérapie dose-intense epirubicine/cyclophosphamide, et suggère de plus que les cancers de type basal sont extrêmement sensibles à cette association thérapeutique. Puisque que la réponse histopathologique complète est un facteur majeur de bon pronostic et que les tumeurs avec mutation de *TP53* ont un pronostic particulièrement sombre avec d'autres traitements, cette chimiothérapie pourrait être particulièrement adaptée à ce type de cancers du sein avec mutation de *TP53*, et en particulier de type basal.
