# Supplementary material for: Exquisite Sensitivity of TP53 Mutant and Basal Breast Cancers to a Dose-Dense Epirubicin−Cyclophosphamide Regimen
Source: PLoS Med. 2007 Mar 20;4(3):e90. doi: 10.1371/journal.pmed.0040090 (PMC1831731; doi:10.1371/journal.pmed.0040090)
Supplement: Alternative Language Abstract S4 [file pmed.0040090.sd004.doc]

**Hintergrund**

Nur eine Minderheit aller Brustkrebspatienten profitiert voll von den verschiedenen Chemotherapieprotokollen. Die Identifikation von Prediktivmarkern für das Ansprechen einer Therapie ist somit von großer Bedeutung für die Patientenversorgung. In Zellkulturlinien und Tiermodellen spielt der Marker *TP53* eine entscheidende Rolle bei der Modulation der Zellantwort auf gen-toxische Substanzen, indem seine Aktivierung als Antwort auf DNS-Schäden entweder Apoptose oder den Stopp des Zellzyklus induziert, was zu entgegen­gesetzten Effekten für das Schicksal der Zelle führt. Dennoch haben Studien, die den TP53 Status und das Ansprechen auf Chemotherapien an Patienten untersucht haben bislang keinen zweifellosen Zusammenhang finden können. Für Brusttumoren mit der TP53 Mutation wurde wiederholt ein schlechtes outcome gezeigt, aber ob dies eine schlechte Therapieantwort oder eine stärkere intrinsische Aggressivität des Tumors widerspiegelt ist unbekannt.

**Methoden und Resultate**

Wir haben 80 nicht-inflammatorische Brusttumoren, die mit Standard (neo-adjuvanter) Chemotherapie behandelt wurden analysiert. Die Tumordiagnose wurde vor Beginn der Behandlung durch Biopsien etabliert und die Patienten wurden dann mit 6 Zyklen eines „dose dense“ Chemotherapieprotokolls 75 mg/m^2^ Epirubicin und 1200 mg/m^2^ Cyclophosphamid alle 14 Tage behandelt. Nach Ende der Therapie wurde für alle Patienten eine Mastektomie durchgeführt, die eine Evaluierung der Chemotherapieantwort erlaubte. Die vor Behandlungsbeginn entnommenen Biopsien wurden verwendet, um den *TP53* Status mit Hilfe eines hoch effizienten Hefe-funktions- Assays zu bestimmen und ein RNS Profil zu erstellen. Erstaunlicherweise traten die 15 Fälle die ein komplettes Ansprechen auf die Therapie zeigten unter den 28 *TP53* mutierten Tumoren auf. Zudem erreichten unter den *TP53* Tumoren 9 von 10 der hochaggressiven Basalsubtypen, definiert durch die Basal­zytokeratin­immunhisto­chemie­fär­­bung, ein vollständige Therapieantwort und nur der *TP53* Status und der Basalsubtyp waren unabhängige Prädiktoren eines kompletten Ansprechens. Expressions­analysen identifizierten viele unterschiedliche Mutationen *TP53* assoziierter Gene, einschließlich *CDC20, TTK*, *CDKN2* und das Stammzellgen *PROM1*, aber konnten kein Transkriptionsprofil identifizieren, das mit einem kompletten Therapieansprechen von *TP53* Tumoren assoziiert war. Bei Patienten mit nicht ansprechenden Tumoren, war die *TP53* Mutation mit einer kürzeren Überlebenszeit assoziiert. Dennoch hatten 15 Patienten mit ansprechenden *TP53* Mutantenttumoren ein günstiges Outcome, was nahelegt, dass dieses Chemotherapieprotokoll die schlechte Prognose, die gewöhnlich mit dem *TP53* Mutantenstatus einhergeht, überwinden kann.

**Schlussfolgerung**

Diese Studie zeigt, dass *TP53*für Nicht-inflammatorischen Brusttumoren ein prediktiver Schlüsselfaktor für das Therapieansprechen auf dieses „Dose dense“ Epirubicin-Cyclophosphamid Chemotherapieprotokoll ist und legt des weiteren Nahe, dass diese Assoziation für den Basalsubtyp besonders ausgeprägt ist. Wenn man den gut etablierten prediktiven Wert einer kompletten Therapieantwort für das Langzeitüberleben und die ungünstige Prognose von Basal und *TP53* Mutantentumoren, die mit anderen Therapieprotokollen behandelt werden in Betracht zieht, könnte dieses Chemotherapie­protokoll besonders gut für Brustkrebspatienten mit mutiertem *TP53* besonders diejenigen mit Basalsubtypen geeignet sein.
