# Supplementary material for: Exquisite Sensitivity of TP53 Mutant and Basal Breast Cancers to a Dose-Dense Epirubicin−Cyclophosphamide Regimen
Source: PLoS Med. 2007 Mar 20;4(3):e90. doi: 10.1371/journal.pmed.0040090 (PMC1831731; doi:10.1371/journal.pmed.0040090)
Supplement: Alternative Language Abstract S5 [file pmed.0040090.sd005.pdf]

エビルビシン/シクロフォスファミド併用dose-dense療法に対する*TP53*遺伝子突然変異型・基底細胞型乳癌の高度感受性

要約

背景

現在使用されている乳癌の化学療法から十分に治療利益を得ている患者は少数である。よって、特定の治療法に対する反応を予測するマーカーを同定することは、患者のケアに関して、極めて重要である。培養細胞系やモデル動物の系において、*TP53*は、遺伝子毒性薬物に対する反応調節に重要な役割を果たし、DNA傷害に対するその活性化は、アポトーシスと細胞周期の停止を誘発し、細胞の運命に負の効果をもたらすことが示されている。しかし、ヒトにおける*TP53*変異と化学療法の反応性に関する研究は、今までのところ、明確には示されていない。*TP53*遺伝子突然変異型の乳癌は、予後不良であることが示されているが、その治療反応性の低さや腫瘍の高悪性度の原因は未だ不明である。

方法と所見

我々は、ファーストラインの（ネオアジュバント）化学療法を施行した８０例の非炎症性乳癌を解析した。乳癌の診断は、治療前の生検標本によってなされ、患者は、２週間毎に、６サイクルの75mg/m2エビルビシンと1200mg/m2シクロフォスファミド併用dose-dense療法を受けた。化学療法に引き続き、全患者は、乳房切除術を受けた。この手術検体を用いて、化学療法に対する反応を正確に評価することができた。また治療前の生検標本を用いて、yeast functional assay による*TP53*変異の検出とRNAプロファイル解析を行った。完全奏効した15例はすべて、28例の*TP53*変異型乳癌患者群にみられた。さらに、基底細胞型サイトケラチンによる免疫組織染色によって、*TP53*変異型乳癌のうち、10例中の9例の高悪性度の基底細胞型が完全奏効に至ったことが判明した。よって*TP53*変異型と基底細胞型だけが、完全奏効の独立した予測因子であった。Expression analysisによって、*CDC20, TTK, CDKN2A*を含む多くの*TP53*変異関連遺伝子や幹細胞遺伝子*PROM1*が同定されたが、*TP53*変異型乳癌における完全奏効に相関する転写プロファイルは同定できなかった。治療に反応しない患者群では、*TP53*変異は、全生存期間短縮と有意な相関を示した。また、治療反応性の*P53*変異型乳癌患者15例は、予後良好であった。この化学療法は、*P53*変異型に関連した一般に予後不良な例に有効であることが示唆された。

結論

今回の研究により、非炎症性乳癌において、*P53*変異は、エビルビシン/シクロフォスファミド併用dose-dense療法に対する反応に関し、重要な予測因子であることが判明した。さらに、基底細胞型は、*P53*変異と予後の相関に強く関連していることが示唆された。また完全奏効が長期予後の主要な因子であることと他の治療法による*TP53*変異型基底細胞型乳癌の予後不良を考え合わせると、この化学療法は、*TP53*変異型乳癌、特に基底細胞型の乳癌にとりわけ適した治療法であろう。
