# Supplementary material for: Exquisite Sensitivity of TP53 Mutant and Basal Breast Cancers to a Dose-Dense Epirubicin−Cyclophosphamide Regimen
Source: PLoS Med. 2007 Mar 20;4(3):e90. doi: 10.1371/journal.pmed.0040090 (PMC1831731; doi:10.1371/journal.pmed.0040090)
Supplement: Alternative Language Abstract S6 [file pmed.0040090.sd006.pdf]

Extrema quimiosensibilidade dos cânceres de mama do tipo basal com mutaçao de TP53 à uma associaçao de epirubicina e ciclofosfamida

Resumo

Contexto :
Somente uma minoria dos pacientes tendo um câncer de mama se beneficiam realmente de uma quimioterapia. A identificaçao de marcadores que possam predizer a resposta à um dado tratamento é portanto um elemento essencial para a decisao terapêutica frente à estes pacientes. Em células em culturas ou em modelos animais, o TP 53 tem um papel importante na modulaçao da resposta à drogas genotoxicas. Sua ativaçao em resposta à lesoes do DNA pode provocar ora a apoptose, ora o bloqueio do ciclo celular, com efeitos opostos sobre o futuro da célula. No entanto, os estudos em humanos nao demonstraram ainda claramente a ligaçao entre TP53 e a resposta à quimioterapia. E sabido que os canceres de mama com mutaçao do TP53 têm um mau prognostico, mas nao se sabe se é devido à uma ma resposta aos tratamentos ou à uma grande agressividade intrinseca destes tumores.

Métodos e resultados

Nos estudamos 80 cânceres de mama nao-inflamatorios tratados por quimioterapia néo-adjuvante. Apos uma biopsia cirurgica diagnostica, os pacientes receberam quinzenalmente uma associaçao de epirubicina (75 mg/m2) e ciclofosfamida (1200 mg/m2) num total de 6 infusoes. Apos, todas as pacientes foram mastectomizadas e as peças histologicamente analisadas. As biopsias iniciais nos permitiram pesquisar as mutaçoes de TP53 com ajuda de um teste funcional em levedura, e realisar os perfils de expressao. De maneira espantosa, as 15 respostas histopatologicas completas foram todas observadas dentro do grupo de 28 tumores com mutaçao de TP53. Além disso, entre os tumores mutados, 9 entre 10 carcinomas do tipo basal (definidos pela presença imunohistoquimica de citoqueratinas basais) responderam completamente ao tratamento. Somente o estatuto TP53 e o tipo basal foram marcadores preditivos independentes da resposta completa. O estudo de expressao identificou numerosos genes associados à mutaçao de TP53 tanto quanto CDC20, TTK, CDKN2A e o gene das células tronco PROM1, mas nao mostrou o perfil de expressao associado à resposta completa entre os tumores com mutacao de TP53. Nas pacientes que apresentaram tumores que nao responderam ao tratamento, a presença de uma mutaçao de TP53 foi associada à uma sobrevida menor. No entanto, as 15 pacientes com resposta completa tiveram um evoluçao favoravel , sugerindo que esta quimioterapia pode opor-se totalmente ao efeito deleterio da mutaçao de TP53 nestes tumores.

Conclusao

Este estudo demonstra que entre os cânceres de mama nao-inflamatorios, o estatuto TP53 é um marcador chave da resposta à uma quimioterapia em altas doses de epirubicina – ciclofosfamida, e sugere ainda que os cânceres de mama do tipo basal sao extremamente sensiveis à esta associaçao terapêutica. Ja que a resposta histopatologica completa é um fator maior de bom prognostico e que os tumores com mutaçao de TP53 sao conhecidos por seus prognosticos sombrios com outros tratamentos, esta quimioterapia podera ser particularmente

adaptada à estes cânceres de mama com mutaçao de TP53 e em particular, os cânceres do tipo basal.
