# Supplementary material for: Exquisite Sensitivity of TP53 Mutant and Basal Breast Cancers to a Dose-Dense Epirubicin−Cyclophosphamide Regimen
Source: PLoS Med. 2007 Mar 20;4(3):e90. doi: 10.1371/journal.pmed.0040090 (PMC1831731; doi:10.1371/journal.pmed.0040090)
Supplement: Alternative Language Abstract S7 [file pmed.0040090.sd007.pdf]

Chimiosensibilitate ridicată la un tratament asociind epirubicină şi ciclofosfamidă in cancerele de sân de tip bazal cu o mutație in gena *TP53*

Rezumat

Context

Doar o minoritate de paciente cu un cancer de sân beneficiază de diferite tratamente de chimioterapie._Identificarea marcherilor care pot prezice răspunsul la un anumit tratament este deci un element esențial pentru individualizarea tratamentului la aceste paciente. Pe diferite modele de studiu de culturi celulare sau la şoarece, gena *TP53* joacă un rol important în modularea răspusului la tratamentul cu droguri chimioterapeutice. Activarea sa ca răspuns la diferite leziuni pe ADN poate să inducă fie apoptoza celulară fie oprirea ciclului celular, cu efecte opuse pentru evoluția acestor celule. Totuşi, studiile făcute la om până în prezent, nu au demonstrat clar legătura intre TP53 si răspunsul la tratament . Este binecunoscut prognosticul negativ al pacientelor cu un cancer de sân cu o mutație in gena *TP53*, dar nu se ştie până in prezent dacă acest prognostic este datorat unui răspuns slab la tratament sau a unei mari agresivitați intrinsece a acestor tumori.

Metode si rezultate

In acest studiu am analizat 80 cancere de sân fără inflamație, tratate cu o chimioterapie neoadjuvantă. După o biopsie chirurgicală pentru diagnostic, toate pacientele au primit timp de 2 săptămâni o asociație de epirubicină (75mg/m2) si ciclofosfamidă (1200mg/m2), pe un total de 6 cure. La sfârşitul acestui tratament toate pacientele au avut o mastectomie, iar răspunsul histologic a putut să fie analizat. Biopsiile inițiale au permis cautarea mutațiilor în gena *TP53* cu ajutorul unui test funcțional realizat pe drojdie, precum si realizarea profilelor de expresie genică. În mod surprinzător cele 15 răspunsuri histologice complete au fost observate în totalitate în grupul de 28 tumori cu o genă *TP53* mutată. În plus în cadrul tumorilor mutate, 9 pe 10 carcinoame de tip bazal (marcaj pozitiv in imunohistochimie pentru citokeratinele bazale) au răspuns complet la tratament. Doar statutul genei *TP53* si tipul bazal erau marcheri predictivi independenți pentru un raspuns complet. Studiile de expresie genică au identificat numeroase gene asociate cu mutația in *TP53* cum a fi *CDC20*, *TTK*, *CDKN2A,* dar si gena *PROM1* specifică celulelor suşă. La pacienții cu tumori care nu au răspuns la tratament prezența unei mutații în gena *TP53* a fost asociată la o durată de viață mai scurtă. Totuşi 15 pacienți cu un răspuns complet au avut o evoluție favorabilă ceea ce demonstrează că această chimioterapie poate să contracareze în întregime efectul nefast al mutației genei *TP53* în aceste tumori.

Concluzie :

Acest studiu demonstrează că în cancere de sân ne-inflamatoare statutul genei *TP53* este un marcher cheie al răspunsului la o chimioterapie în doze ridicate de epirubicină şi ciclofosfamida şi în plus sugerează că, tumorile de sân de tip bazal sunt foarte sensibile la această asociație terapeutică. Ținând cont că răspunsul histopatologic complet este un factor major de prognostic pozitiv şi că tumorile cu

mutația în gena *TP53* sunt cunoscute pentru prognosticul lor negativ la alte tratamente, această chimoterapie ar putea să fie adaptată în particular la cancere de sân cu o mutație în gena *TP53* şi în particular în cancere de sân de tip bazal.
