# Supplementary material for: Exquisite Sensitivity of TP53 Mutant and Basal Breast Cancers to a Dose-Dense Epirubicin−Cyclophosphamide Regimen
Source: PLoS Med. 2007 Mar 20;4(3):e90. doi: 10.1371/journal.pmed.0040090 (PMC1831731; doi:10.1371/journal.pmed.0040090)
Supplement: Alternative Language Abstract S8 [file pmed.0040090.sd008.pdf]

Los cánceres de mama de tipo basal con mutación *TP53*, son extremadamente sensibles a la quimioterapia con epirubicina y citofosfamida.

Resumen:

Introducción:

Sólo una mínima parte de los pacientes con cáncer de mama responden a los actuales régimenes de quimioterapia. Por ello, la identificación de marcadores pronóstico de la respuesta a un tratamiento determinado puede ser un elemento fundamental a la hora de tratar a estos pacientes.

Tanto en células en cultivo como en modelos animales, la TP53 juega un papel fundamental en la regulación de la respuesta a los fármacos genotóxicos. Su activación, en respuesta a ciertas lesiones del ADN, puede derivar en apoptosis o en parada del ciclo celular. Sin embargo, los estudios realizados en el hombre no han demostrado claramente, de momento, la relación entre TP53 y la respuesta a la quimioterapia. Es bien sabido, que el pronóstico de los cánceres de mama con mutación *TP53* es desfavorable pero lo que no se sabe todavía, es si es debido a una mala respuesta al tratamiento o a una mayor agresividad intrínseca de estos tumores.

Métodos y resultados:

Hemos estudiado 80 cánceres de mama no inflamatorios tratados con quimioterapia neo-adjuvante. Uma vez realizado el diagnóstico de la biopsia quirúrgica, los pacientes recibieron una asociación de epirubicina(75mg/m2) y de cyclofosfamida (1200mg/m2) cada dos semanas hasta un total de 6 ciclos. Al término de dicho tratamiento, todos los pacientes sufrieron una mastectomia sobre la cual, la respuesta histopatológica pudo ser analizada con precisión.

Las biopsias pre-tratamiento permitieron la búsqueda de mutaciones en *TP53* a través de un test funcional sobre la levadura y la realización de perfiles de expresión (de ARN). De manera sorprendente, todas las 15 respuestas histopatológicas completas correspondían al grupo de 28 tumores con mutación *TP53*. Asimismo, dentro de los tumores mutados, 9 de los10 carcinomas de tipo basal (definidos por la presencia inmunohistoquímica de citoqueratinas basales) respondieron completamente al tratamiento. Los únicos marcadores predictivos estadísticamente independientes de una respuesta completa fueron la presencia o ausencia de mutación de *TP53* y correspomder a un carcinoma de tipo basal. El estudio de la expresión, identificó un gran número de genes asociados a la mutación de *TP53*, tales como, *CDC20*, *TTK*, *CDKN2A* y el gen de las células madre *PROM1*, pero no encontró el perfil de expresión asociado a la respuesta completa de los tumores con mutación *TP53*. Entre los pacientes que no respondieron al tratamiento, la presencia de *TP53* se asoció a una menor supervivencia. Sin embargo, los 15 pacientes que respondieron completamente al tratamiento, tuvieron una evolución favorable que sugiere que la aplicación de esta quimioterapia puede contrarrestar totalmente el efecto nefasto de la mutación *TP53* en estos tumores.

Conclusión :

Este estudio demuestra que, en los cánceres de mama no inflamatorios, la presencia de la mutación *TP53* es un marcador clave en la respuesta a la quimioterapia a dosis intensas de epirubicine-ciclofosfamida, y sugiere además, que los cánceres de tipo basal son extremadamente sensibles a esta asociación terapeútica. Teniendo en cuenta que la respuesta histopatológica completa es un factor de buen pronóstico y que los tumores con mutación *TP53* son conocidos por su pronóstico sombrío con otros tratamientos, esta quimioterapia podría aplicarse a los cánceres con mutación *TP53*, y en particular a aquellos de tipo basal.
