# Supplementary material for: Exquisite Sensitivity of TP53 Mutant and Basal Breast Cancers to a Dose-Dense Epirubicin−Cyclophosphamide Regimen
Source: PLoS Med. 2007 Mar 20;4(3):e90. doi: 10.1371/journal.pmed.0040090 (PMC1831731; doi:10.1371/journal.pmed.0040090)
Supplement: Dataset S1 — (290 KB PDF) [file pmed.0040090.sd009.doc]

**Bio-informatic methods**

**T tests and F tests**

The method used also evaluates the number N’ of probe sets (N’ first probe sets ordered by their p-value from the univariate test) for which the number of FDs is less than 10 (with a probability of 90%). The below table recapitulates the number of probe sets (2^nd^ column) with a p-value less than a given threshold (3^rd^ column) obtained from each test (1^st^ column). The 4^th^ column indicates the set of samples used for the test and the 5^th^ column indicates which test was used. The 6^th^ and 7^th^ columns give the N and N’ from each test, respectively.

| **Conditions tested** | **Number of significant genes** | **p-value threshold** | **Set** | **Test** | **N for which FDR < 10% (prob. 90%)** | **N’ for which FD < 10**  **(prob. 90%)** |
| --- | --- | --- | --- | --- | --- | --- |
| TP53 mutant vs wild type | 1,599 | 0.001 | S1 | T-test | 2,313 | 1,143 |
| C1 vs C2 | 909 | 0.001 | S1+S2 | T-test | 1,214 | 524 |
| C1 vs C3 | 839 | 0.001 | S1+S2 | T-test | 1,271 | 478 |
| C2 vs C3 | 3,129 | 0.05 | S1+S2 | T-test | 137 | 120 |
| C1 vs C2 vs C3 | 1,400 | 0.001 | S1+S2 | F-test | 1,975 | 851 |

The subgroups C2 (n = 7) and C3 (n = 9) are significantly smaller than the group C1 (n = 21) reducing the power of a statistical test between these 2 groups compared to test comparing C2 and C1 or C3 and C1. If we reduce the p value threshold for the C2 vs C3 test we take into account this difference in statistical power and obtain more genes that overlap with the C1 vs C2 and C1 vs C3 tests. Genes that are shown in Table S4 and Table S5 (C2 and C3 gene lists, respectively) are highly significant (P < 0.001) in C2 vs C1 and C3 vs C1, respectively and from an ANOVA test while yielding, for some genes, a higher but still significant p values (p < 0.05). The cluster class-related lists (e.g. C1 vs C2) were further filtered by demanding a geometric mean intensity of at least 100 in at least 1 class.

**T-tests and Wilcoxon tests of TP53 status**

To confirm that the expression of the 12 genes was significantly different between  *TP53* mutant and  *TP53* wild type samples, we performed independent Wilcoxon tests and T-tests for each of the 12 genes, using samples from set S1, as well as S2+S3 samples (see Table A below). As both tests gave a p-value higher or equal to 0.5 for gene MAFF on set S1, it was eliminated leaving 11 genes from further analyses.

For each predictor and each of the sub-groups, we obtained a contingency table of the following form :

|  | |  | | |  | | |
| --- | --- | --- | --- | --- | --- | --- | --- |
|  |  |  |  |  |  |
|  |  |  |  |  |  |  |  |
|  |  |  |  |  |  |  |
|  |  |  |  |  |  |  |  |
|  | |  | | |  | | |
|  |  |  |  |  |  |
|  |  |  |  |  |  |  |  |
|  |  |  |  |  |  |  |
|  |  |  |  |  |  |  |  |

|  |  |  |
| --- | --- | --- |
|  |  |  |
|  |  |  |

g form :

|  | |  | | |  | | |
| --- | --- | --- | --- | --- | --- | --- | --- |
|  |  |  |  |  |  |
|  |  |  |  |  |  |  |  |
|  |  |  |  |  |  |  |
|  |  |  |  |  |  |  |  |

|  |  |  |
| --- | --- | --- |
|  |  |  |
|  |  |  |

|  |  |  |  |  | |
| --- | --- | --- | --- | --- | --- |
|  |  |  |  |  |  |
|  |  |  |  |  |  |
|  |  |  |  |  |  |
|  |  |  |  |  |  |
|  |  |  |  |  |  |
|  |  |  |  |  |  |
|  |  |  |  |  |  |
|  |  |  |  |  |  |
|  |  |  |  |  |  |
|  |  |  |  |  |  |
|  |  |  |  |  |  |
|  |  |  |  |  |  |

|  |  | | |  | | |
| --- | --- | --- | --- | --- | --- | --- |
|  |  |  |  |  |  |  |
|  |  |  |  |  |  |  |
|  |  |  |  |  |  |  |
|  |  |  |  |  |  |  |
|  |  |  |  |  |  |  |
|  |  |  |  |  |  |  |
|  |  |  |  |  |  |  |
|  |  |  |  |  |  |  |
|  |  |  |  |  |  |  |
|  |  |  |  |  |  |  |
|  |  |  |  |  |  |  |
|  |  |  |  |  |  |  |
|  |  |  |  |  |  |  |

| **Wilcoxon** | all | training | validation | ESR1 pos | ESR1 pos, training | ESR1 pos, validation | ESR1 neg |
| --- | --- | --- | --- | --- | --- | --- | --- |
| ESR1 | 1,90653E-09 | 8,08194E-05 | 4,03554E-05 | 0,000287488 | 0,114647873 | 0,003091947 | 0,899787169 |
| CDC20 | 1,20963E-08 | 0,00387398 | 7,95223E-06 | 0,000683913 | 0,141858142 | 0,00476952 | 0,067436911 |
| ADM | 2,30102E-06 | 0,000402899 | 0,01193199 | 0,003943367 | 0,013186813 | 0,100705 | 0,388525833 |
| PROM1 | 0,00017294 | 9,96995E-05 | 0,030498653 | 0,247437235 | 0,02967033 | 0,92027639 | 0,268635984 |
| p14 | 0,001412908 | 0,006905204 | 0,212988229 | 0,818762397 | 0,316483516 | 0,508868278 | 0,078686531 |
| RIS1 | 0,001522702 | 0,199421431 | 0,022023261 | 0,028274104 | 0,659340659 | 0,08329617 | 0,70513834 |
| TMSNB | 0,001595088 | 0,159550055 | 0,009140371 | 0,952774001 | 0,291208791 | 0,421594527 | 0,048647005 |
| KRT7 | 0,002282679 | 0,071228983 | 0,019125492 | 0,015169159 | 0,225274725 | 0,062631936 | 0,157008209 |
| p16 | 0,002899508 | 0,002796006 | 0,420833553 | 0,835767191 | 0,103296703 | 0,244567916 | 0,134488006 |
| TTK | 0,010024224 | 0,400102732 | 0,091290947 | 0,913111397 | 0,379120879 | 0,763981346 | 0,613408331 |
| PHLDA2 | 0,02135332 | 0,025691943 | 0,367791157 | 0,085413556 | 0,078021978 | 0,393353492 | 0,91384588 |
| MAFF | 0,180376649 | 0,183984607 | 0,461769484 | 0,300423645 | 0,103296703 | 0,952114742 | 0,587357722 |
|  |  |  |  |  |  |  |  |
|  |  |  |  |  |  |  |  |
| **T-TEST** | all | training | validation | ESR1 pos | ESR1 pos, training | ESR1 pos, validation | ESR1 neg |
| ESR1 | 2,75867E-10 | 2,09819E-06 | 9,69356E-05 | 0,000655241 | 0,100607639 | 0,014154268 | 0,897167904 |
| CDC20 | 1,86481E-09 | 0,002238702 | 1,56027E-05 | 0,000607709 | 0,166673491 | 0,00307315 | 0,024420313 |
| ADM | 3,68304E-06 | 0,000574741 | 0,037071487 | 0,015232342 | 0,004941028 | 0,204379249 | 0,703865008 |
| PROM1 | 0,000224377 | 0,000987438 | 0,090932312 | 0,421330363 | 0,010542919 | 0,953833625 | 0,280919634 |
| p14 | 0,002091267 | 0,01362518 | 0,090880246 | 0,428289511 | 0,267386766 | 0,972379301 | 0,094720426 |
| RIS1 | 0,002219626 | 0,220217527 | 0,025807233 | 0,048200858 | 0,460215113 | 0,104779172 | 0,573866793 |
| TMSNB | 0,001130856 | 0,161474548 | 0,010281512 | 0,764850522 | 0,21692097 | 0,362228802 | 0,019325681 |
| KRT7 | 0,272299 | 0,603731467 | 0,228395178 | 0,053657445 | 0,189273799 | 0,167878083 | 0,276815901 |
| p16 | 0,007215172 | 0,00375052 | 0,297103623 | 0,729294897 | 0,090888918 | 0,490787824 | 0,189952041 |
| TTK | 0,004809341 | 0,524734185 | 0,045691351 | 0,730381526 | 0,294260517 | 0,405252376 | 0,79309745 |
| PHLDA2 | 0,025323754 | 0,038245662 | 0,365088279 | 0,101585353 | 0,150632085 | 0,453738642 | 0,571841028 |
| MAFF | 0,577612377 | 0,298397514 | 0,9841202 | 0,870387391 | 0,046593861 | 0,555521971 | 0,464198143 |
