# Supplementary figures and images for: Exquisite Sensitivity of TP53 Mutant and Basal Breast Cancers to a Dose-Dense Epirubicin−Cyclophosphamide Regimen
Source: PLoS Med. 2007 Mar 20;4(3):e90. doi: 10.1371/journal.pmed.0040090 (PMC1831731; doi:10.1371/journal.pmed.0040090)

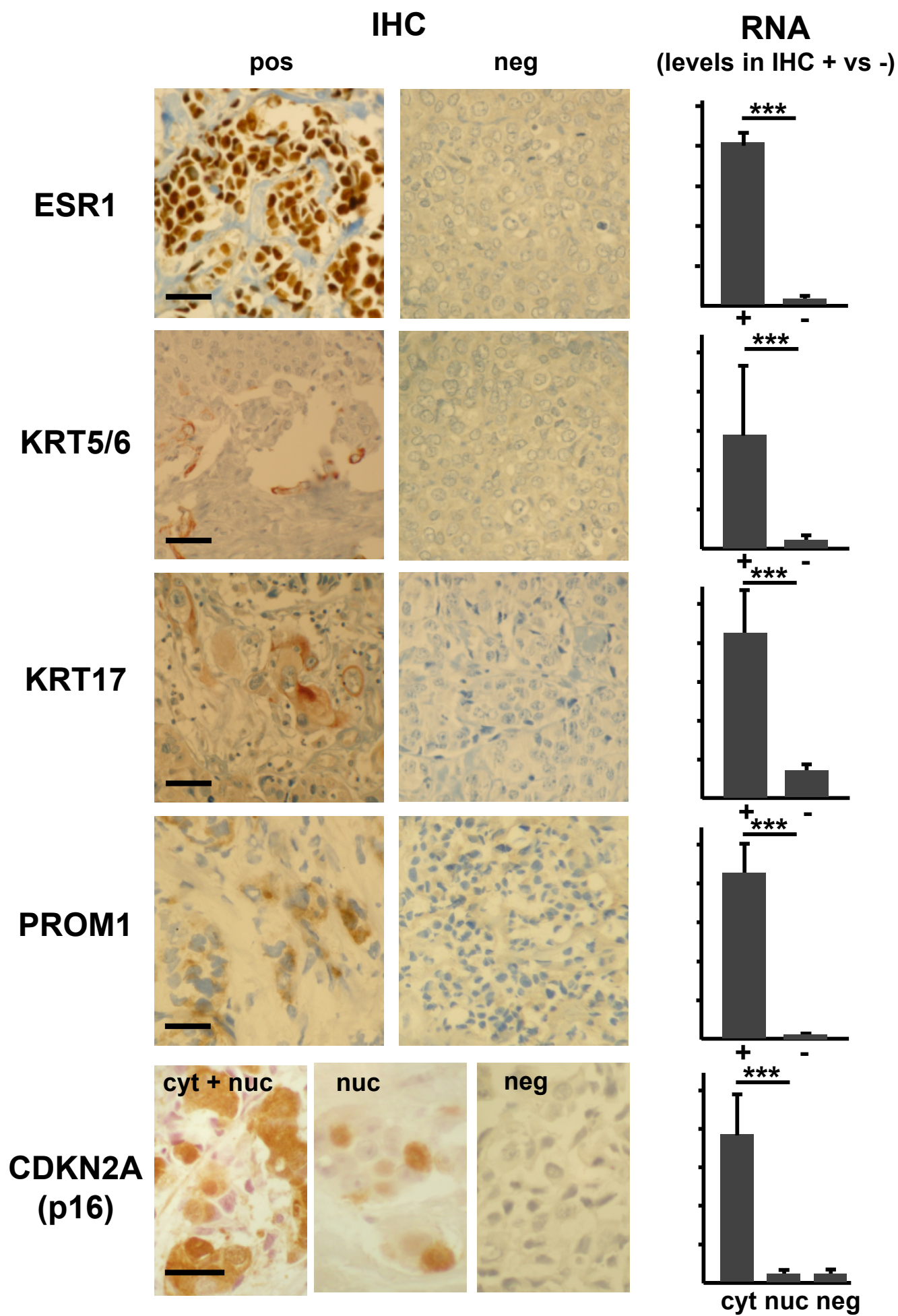

Supplement: Figure S1 — Left: example of tumors scored positive or negative by immunohistochemistry. Right: mean RNA levels for tumors positive and negative for ESR1, KRT5, KRT17, PROM1, and CDKN2A (p16). Pos, positive; neg, negative; cyt, cytoplasmic; nuc, nuclear; ***, p < 0.001 when comparing the levels of mRNA between IHC+ and IHC− tumors. Bars indicate 50 μm. (1.1 MB PDF) [file pmed.0040090.sg001.pdf]
