# Supplementary material for: Exquisite Sensitivity of TP53 Mutant and Basal Breast Cancers to a Dose-Dense Epirubicin−Cyclophosphamide Regimen
Source: PLoS Med. 2007 Mar 20;4(3):e90. doi: 10.1371/journal.pmed.0040090 (PMC1831731; doi:10.1371/journal.pmed.0040090)
Supplement: Table S2 — FASAY, functional analysis of separated allele in yeast [23,24] codon 72; R, arginine; P, proline. (28 KB PDF) [file pmed.0040090.st002.pdf]

patient	FASAY % mutant	*TP53* codon 72	*TP53* mutation	Dominant Negative (IARC)	pathological response
P3	75	P	R175H (G/A)	+	comp
P4	98	R	V147G (T/G)		comp
P11	36	P	R273H (G/A)	+	comp
P23	68	R	T284P (A/C)		comp
P42	71	R	S241F (C/T)	+	comp
P46	19	R	228 (delGT) frameshift		comp
P50	38	P	R248Q (G/A)	+	comp
P51	74	R	R273H (G/A)	+	comp
P63	90	R	R273H (G/A)	+	comp
P67	81	R	R175H (G/A)	+	comp
P69	58	R	del (252 - 258)Q		comp
P71	89	R	del (168 - 174)		comp
P73	98	R	del (225 - 331)		comp
P77	87	R	N239D (A/G)		comp
P78	34	R	P190S (C/T)		comp
P10	23	R	157 ins C frameshift		incomp
P15	70	R	R248Q (G/A)	+	incomp
P21	20	R	Q317stop (C/T)		incomp
P26	88	R	R273H (G/A)	+	incomp
P31	62	R	H193R (A/G)	+	incomp
P37	82	R	R175H (G/A)		incomp
P47	70	R	del (74 - 93)		incomp
P55	63	R	R248W (C/T)	+	incomp
P60	66	R	E285K (G/A)		incomp
P64	96	R	Y220C (A/G)	+	incomp
P68	22	P	Q144stop (C/T)		incomp
P72	54	R	R273H (G/A)	+	incomp
P82	30	R	del (130 - 134)		incomp
