# Supplementary material for: Exquisite Sensitivity of TP53 Mutant and Basal Breast Cancers to a Dose-Dense Epirubicin−Cyclophosphamide Regimen
Source: PLoS Med. 2007 Mar 20;4(3):e90. doi: 10.1371/journal.pmed.0040090 (PMC1831731; doi:10.1371/journal.pmed.0040090)
Supplement: Table S3 — Probe set, ratios of mean values, p-values, gene symbol, and gene name are indicated. Class specific gene lists were generated by the intersection of all group-wise t-tests with an F-test of all three classes (i.e., C1-specific genes, intersection of C1 versus C2, C1 versus C3 and F test, etc.). (68 KB PDF) [file pmed.0040090.st003.pdf]

Probe set	C1/C2	C1vC2 p-value	C1/C3	C1vC3 p-value	Gene symbol	Description
205225_at	**16,57**	p < 1e-07	**15,85**	p < 1e-07	ESR1	estrogen receptor 1
219197_s_at	**11,31**	1,04E-05	**5,94**	0,0002018	SCUBE2	signal peptide, CUB domain, EGF-like 2
209604_s_at	**8,61**	p < 1e-07	**3,27**	2,20E-06	GATA3	GATA binding protein 3
203963_at	**8,37**	2,00E-07	**8,30**	p < 1e-07	CA12	carbonic anhydrase XII
209603_at	**7,64**	p < 1e-07	**4,24**	1,40E-06	GATA3	GATA binding protein 3
209602_s_at	**6,96**	2,00E-07	**3,03**	0,0001004	GATA3	GATA binding protein 3
205696_s_at	**6,17**	1,01E-05	**6,17**	1,20E-06	GFRA1	GDNF family receptor alpha 1
210735_s_at	**6,06**	2,58E-05	**4,39**	7,17E-05	CA12	carbonic anhydrase XII
204508_s_at	**5,88**	8,00E-07	**5,07**	4,00E-07	CA12	carbonic anhydrase XII
203571_s_at	**5,38**	7,60E-06	**3,16**	0,0001853	C10orf116	chromosome 10 open reading frame 11
203929_s_at	**5,09**	0,0003715	**4,01**	0,0005628	MAPT	microtubule-associated protein tau
209460_at	**5,00**	0,000177	**3,60**	0,000537	ABAT	4-aminobutyrate aminotransferase
214053_at	**4,05**	0,0001311	**3,01**	0,000637		CDNA FLJ44318 fis, clone TRACH300(
211712_s_at	**3,92**	6,00E-07	**2,33**	0,00078	ANXA9	annexin A9 /// annexin A9
210652_s_at	**3,68**	7,18E-05	**3,04**	0,0002142	C1orf34	chromosome 1 open reading frame 34
204776_at	**3,20**	0,0019947	**2,92**	0,001266	THBS4	thrombospondin 4
218692_at	**3,10**	1,10E-06	**3,17**	3,20E-06	FLJ20366	hypothetical protein FLJ20366
210085_s_at	**3,06**	1,20E-06	**1,90**	0,0013326	ANXA9	annexin A9
212195_at	**2,80**	0,0011831	**2,59**	0,000916	IL6ST	interleukin 6 signal transducer (gp130,
205116_at	**2,68**	0,000459	**2,31**	0,0007733	LAMA2	laminin, alpha 2 (merosin, congenital m
200810_s_at	**2,62**	3,37E-05	**1,77**	0,0015435	CIRBP	cold inducible RNA binding protein
206338_at	**2,59**	5,02E-05	**1,87**	0,0006175	ELAVL3	ELAV (embryonic lethal, abnormal visio
217838_s_at	**2,50**	0,0002818	**1,94**	0,0012908	EVL	Enah/Vasp-like
213234_at	**2,42**	6,80E-06	**1,82**	0,0004548	KIAA1467	KIAA1467 protein
209769_s_at	**2,41**	0,0003829	**2,01**	0,0009563	GP1BB	glycoprotein Ib (platelet), beta polypepti
205050_s_at	**2,37**	3,98E-05	**2,10**	9,70E-06	MAPK8IP2	mitogen-activated protein kinase 8 inter
221934_s_at	**2,31**	2,00E-06	**1,64**	0,0005998	FLJ10496	hypothetical protein FLJ10496
215304_at	**2,30**	0,0002848	**2,24**	0,0001244		Human clone 23948 mRNA sequence
206278_at	**2,27**	8,68E-05	**1,93**	0,0002488	PTAFR	platelet-activating factor receptor
208682_s_at	**2,22**	0,0001352	**1,74**	0,0009878	MAGED2	melanoma antigen, family D, 2
203842_s_at	**2,20**	0,0002832	**1,98**	0,0002772	MAPRE3	microtubule-associated protein, RP/EB
204378_at	**2,19**	3,00E-07	**1,60**	7,73E-05	BCAS1	breast carcinoma amplified sequence 1
203670_at	**2,19**	0,0001633	**1,78**	0,000353	TTLL3	tubulin tyrosine ligase-like family, memb
207306_at	**2,18**	6,89E-05	**1,83**	0,0002596	TCF15	transcription factor 15 (basic helix-loop-
40016_g_at	**2,15**	4,30E-06	**1,75**	7,14E-05	KIAA0303	KIAA0303 protein
217696_at	**2,14**	0,0003288	**1,85**	0,0003921	FUT7	fucosyltransferase 7 (alpha (1,3) fucosy
205354_at	**2,14**	0,0001865	**2,08**	5,83E-05	GAMT	guanidinoacetate N-methyltransferase
205212_s_at	**2,13**	9,68E-05	**1,75**	0,0002644	CENTB1	centaurin, beta 1
206416_at	**2,12**	0,000148	**1,85**	0,0002187	ZNF205	zinc finger protein 205
222125_s_at	**2,11**	0,0001055	**2,00**	0,0005216	PH-4	hypoxia-inducible factor prolyl 4-hydrox
210684_s_at	**2,10**	0,0002629	**1,91**	0,0001535	DLG4	discs, large homolog 4 (Drosophila)
216289_at	**2,10**	0,0002215	**1,84**	0,0002813	GPR144	G protein-coupled receptor 144
215616_s_at	**2,09**	1,00E-06	**1,75**	4,70E-06	JMJD2B	jumonji domain containing 2B
209730_at	**2,07**	5,50E-05	**1,92**	2,48E-05	SEMA3F	sema domain, immunoglobulin domain
208868_s_at	**2,06**	4,74E-05	**1,55**	0,0010661	GABARAPL1	GABA(A) receptor-associated protein lil
205141_at	**2,05**	0,0004125	**1,91**	0,0005375	ANG	angiogenin, ribonuclease, RNase A fam
204862_s_at	**2,05**	0,0004588	**1,70**	0,0018047	NME3	non-metastatic cells 3, protein expresse
211692_s_at	**2,04**	5,20E-05	**1,92**	1,91E-05	BBC3	BCL2 binding component 3 /// BCL2 bir
217700_at	**2,02**	0,0002211	**1,73**	0,0005293		
213550_s_at	**2,01**	0,0004628	**2,22**	2,33E-05	PRO1580	hypothetical protein PRO1580
215844_at	**2,00**	0,0001526	**1,77**	0,0002054	TNPO2	transportin 2 (importin 3, karyopherin be
214122_at	**2,00**	0,000211	**1,67**	0,0013169		
221946_at	**1,99**	9,58E-05	**1,60**	0,0013917	C9orf116	chromosome 9 open reading frame 116
206328_at	**1,99**	0,0002984	**1,88**	0,0002345	CDH15	cadherin 15, M-cadherin (myotubule)
201841_s_at	**1,96**	0,0020072	**1,75**	0,0013311	HSPB1	heat shock 27kDa protein 1
217058_at	**1,93**	0,0002015	**1,63**	0,0016867	GNAS	GNAS complex locus
205451_at	**1,91**	0,0008512	**1,80**	0,0002204	MLLT7	myeloid/lymphoid or mixed-lineage leuk
204482_at	**1,91**	0,0018012	**1,96**	0,0003043	CLDN5	claudin 5 (transmembrane protein delet
209697_at	**1,90**	0,0008172	**1,78**	0,0004519		
206650_at	**1,87**	0,0003576	**1,75**	0,0002134	IQCC	IQ motif containing C
208141_s_at	**1,87**	0,0004225	**1,79**	0,0001356	MGC4293	hypothetical protein MGC4293 /// hypot
204878_s_at	**1,85**	0,0001578	**1,65**	0,0001033	TAO1	thousand and one amino acid protein ki
204231_s_at	**1,84**	0,0002371	**1,89**	2,65E-05	FAAH	fatty acid amide hydrolase
220705_s_at	**1,84**	0,0003755	**1,53**	0,0015459	ADAMTS7	a disintegrin-like and metalloprotease (r
205074_at	**1,84**	1,80E-05	**1,50**	0,0009569	SLC22A5	solute carrier family 22 (organic cation t
213690_s_at	**1,83**	0,0005455	**1,70**	0,000562	PTOV1	prostate tumor overexpressed gene 1
204876_at	**1,82**	4,75E-05	**1,64**	6,86E-05	KIAA0296	KIAA0296 gene product

216345_at	**1,81**	0,0014811	**1,70**	0,0008961	KIAA0913	KIAA0913
211248_s_at	**1,81**	5,48E-05	**1,63**	0,0001223	CHRD	chordin
208102_s_at	**1,81**	0,0003824	**1,68**	0,0001942	PSD	pleckstrin and Sec7 domain containing
221013_s_at	**1,79**	0,0020081	**1,87**	0,0001043	APOL2	apolipoprotein L, 2 /// apolipoprotein L,
204104_at	**1,79**	7,33E-05	**1,72**	1,42E-05	SNAPC2	small nuclear RNA activating complex,
202700_s_at	**1,78**	0,0005387	**1,78**	0,0001181	KIAA0792	KIAA0792 gene product
201452_at	**1,77**	0,0005989	**1,57**	0,0005311	RHEB	Ras homolog enriched in brain
205730_s_at	**1,77**	0,0006504	**1,67**	0,000342	ABLIM3	actin binding LIM protein family, membe
213319_s_at	**1,75**	0,0005517	**1,55**	0,0008811		
219849_at	**1,75**	0,0001031	**1,52**	0,0005805		
220755_s_at	**1,74**	0,001477	**1,78**	1,05E-05	C6orf48	chromosome 6 open reading frame 48
221163_s_at	**1,74**	0,0006284	**1,56**	0,0009569	WBSCR14	Williams Beuren syndrome chromosom
213651_at	**1,74**	0,0001063	**1,49**	0,0016783	PIB5PA	phosphatidylinositol (4,5) bisphosphate
216821_at	**1,74**	0,0002177	**1,49**	0,0024253		
205201_at	**1,73**	0,0004063	**1,57**	0,001131		
215712_s_at	**1,72**	0,0008733	**1,76**	3,41E-05	IGFALS	insulin-like growth factor binding proteir
201354_s_at	**1,72**	0,0005304	**1,54**	0,0008807		
214269_at	**1,72**	0,0009946	**1,67**	0,0002871	FLJ22269	hypothetical protein FLJ22269
209224_s_at	**1,71**	0,001266	**1,59**	0,0009895	NDUFA2	NADH dehydrogenase (ubiquinone) 1 a
205441_at	**1,70**	0,0017104	**1,71**	7,08E-05	FLJ22709	hypothetical protein FLJ22709
216611_s_at	**1,70**	0,0007205	**1,69**	8,53E-05	SLC6A2	solute carrier family 6 (neurotransmitter
202312_s_at	**1,69**	0,0004789	**1,49**	0,0012541	COL1A1	collagen, type I, alpha 1
210025_s_at	**1,69**	0,0005205	**1,46**	0,0021124	CARD10	caspase recruitment domain family, me
215045_at	**1,69**	0,0015614	**1,51**	0,0014933	TNRC4	trinucleotide repeat containing 4
202488_s_at	**1,68**	0,001561	**1,97**	1,44E-05	FXYD3	FXYD domain containing ion transport r
212925_at	**1,67**	8,97E-05	**1,48**	0,0007695	C19orf21	chromosome 19 open reading frame 21
211512_s_at	**1,66**	0,0003242	**1,58**	8,18E-05	OGFR	opioid growth factor receptor
220024_s_at	**1,66**	0,0004698	**1,49**	0,0008219	PRX	periaxin
205211_s_at	**1,64**	0,0001279	**1,59**	4,96E-05		
216835_s_at	**1,64**	0,0008615	**1,80**	5,20E-06	DOK1	docking protein 1, 62kDa (downstream
218262_at	**1,64**	0,0002735	**1,58**	0,0002368	FLJ22318	hypothetical protein FLJ22318
217937_s_at	**1,62**	0,0001506	**1,42**	0,0016306	HDAC7A	histone deacetylase 7A
209280_at	**1,61**	0,0005154	**1,43**	0,0002125	MRC2	mannose receptor, C type 2
200895_s_at	**1,61**	0,001736	**1,62**	0,0009367	FKBP4	FK506 binding protein 4, 59kDa
205245_at	**1,61**	0,0002503	**1,43**	0,0015565	PARD6A	par-6 partitioning defective 6 homolog a
201124_at	**1,60**	0,0009086	**1,46**	0,0019597	ITGB5	integrin, beta 5
206781_at	**1,60**	0,0013948	**1,47**	0,002455	DNAJC4	DnaJ (Hsp40) homolog, subfamily C, m
208104_s_at	**1,60**	0,0019761	**1,53**	0,0010521	THG-1	TSC-22-like /// TSC-22-like
214105_at	**1,60**	0,0005779	**1,64**	7,82E-05		
213731_s_at	**1,59**	0,0001993	**1,45**	0,000535		
89476_r_at	**1,58**	0,0010408	**1,62**	0,0002035	NPEPL1	aminopeptidase-like 1
217046_s_at	**1,57**	0,0003684	**1,43**	0,0017643	AGER	advanced glycosylation end product-sp
204144_s_at	**1,56**	0,0012864	**1,47**	0,0022785	PIGQ	phosphatidylinositol glycan, class Q
218707_at	**1,55**	0,0007241	**1,60**	9,36E-05	ZNF444	zinc finger protein 444
221557_s_at	**1,55**	0,0004885	**1,52**	0,0001506	LEF1	lymphoid enhancer-binding factor 1
220341_s_at	**1,54**	6,64E-05	**1,56**	4,10E-06	LOC51149	truncated calcium binding protein
205561_at	**1,53**	0,0004947	**1,44**	0,0005562	FLJ12242	hypothetical protein FLJ12242
209665_at	**1,53**	0,0013421	**1,42**	0,0018571	CYB561D2	cytochrome b-561 domain containing 2
217794_at	**1,52**	2,00E-07	**1,28**	0,000104	DKFZP564J15	DKFZp564J157 protein
205906_at	**1,52**	0,0007773	**1,45**	0,000729	FOXJ1	forkhead box J1
207353_s_at	**1,51**	0,0018762	**1,42**	0,0015018	HMX1	homeo box (H6 family) 1
213512_at	**1,51**	0,00047	**1,61**	9,70E-06	C14orf79	chromosome 14 open reading frame 79
214125_s_at	**1,51**	0,0010882	**1,39**	0,0024183		
202045_s_at	**1,51**	1,00E-07	**1,25**	0,0008971	GRLF1	glucocorticoid receptor DNA binding fac
205806_at	**1,50**	0,000761	**1,60**	2,10E-05	ROM1	retinal outer segment membrane protei
214002_at	**1,50**	9,00E-07	**1,56**	4,00E-07	MYL6	myosin, light polypeptide 6, alkali, smoc
217079_at	**1,49**	0,0019614	**1,49**	0,0003226		
214386_at	**1,49**	0,0006884	**1,50**	8,76E-05		
219057_at	**1,47**	0,0010077	**1,36**	0,0012537	RABEP2	rabaptin, RAB GTPase binding effector
215082_at	**1,47**	0,0010575	**1,38**	0,0010943	ELOVL5	ELOVL family member 5, elongation of
208176_at	**1,46**	0,001821	**1,44**	0,0007611	DUX1	double homeobox, 1
222228_s_at	**1,46**	0,0007969	**1,46**	1,07E-05	FLJ20013	hypothetical protein FLJ20013
219417_s_at	**1,44**	6,15E-05	**1,39**	5,10E-06	FLJ20014	hypothetical protein FLJ20014
220193_at	**1,44**	0,0011884	**1,37**	0,0009533	FLJ22938	hypothetical protein FLJ22938
203996_s_at	**1,44**	0,0002625	**1,35**	0,0006825	C21orf2	chromosome 21 open reading frame 2
210161_at	**1,43**	0,0008939	**1,46**	0,000276		
204746_s_at	**1,43**	0,0006258	**1,48**	2,06E-05	PRKCABP	protein kinase C, alpha binding protein
204954_s_at	**1,43**	0,0006438	**1,56**	9,70E-06	DYRK1B	dual-specificity tyrosine-(Y)-phosphoryla
204648_at	**1,42**	0,0003444	**1,34**	0,0004544	NPR1	natriuretic peptide receptor A/guanylate
217054_at	**1,42**	0,0012435	**1,35**	0,0024817		CDNA FLJ39484 fis, clone PROST2014

220061_at	**1,42**	0,0003323	**1,30**	0,0011668	FLJ20581	hypothetical protein FLJ20581
220430_at	**1,40**	0,0018631	**1,42**	0,0001148	FLJ14050	hypothetical protein FLJ14050
209322_s_at	**1,39**	0,0021262	**1,35**	0,0011566	SH2B	SH2-B homolog
204660_at	**1,38**	0,0008522	**1,37**	0,0002827	GFER	growth factor, augmenter of liver regene
203682_s_at	**1,38**	0,0020853	**1,55**	0,0001552	IVD	isovaleryl Coenzyme A dehydrogenase
207004_at	**1,37**	0,0019432	**1,47**	0,0002903	BCL2	B-cell CLL/lymphoma 2
221232_s_at	**1,37**	0,0005102	**1,27**	0,001689	ANKRD2	ankyrin repeat domain 2 (stretch respor
204889_s_at	**1,36**	0,0002208	**1,36**	4,90E-05	NEURL	neuralized-like (Drosophila)
219766_at	**1,36**	0,0011097	**1,32**	0,0008085	MGC4093	hypothetical protein MGC4093
203586_s_at	**1,36**	0,0016477	**1,32**	0,0003952	ARF4L	ADP-ribosylation factor 4-like
212047_s_at	**1,35**	0,0018638	**1,31**	0,0007459	RNF167	ring finger protein 167
213433_at	**1,35**	0,0017691	**1,33**	0,0009181	ARL3	ADP-ribosylation factor-like 3
203273_s_at	**1,32**	0,0008243	**1,22**	0,0021979	TUSC2	tumor suppressor candidate 2
219986_s_at	**1,32**	0,000194	**1,35**	1,60E-06	ACAD10	acyl-Coenzyme A dehydrogenase famil
206739_at	**1,26**	2,24E-05	**1,26**	6,30E-06	HOXC5	homeo box C5
218257_s_at	**0,74**	0,0005943	**0,62**	2,00E-07	UGCGL1	UDP-glucose ceramide glucosyltransfe
212752_at	**0,67**	5,41E-05	**0,71**	6,65E-05	CLASP1	cytoplasmic linker associated protein 1
218420_s_at	**0,67**	0,0009414	**0,69**	5,86E-05	C13orf23	chromosome 13 open reading frame 23
203843_at	**0,66**	0,0020658	**0,71**	0,0022472	RPS6KA3	ribosomal protein S6 kinase, 90kDa, pc
221985_at	**0,63**	0,0002155	**0,73**	0,0012748	DRE1	DRE1 protein
203746_s_at	**0,63**	7,96E-05	**0,68**	0,0003097	HCCS	holocytochrome c synthase (cytochrom
203622_s_at	**0,63**	0,0008113	**0,65**	0,0003767	LOC56902	putatative 28 kDa protein
220668_s_at	**0,62**	9,00E-06	**0,61**	1,83E-05	DNMT3B	DNA (cytosine-5-)-methyltransferase 3
220941_s_at	**0,61**	0,0001908	**0,71**	0,0012136	C21orf91	chromosome 21 open reading frame 91
202754_at	**0,61**	6,57E-05	**0,65**	1,35E-05	R3HDM	R3H domain (binds single-stranded nuc
219862_s_at	**0,60**	4,94E-05	**0,55**	3,88E-05	NARF	nuclear prelamin A recognition factor
208433_s_at	**0,60**	0,0003524	**0,67**	0,0002879	LRP8	low density lipoprotein receptor-related
203702_s_at	**0,60**	0,0014806	**0,64**	0,0009352	TTLL4	tubulin tyrosine ligase-like family, memb
201393_s_at	**0,59**	0,002052	**0,47**	1,11E-05	IGF2R	insulin-like growth factor 2 receptor
210466_s_at	**0,58**	0,0004903	**0,71**	0,0010032	PAI-RBP1	PAI-1 mRNA-binding protein
219498_s_at	**0,58**	6,00E-06	**0,75**	0,0004189	BCL11A	B-cell CLL/lymphoma 11A (zinc finger p
200693_at	**0,57**	6,70E-05	**0,60**	8,25E-05	YWHAQ	tyrosine 3-monooxygenase/tryptophan
212350_at	**0,57**	8,10E-06	**0,69**	5,88E-05	TBC1D1	TBC1 (tre-2/USP6, BUB2, cdc16) doma
202370_s_at	**0,57**	0,0014139	**0,63**	0,0012305	CBFB	core-binding factor, beta subunit
204094_s_at	**0,56**	0,0021206	**0,57**	0,0002786	KIAA0669	KIAA0669 gene product
202511_s_at	**0,55**	0,0010962	**0,56**	0,0011034	APG5L	APG5 autophagy 5-like (S. cerevisiae)
218566_s_at	**0,55**	0,0005554	**0,58**	0,0001403	CHORDC1	cysteine and histidine-rich domain (CH(
217832_at	**0,54**	0,001183	**0,62**	0,0014086	SYNCRIP	synaptotagmin binding, cytoplasmic RN
212262_at	**0,54**	0,0012194	**0,59**	0,000456	QKI	quaking homolog, KH domain RNA binc
203139_at	**0,54**	8,20E-06	**0,50**	2,89E-05	DAPK1	death-associated protein kinase 1
212780_at	**0,54**	0,0009552	**0,67**	0,0001938	SOS1	son of sevenless homolog 1 (Drosophil
208398_s_at	**0,54**	0,0001142	**0,55**	0,000116	TBPL1	TBP-like 1
202188_at	**0,54**	1,45E-05	**0,68**	0,0006159	NUP93	nucleoporin 93kDa
202200_s_at	**0,54**	0,0005361	**0,55**	0,0002564	SRPK1	SFRS protein kinase 1
219675_s_at	**0,53**	0,0001389	**0,67**	0,0011532	UXS1	UDP-glucuronate decarboxylase 1
211764_s_at	**0,53**	8,53E-05	**0,59**	0,0002716	UBE2D1	ubiquitin-conjugating enzyme E2D 1 (U
218497_s_at	**0,53**	0,0003496	**0,67**	0,0001946	RNASEH1	ribonuclease H1
219960_s_at	**0,52**	0,0004888	**0,63**	0,000463	UCHL5	ubiquitin carboxyl-terminal hydrolase L!
204915_s_at	**0,52**	0,0015318	**0,16**	p < 1e-07	SOX11	SRY (sex determining region Y)-box 11
202147_s_at	**0,52**	0,0017243	**0,59**	0,0004569	IFRD1	interferon-related developmental regula
218092_s_at	**0,52**	0,0004283	**0,49**	8,30E-05	HRB	HIV-1 Rev binding protein
218826_at	**0,52**	2,49E-05	**0,57**	0,000117	SLC35F2	solute carrier family 35, member F2
221843_s_at	**0,51**	4,02E-05	**0,66**	0,0001168	KIAA1609	KIAA1609 protein
221676_s_at	**0,51**	0,0006185	**0,54**	0,0003082	CORO1C	coronin, actin binding protein, 1C
200821_at	**0,51**	0,0002944	**0,59**	0,0019771	LAMP2	lysosomal-associated membrane protei
212020_s_at	**0,51**	7,80E-06	**0,67**	0,0005729	MKI67	antigen identified by monoclonal antibo
202720_at	**0,51**	0,0004904	**0,55**	0,0003327	TES	testis derived transcript (3 LIM domains
218137_s_at	**0,50**	2,51E-05	**0,55**	3,56E-05	SMAP1	stromal membrane-associated protein [illegible]
212263_at	**0,50**	7,78E-05	**0,57**	8,65E-05	QKI	quaking homolog, KH domain RNA binc
213102_at	**0,50**	0,0001722	**0,58**	0,0004905	ACTR3	ARP3 actin-related protein 3 homolog (
205339_at	**0,49**	3,50E-06	**0,73**	0,0006869	SIL	TAL1 (SCL) interrupting locus
203837_at	**0,49**	0,0004758	**0,50**	0,0001882	MAP3K5	mitogen-activated protein kinase kinase
212022_s_at	**0,49**	7,87E-05	**0,61**	0,0008591	MKI67	antigen identified by monoclonal antibo
203222_s_at	**0,48**	0,0014137	**0,52**	0,0002874	TLE1	transducin-like enhancer of split 1 (E(sp
201833_at	**0,48**	0,0002381	**0,47**	6,95E-05	HDAC2	histone deacetylase 2
204198_s_at	**0,47**	0,0020493	**0,50**	0,0014011	RUNX3	runt-related transcription factor 3
205596_s_at	**0,47**	0,0001256	**0,55**	0,0007123	SMURF2	SMAD specific E3 ubiquitin protein liga:
203315_at	**0,47**	1,10E-06	**0,62**	0,0001114	NCK2	NCK adaptor protein 2
218486_at	**0,47**	3,31E-05	**0,67**	0,0025557	TIEG2	TGFB inducible early growth response
218726_at	**0,46**	1,90E-06	**0,69**	0,00155	DKFZp762E13	hypothetical protein DKFZp762E1312

220892_s_at	**0,46**	0,0004598	**0,50**	4,24E-05	PSAT1	phosphoserine aminotransferase 1
201197_at	**0,45**	0,0021375	**0,50**	0,000738	AMD1	adenosylmethionine decarboxylase 1
216640_s_at	**0,45**	6,38E-05	**0,66**	0,0005833	TXNDC7	thioredoxin domain containing 7 (protei
203418_at	**0,45**	0,0004249	**0,52**	0,0005556	CCNA2	cyclin A2
222201_s_at	**0,45**	2,11E-05	**0,61**	0,0014686	CASP8AP2	CASP8 associated protein 2
219588_s_at	**0,45**	0,0001128	**0,66**	0,0012417	MTB	more than blood homolog
221677_s_at	**0,44**	0,000673	**0,48**	8,07E-05		
205891_at	**0,43**	3,10E-06	**0,67**	0,000115	ADORA2B	adenosine A2b receptor
220094_s_at	**0,42**	2,90E-05	**0,63**	0,0012499	C6orf79	chromosome 6 open reading frame 79
211967_at	**0,42**	0,0001122	**0,48**	9,06E-05	PORIMIN	pro-oncosis receptor inducing membrar
205668_at	**0,41**	9,59E-05	**0,47**	0,0004218	LY75	lymphocyte antigen 75
218319_at	**0,40**	5,75E-05	**0,58**	0,0020946	PELI1	pellino homolog 1 (Drosophila)
209205_s_at	**0,40**	0,000256	**0,42**	0,000152	LMO4	LIM domain only 4
203405_at	**0,40**	1,96E-05	**0,60**	0,0021432	DSCR2	Down syndrome critical region gene 2
217834_s_at	**0,40**	1,96E-05	**0,61**	0,0018575	SYNCRIP	synaptotagmin binding, cytoplasmic RN
220651_s_at	**0,40**	2,68E-05	**0,49**	0,0012885	MCM10	MCM10 minichromosome maintenance
200602_at	**0,40**	9,10E-05	**0,55**	0,0020847	APP	amyloid beta (A4) precursor protein (pr
201589_at	**0,39**	1,31E-05	**0,56**	0,0010059	SMC1L1	SMC1 structural maintenance of chrom
214953_s_at	**0,39**	8,17E-05	**0,49**	0,0010164	APP	amyloid beta (A4) precursor protein (pr
209773_s_at	**0,38**	0,0001084	**0,35**	2,08E-05	RRM2	ribonucleotide reductase M2 polypeptid
210024_s_at	**0,38**	0,0001766	**0,44**	0,0004001	UBE2E3	ubiquitin-conjugating enzyme E2E 3 (U
200790_at	**0,37**	7,50E-05	**0,47**	0,0002728	ODC1	ornithine decarboxylase 1
204602_at	**0,36**	0,0003786	**0,24**	0,0003412	DKK1	dickkopf homolog 1 (Xenopus laevis)
201976_s_at	**0,36**	0,0001534	**0,47**	0,0012703	MYO10	myosin X
203510_at	**0,33**	0,0002367	**0,36**	2,56E-05	MET	met proto-oncogene (hepatocyte growtl
201088_at	**0,33**	0,0002656	**0,45**	0,0025671	KPNA2	karyopherin alpha 2 (RAG cohort 1, imp
201890_at	**0,33**	0,0008105	**0,37**	0,0005339	RRM2	ribonucleotide reductase M2 polypeptid
203574_at	**0,33**	7,40E-06	**0,54**	0,0017526		
219918_s_at	**0,33**	0,000238	**0,49**	0,0024498	ASPM	asp (abnormal spindle)-like, microcepha
212276_at	**0,30**	6,00E-07	**0,55**	3,00E-07	LPIN1	lipin 1
209288_s_at	**0,30**	8,77E-05	**0,50**	0,002508	CDC42EP3	CDC42 effector protein (Rho GTPase b
205681_at	**0,30**	0,0005541	**0,53**	0,0025246	BCL2A1	BCL2-related protein A1
212274_at	**0,30**	1,30E-06	**0,59**	4,12E-05	LPIN1	lipin 1
201579_at	**0,30**	1,49E-05	**0,53**	0,0023277	FAT	FAT tumor suppressor homolog 1 (Dros
219497_s_at	**0,29**	p < 1e-07	**0,63**	0,0006702	BCL11A	B-cell CLL/lymphoma 11A (zinc finger p
202270_at	**0,29**	4,96E-05	**0,43**	0,0014354	GBP1	guanylate binding protein 1, interferon-i
209642_at	**0,27**	2,00E-07	**0,56**	0,0002211	BUB1	BUB1 budding uninhibited by benzimida
204962_s_at	**0,25**	3,80E-06	**0,52**	0,0021298	CENPA	centromere protein A, 17kDa
204822_at	**0,19**	p < 1e-07	**0,43**	4,48E-05		
204304_s_at	**0,16**	6,60E-05	**0,20**	1,12E-05	PROM1	prominin 1
205347_s_at	**0,14**	1,19E-05	**0,27**	5,75E-05	TMSNB	thymosin, beta, identified in neuroblasto
215729_s_at	**0,08**	2,00E-07	**0,39**	0,0022683	VGLL1	vestigial like 1 (Drosophila)
