# Supplementary material for: Exquisite Sensitivity of TP53 Mutant and Basal Breast Cancers to a Dose-Dense Epirubicin−Cyclophosphamide Regimen
Source: PLoS Med. 2007 Mar 20;4(3):e90. doi: 10.1371/journal.pmed.0040090 (PMC1831731; doi:10.1371/journal.pmed.0040090)
Supplement: Table S4 — For details, see legend of Table S3. (86 KB PDF) [file pmed.0040090.st004.pdf]

Probe set	C2/C1	C1vC2 p-value	C2/C3	C2vC3 p-value	Gene Symbol	Description
205044_at	15,19	4,00E-07	20,75	3,82E-05	GABRP	gamma-aminobutyric acid (GABA) A re
220559_at	10,71	p < 1e-07	8,58	2,43E-04	EN1	engrailed homolog 1
202037_s_at	8,88	1,00E-05	9,19	1,40E-04	SFRP1	secreted frizzled-related protein 1
218963_s_at	8,70	2,10E-06	4,42	0,02	KRT23	keratin 23 (histone deacetylase inducib
204855_at	7,56	5,00E-07	9,08	3,76E-05	SERPINB5	serine (or cysteine) proteinase inhibitor,
202036_s_at	6,77	1,04E-05	9,27	1,77E-04	SFRP1	secreted frizzled-related protein 1
209800_at	6,73	p < 1e-07	6,33	3,10E-06	KRT16	keratin 16 (focal non-epidermolytic paln
213680_at	6,73	6,14E-05	6,37	5,70E-03	KRT6B	keratin 6B
220625_s_at	6,66	1,22E-05	4,20	0,01	ELF5	E74-like factor 5 (ets domain transcripti
207039_at	6,57	p < 1e-07	5,56	6,41E-04	CDKN2A	cyclin-dependent kinase inhibitor 2A (m
206373_at	6,47	p < 1e-07	4,56	4,66E-03	ZIC1	
205475_at	6,39	p < 1e-07	6,15	1,14E-04	SCRG1	scrapie responsive protein 1
213711_at	6,18	4,00E-07	4,34	0,01	KRTHB1	keratin, hair, basic, 1
206632_s_at	6,16	7,00E-07	3,95	1,06E-04	APOBEC3B	apolipoprotein B mRNA editing enzyme
213492_at	6,08	7,89E-05	6,31	5,29E-03	COL2A1	collagen, type II, alpha 1 (primary ostec
206560_s_at	5,98	4,00E-07	9,42	2,43E-05	MIA	melanoma inhibitory activity
209283_at	5,95	3,90E-06	6,18	5,85E-04	CRYAB	crystallin, alpha B
204259_at	5,62	1,70E-06	4,63	8,76E-03	MMP7	
205157_s_at	5,61	5,19E-05	5,58	5,32E-03	KRT17	keratin 17
202342_s_at	5,44	7,00E-07	3,04	2,61E-03	TRIM2	tripartite motif-containing 2
212730_at	4,73	6,25E-05	5,58	2,70E-06	DMN	desmuslin
209289_at	4,68	6,83E-05	2,09	0,02	NFIB	nuclear factor I/B
202965_s_at	4,67	0,0010676	6,26	2,66E-03	CAPN6	calpain 6
36711_at	4,58	7,62E-05	3,42	5,69E-04	MAFF	v-maf musculoaponeurotic fibrosarcom
209842_at	4,57	1,20E-06	5,22	2,16E-04	SOX10	SRY (sex determining region Y)-box 10
202912_at	4,57	0,0001947	3,17	7,09E-03	ADM	adrenomedullin
204724_s_at	4,55	1,00E-07	4,85	6,14E-05	COL9A3	collagen, type IX, alpha 3
208103_s_at	4,46	1,00E-06	3,65	2,92E-03	ANP32E	acidic (leucine-rich) nuclear phosphopr
209290_s_at	4,43	7,67E-05	1,90	0,02	NFIB	nuclear factor I/B
213338_at	4,28	0,0001359	3,11	7,60E-03	RIS1	Ras-induced senescence 1
205350_at	4,10	0,0008554	4,60	6,50E-03	CRABP1	cellular retinoic acid binding protein 1
207030_s_at	4,09	0,0002163	2,78	0,03	CSRP2	cysteine and glycine-rich protein 2
209387_s_at	4,06	0,0001308	3,25	5,67E-04	TM4SF1	transmembrane 4 superfamily member
214297_at	3,98	p < 1e-07	4,39	2,90E-06		
215034_s_at	3,95	0,0001989	3,41	1,31E-04	TM4SF1	transmembrane 4 superfamily member
209170_s_at	3,95	0,0001436	3,61	8,51E-03	GPM6B	glycoprotein M6B
202870_s_at	3,92	5,39E-05	2,18	7,79E-03	CDC20	CDC20 cell division cycle 20 homolog (
218868_at	3,86	p < 1e-07	3,44	1,82E-04	ARP3BETA	actin-related protein 3-beta
201195_s_at	3,79	3,82E-05	2,13	0,02	SLC7A5	solute carrier family 7 (cationic amino a
209504_s_at	3,79	5,78E-05	3,90	1,19E-04	PLEKHB1	pleckstrin homology domain containing
201820_at	3,77	0,0005098	3,66	0,01	KRT5	keratin 5 (epidermolysis bullosa simple
203637_s_at	3,73	2,03E-05	2,57	0,01	MID1	midline 1 (Opitz/BBB syndrome)
202887_s_at	3,68	1,30E-06	2,40	4,85E-03	DDIT4	DNA-damage-inducible transcript 4
202504_at	3,66	6,40E-06	2,82	4,36E-03	TRIM29	tripartite motif-containing 29
214240_at	3,62	9,30E-06	2,91	7,38E-03	GAL	galanin
209386_at	3,62	0,000532	3,15	2,25E-04	TM4SF1	transmembrane 4 superfamily member
203256_at	3,61	2,14E-05	2,11	0,03	CDH3	cadherin 3, type 1, P-cadherin (placent
210512_s_at	3,58	2,03E-05	1,80	0,03	VEGF	vascular endothelial growth factor
203400_s_at	3,58	0,0002227	3,50	7,22E-03	TF	transferrin
218755_at	3,54	7,90E-06	2,73	1,28E-04	KIF20A	kinesin family member 20A
203126_at	3,49	2,18E-05	3,07	1,97E-03	IMPA2	inositol(myo)-1(or 4)-monophosphatase
204437_s_at	3,43	7,95E-05	3,01	8,16E-03	FOLR1	folate receptor 1 (adult)
204825_at	3,40	9,90E-06	2,20	3,41E-03	MELK	maternal embryonic leucine zipper kina
213032_at	3,33	0,0002554	2,13	0,04	NFIB	nuclear factor I/B
204733_at	3,30	6,99E-05	3,26	9,75E-03	KLK6	kallikrein 6 (neurosin, zyme)
217996_at	3,30	0,000134	2,35	0,03	PHLDA1	pleckstrin homology-like domain, family
213122_at	3,29	0,0001487	2,25	0,03	TSPYL5	TSPY-like 5
201397_at	3,26	2,40E-06	2,05	1,12E-03	PHGDH	phosphoglycerate dehydrogenase
202705_at	3,26	4,70E-06	1,86	0,02	CCNB2	cyclin B2
203924_at	3,25	8,38E-05	3,23	0,01	GSTA1	glutathione S-transferase A1
202589_at	3,25	2,07E-05	3,16	1,78E-04	TYMS	thymidylate synthetase
205569_at	3,25	0,0002846	1,90	0,03	LAMP3	lysosomal-associated membrane protei
212816_s_at	3,24	1,82E-05	2,60	6,55E-03	CBS	cystathionine-beta-synthase
213456_at	3,24	2,00E-07	3,71	1,73E-04	SOSTDC1	sclerostin domain containing 1
203764_at	3,24	0,0001493	1,76	0,02	DLG7	discs, large homolog 7 (Drosophila)
55081_at	3,24	4,00E-07	2,62	1,79E-04	MICAL-L1	MICAL-like 1
218847_at	3,22	p < 1e-07	3,00	7,87E-05	IMP-2	IGF-II mRNA-binding protein 2
203755_at	3,21	0,0002697	2,13	0,02	BUB1B	BUB1 budding uninhibited by benzimid

205334_at	3,19	0,0009255	3,60	5,50E-03	S100A1	S100 calcium binding protein A1
204162_at	3,14	0,0015791	3,19	0,01	KNTC2	kinetochore associated 2
209167_at	3,14	0,0002638	2,71	0,02	GPM6B	glycoprotein M6B
210052_s_at	3,13	1,25E-05	1,72	5,92E-03	TPX2	TPX2, microtubule-associated protein h
200632_s_at	3,13	7,73E-05	2,51	1,48E-03	NDRG1	N-myc downstream regulated gene 1
202946_s_at	3,06	0,0015678	2,66	0,01	BTBD3	BTB (POZ) domain containing 3
218009_s_at	3,04	0,0005979	2,58	7,18E-04	PRC1	protein regulator of cytokinesis 1
209074_s_at	3,00	2,20E-06	3,18	3,78E-04	TU3A	TU3A protein
204030_s_at	2,98	1,48E-05	3,69	1,44E-04	SCHIP1	schwannomin interacting protein 1
209409_at	2,96	0,0003511	2,47	2,81E-03	GRB10	growth factor receptor-bound protein 10
221505_at	2,96	0,0001453	2,83	4,46E-03	ANP32E	acidic (leucine-rich) nuclear phosphopro
205778_at	2,96	0,0005714	2,83	0,03	KLK7	kallikrein 7 (chymotryptic, stratum corne
212771_at	2,94	2,70E-06	2,59	1,40E-03	C10orf38	chromosome 10 open reading frame 38
210347_s_at	2,94	p < 1e-07	2,39	1,82E-03	BCL11A	B-cell CLL/lymphoma 11A (zinc finger p
214051_at	2,93	8,30E-06	2,13	0,03	MGC39900	hypothetical protein MGC39900
222062_at	2,90	0,0004328	2,62	0,02	IL27RA	interleukin 27 receptor, alpha
202341_s_at	2,89	3,31E-05	2,41	4,18E-03	TRIM2	tripartite motif-containing 2
213060_s_at	2,88	0,0005108	3,11	8,98E-03	CHI3L2	chitinase 3-like 2
200934_at	2,88	2,87E-05	2,60	8,49E-04	DEK	DEK oncogene (DNA binding)
203693_s_at	2,87	6,67E-05	2,39	3,33E-03	E2F3	E2F transcription factor 3
203358_s_at	2,86	6,96E-05	1,88	0,03	EZH2	enhancer of zeste homolog 2 (Drosoph
219582_at	2,83	3,50E-06	2,35	1,11E-03	OGFRL1	opioid growth factor receptor-like 1
201037_at	2,83	0,0004922	2,66	1,04E-03	PFKP	phosphofructokinase, platelet
201656_at	2,83	9,04E-05	1,89	4,17E-03	ITGA6	integrin, alpha 6
208165_s_at	2,82	5,61E-05	2,97	4,82E-03	PRSS16	protease, serine, 16 (thymus)
204641_at	2,82	0,0002196	1,91	0,01	NEK2	NIMA (never in mitosis gene a)-related
221510_s_at	2,81	8,00E-07	2,11	1,25E-03	GLS	glutaminase
201795_at	2,80	0,0001132	1,91	0,02	LBR	lamin B receptor
221016_s_at	2,79	2,00E-07	2,05	4,53E-04	TCF7L1	transcription factor 7-like 1 (T-cell speci
209834_at	2,79	1,00E-07	2,44	2,95E-04	CHST3	carbohydrate (chondroitin 6) sulfotransf
205199_at	2,79	4,15E-05	2,17	0,01	CA9	carbonic anhydrase IX
216237_s_at	2,77	2,20E-06	2,39	1,34E-04	MCM5	MCM5 minichromosome maintenance c
218984_at	2,74	9,26E-05	2,18	7,73E-03	FLJ20485	hypothetical protein FLJ20485
204170_s_at	2,73	0,0002492	1,84	0,01	CKS2	CDC28 protein kinase regulatory subur
211953_s_at	2,70	5,92E-05	2,28	1,00E-03	RANBP5	RAN binding protein 5
203636_at	2,70	0,0004695	2,28	0,02	MID1	midline 1 (Opitz/BBB syndrome)
204146_at	2,70	0,0007673	2,70	3,50E-03	RAD51AP1	RAD51 associated protein 1
219654_at	2,69	0,0001385	2,67	7,68E-03	PTPLA	protein tyrosine phosphatase-like (prolin
204060_s_at	2,68	3,00E-07	2,05	1,81E-03	PRKX /// PRKY	protein kinase, X-linked /// protein kinas
206055_s_at	2,68	0,0002348	2,08	1,60E-04	SNRPA1	small nuclear ribonucleoprotein polypep
33323_r_at	2,67	0,0013291	4,32	6,10E-05	SFN	stratifin
218424_s_at	2,65	6,00E-07	1,86	0,01	TSAP6	dudulin 2
221203_s_at	2,63	5,00E-07	2,01	2,42E-03	YEATS2	YEATS domain containing 2
202666_s_at	2,61	4,80E-06	2,01	2,12E-03	ACTL6A	actin-like 6A
33322_i_at	2,57	0,0005525	4,17	7,09E-05	SFN	stratifin
221523_s_at	2,50	0,0016893	2,38	4,67E-03	RRAGD	Ras-related GTP binding D
200783_s_at	2,50	1,32E-05	2,03	1,33E-03	STMN1	stathmin 1/oncoprotein 18
212265_at	2,49	6,78E-05	1,49	0,04	QKI	quaking homolog, KH domain RNA binc
202446_s_at	2,48	4,01E-05	2,60	1,50E-04	PLSCR1	phospholipid scramblase 1
213226_at	2,47	0,0001356	1,85	0,03	CCNA2	cyclin A2
213260_at	2,46	0,0001757	3,26	5,34E-04	FOXC1	forkhead box C1
205047_s_at	2,45	0,0001994	2,08	0,01	ASNS	asparagine synthetase
209191_at	2,45	0,0019629	2,65	1,33E-03	MGC4083	tubulin beta MGC4083
201250_s_at	2,45	3,10E-06	2,15	8,06E-04	SLC2A1	solute carrier family 2 (facilitated glucos
209815_at	2,43	0,0015745	2,44	7,62E-03	PTCH	Patched homolog (Drosophila)
200827_at	2,41	2,90E-06	2,14	2,98E-04	PLOD	procollagen-lysine, 2-oxoglutarate 5-dic
209523_at	2,37	0,0009531	2,18	6,36E-03	TAF2	TAF2 RNA polymerase II, TATA box bin
213346_at	2,37	4,43E-05	2,06	2,98E-03	LOC93081	hypothetical protein BC015148
205505_at	2,37	2,10E-06	2,18	3,07E-03	GCNT1	glucosaminyl (N-acetyl) transferase 1, c
212330_at	2,37	1,52E-05	2,46	5,97E-04	TFDP1	transcription factor Dp-1
210605_s_at	2,37	4,41E-05	2,56	1,82E-03	MFGE8	milk fat globule-EGF factor 8 protein
218252_at	2,37	2,83E-05	1,73	0,03	CKAP2	cytoskeleton associated protein 2
201755_at	2,36	7,70E-06	1,92	1,98E-03	MCM5	MCM5 minichromosome maintenance c
202097_at	2,36	0,0001827	1,79	3,41E-03	NUP153	nucleoporin 153kDa
208079_s_at	2,34	0,0008204	2,07	0,01	STK6	serine/threonine kinase 6
212572_at	2,34	2,11E-05	1,90	5,31E-03	STK38L	serine/threonine kinase 38 like
204061_at	2,34	8,00E-07	1,75	9,13E-03	PRKX	protein kinase, X-linked
203120_at	2,32	0,0002079	1,88	0,02	TP53BP2	tumor protein p53 binding protein, 2
213005_s_at	2,32	0,001727	2,56	3,56E-04	ANKRD15	ankyrin repeat domain 15
32094_at	2,32	3,10E-06	2,45	1,64E-04	CHST3	carbohydrate (chondroitin 6) sulfotransf

218440_at	2,31	5,90E-05	1,60	0,03	MCCC1	methylcrotonoyl-Coenzyme A carboxyla
221854_at	2,31	0,0007883	2,16	0,03	PKP1	plakophilin 1 (ectodermal dysplasia/skir
210115_at	2,31	0,0004422	2,13	7,51E-03	RPL39L	ribosomal protein L39-like
204023_at	2,29	0,0003031	2,10	3,01E-03	RFC4	
201664_at	2,29	0,0008016	1,94	0,02	SMC4L1	SMC4 structural maintenance of chrom
212846_at	2,28	0,0007989	1,88	0,01	KIAA0179	KIAA0179
215945_s_at	2,28	8,41E-05	2,04	0,01	TRIM2	tripartite motif-containing 2
213154_s_at	2,27	0,0005874	1,60	0,04	BICD2	bicaudal D homolog 2 (Drosophila)
221185_s_at	2,26	0,0006852	2,28	0,01	DKFZp434B22	hypothetical protein DKFZp434B227
209270_at	2,26	0,0001226	2,96	2,34E-05	LAMB3	laminin, beta 3
208078_s_at	2,26	0,0002358	1,82	0,03	SNF1LK	SNF1-like kinase /// SNF1-like kinase
221591_s_at	2,26	3,30E-06	1,89	1,08E-03	FLJ10156	hypothetical protein FLJ10156
209260_at	2,26	0,0002122	2,52	4,32E-03	SFN	stratifin
204767_s_at	2,25	0,0001844	2,03	1,88E-03	FEN1	flap structure-specific endonuclease 1
204401_at	2,24	6,10E-05	2,00	5,10E-03	KCNN4	potassium intermediate/small conducta
222039_at	2,23	2,58E-05	2,31	2,22E-04	LOC146909	hypothetical protein LOC146909
221779_at	2,23	p < 1e-07	2,03	1,07E-04	MICAL-L1	MICAL-like 1
213484_at	2,23	0,0003298	2,23	0,01		Clone 23700 mRNA sequence
202185_at	2,22	p < 1e-07	2,25	2,38E-05	PLOD3	procollagen-lysine, 2-oxoglutarate 5-dic
210999_s_at	2,22	0,0001959	1,99	0,01	GRB10	growth factor receptor-bound protein 1(
213310_at	2,21	0,0016839	2,20	0,01	EIF2C2	eukaryotic translation initiation factor 2(
209464_at	2,21	0,0001494	2,20	1,61E-04	AURKB	aurora kinase B
218877_s_at	2,19	0,0004537	2,24	2,24E-03	C6orf75	chromosome 6 open reading frame 75
210074_at	2,19	8,36E-05	1,72	0,03	CTSL2	cathepsin L2
219825_at	2,19	0,0007698	1,99	0,02	CYP26B1	cytochrome P450, family 26, subfamily
204318_s_at	2,18	0,0012859	1,44	0,04	GTSE1	G-2 and S-phase expressed 1
204092_s_at	2,18	0,0001039	1,52	0,02	STK6	serine/threonine kinase 6
218898_at	2,17	3,74E-05	2,22	2,11E-05	CT120	membrane protein expressed in epithel
219060_at	2,15	9,51E-05	1,97	0,01	FLJ10204	hypothetical protein FLJ10204
211964_at	2,14	0,0001271	1,93	3,44E-04	COL4A2	collagen, type IV, alpha 2
203939_at	2,14	0,0017725	2,14	5,94E-03	NT5E	5'-nucleotidase, ecto (CD73)
219225_at	2,13	2,76E-05	1,75	0,03	PGBD5	piggyBac transposable element derivec
213113_s_at	2,13	6,00E-07	1,80	3,16E-03	SLC43A3	solute carrier family 43, member 3
209212_s_at	2,13	1,00E-06	2,07	8,87E-05	KLF5	Kruppel-like factor 5 (intestinal)
201896_s_at	2,12	0,0006071	1,79	6,82E-03	DDA3	differential display and activated by p53
203074_at	2,12	6,58E-05	2,15	0,01	ANXA8	annexin A8
221520_s_at	2,11	2,64E-05	1,53	0,02	CDCA8	cell division cycle associated 8
213404_s_at	2,10	0,0005554	1,55	0,01	RHEB	Ras homolog enriched in brain
204203_at	2,10	0,0005496	1,92	5,13E-03	CEBPG	CCAAT/enhancer binding protein (C/EE
202625_at	2,09	6,71E-05	1,50	0,04		v-yes-1 Yamaguchi sarcoma viral relate
219735_s_at	2,09	2,00E-07	2,12	3,52E-05	TFCP2L1	transcription factor CP2-like 1
209015_s_at	2,09	5,38E-05	1,97	2,55E-03	DNAJB6	DnaJ (Hsp40) homolog, subfamily B, m
209408_at	2,08	2,64E-05	1,51	2,91E-03	KIF2C	kinesin family member 2C
219006_at	2,08	0,0002082	1,52	0,02	C6orf66	chromosome 6 open reading frame 66
204159_at	2,07	0,0006958	2,24	1,24E-04	CDKN2C	cyclin-dependent kinase inhibitor 2C (p
208092_s_at	2,07	3,00E-07	1,68	0,01	FAM49A	family with sequence similarity 49, mem
210093_s_at	2,06	8,06E-05	1,85	0,01	MAGOH	mago-nashi homolog, proliferation-assc
202430_s_at	2,06	3,25E-05	1,84	2,23E-03	PLSCR1	phospholipid scramblase 1
204730_at	2,06	8,80E-06	1,90	3,32E-03	RIMS3	regulating synaptic membrane exocytos
202267_at	2,05	0,001087	2,56	1,76E-03	LAMC2	
205240_at	2,04	0,0002045	1,67	0,02	GPSM2	G-protein signalling modulator 2 (AGS3
214684_at	2,04	3,37E-05	1,55	0,01	MEF2A	MADS box transcription enhancer facto
204126_s_at	2,03	0,0002612	1,70	6,53E-03	CDC45L	CDC45 cell division cycle 45-like (S. ce
210845_s_at	2,03	0,0006504	1,57	0,02	PLAUR	plasminogen activator, urokinase recep
219306_at	2,03	0,0009928	1,87	0,03	KNSL7	kinesin-like 7
209122_at	2,03	0,0009397	1,97	1,98E-03	ADFP	adipose differentiation-related protein
212023_s_at	2,03	1,97E-05	1,69	8,00E-03	MKI67	antigen identified by monoclonal antibo
203687_at	2,02	3,89E-05	1,58	0,03	CX3CL1	chemokine (C-X3-C motif) ligand 1
200052_s_at	2,01	0,000188	1,47	2,05E-03	ILF2	interleukin enhancer binding factor 2, 4
203211_s_at	2,00	0,0012367	1,55	0,04	MTMR2	myotubularin related protein 2
221909_at	2,00	0,000245	1,98	0,02	FLJ14627	hypothetical protein FLJ14627
209609_s_at	2,00	0,0003222	1,67	1,20E-03	MRPL9	mitochondrial ribosomal protein L9
208795_s_at	2,00	0,0005477	2,10	1,62E-03	MCM7	MCM7 minichromosome maintenance (
221524_s_at	2,00	0,0013384	1,72	0,01	RRAGD	Ras-related GTP binding D
202468_s_at	1,99	0,0006739	1,62	0,04	CTNNAL1	catenin (cadherin-associated protein), a
201201_at	1,99	8,40E-06	1,58	3,64E-03	CSTB	cystatin B (stefin B)
201528_at	1,99	0,0014703	1,58	0,02	RPA1	replication protein A1, 70kDa
203234_at	1,97	0,0001787	2,16	1,52E-03	UPP1	uridine phosphorylase 1
209871_s_at	1,97	0,0001755	1,50	0,03	APBA2	amyloid beta (A4) precursor protein-bin
212074_at	1,97	0,0009592	1,51	0,02	UNC84A	unc-84 homolog A (C. elegans)

201564_s_at	1,95	0,0003645	1,96	1,41E-03	FSCN1	fascin homolog 1, actin-bundling protei
205098_at	1,95	0,000242	1,44	0,04	CCR1	
220643_s_at	1,95	0,0008989	1,73	0,04	FAIM	Fas apoptotic inhibitory molecule
202245_at	1,95	0,0004378	1,64	4,54E-03	LSS	lanosterol synthase (2,3-oxidosqualene
201830_s_at	1,95	0,0007305	2,33	9,03E-05	NET1	neuroepithelial cell transforming gene 1
212378_at	1,94	5,29E-05	1,76	5,15E-04	GART	phosphoribosylglycinamide formyltrans
203909_at	1,91	0,0007544	1,58	0,03	SLC9A6	solute carrier family 9 (sodium/hydroge
203805_s_at	1,91	7,63E-05	1,78	3,84E-03	FANCA	Fanconi anemia, complementation grou
221879_at	1,91	0,0020062	2,10	4,66E-03	CALML4	calmodulin-like 4
205348_s_at	1,91	2,64E-05	1,98	2,84E-03	DNCI1	dynein, cytoplasmic, intermediate polyp
201275_at	1,90	7,28E-05	1,63	7,91E-03	FDPS	farnesyl diphosphate synthase (farnesy
218308_at	1,90	0,0001618	1,55	0,02	TACC3	transforming, acidic coiled-coil containi
212973_at	1,90	0,0002945	1,87	1,91E-03	RPIA	ribose 5-phosphate isomerase A (ribose
205356_at	1,89	0,0002186	1,97	3,39E-03	USP13	ubiquitin specific protease 13 (isopeptic
218018_at	1,89	0,0004504	1,72	0,02	PDXK	pyridoxal (pyridoxine, vitamin B6) kinas
213707_s_at	1,89	1,54E-05	1,82	1,52E-03	DLX5	distal-less homeo box 5
221436_s_at	1,89	0,0005092	1,79	1,55E-04	CDCA3	cell division cycle associated 3 /// cell d
202715_at	1,88	0,0004083	1,51	0,01	CAD	carbamoyl-phosphate synthetase 2, as
209511_at	1,87	0,0001822	2,07	1,67E-04	POLR2F	polymerase (RNA) II (DNA directed) po
220233_at	1,86	0,0002056	2,15	3,41E-04	FBXO17	F-box protein 17
217957_at	1,86	0,0011091	1,63	8,86E-03	GTL3	likely ortholog of mouse gene trap locus
205268_s_at	1,85	5,73E-05	1,85	3,77E-03	ADD2	adducin 2 (beta)
219403_s_at	1,85	0,0005874	1,49	0,02	HPSE	heparanase
204768_s_at	1,85	0,0015209	1,50	0,02	FEN1	flap structure-specific endonuclease 1
203390_s_at	1,83	0,0001032	1,51	0,01	KIF3C	kinesin family member 3C
201751_at	1,82	2,90E-05	1,68	1,52E-04	KIAA0063	KIAA0063 gene product
203692_s_at	1,82	2,14E-05	1,77	2,26E-03	E2F3	E2F transcription factor 3
208855_s_at	1,81	0,000154	2,14	1,00E-07	STK24	serine/threonine kinase 24 (STE20 hon
202858_at	1,81	0,0001086	1,38	0,02	U2AF1	U2(RNU2) small nuclear RNA auxiliary
216952_s_at	1,81	0,0001447	1,46	6,98E-03	LMNB2	lamin B2
202804_at	1,80	3,00E-07	1,44	8,95E-03	ABCC1	ATP-binding cassette, sub-family C (CF
201587_s_at	1,80	0,0018226	2,14	7,53E-04	IRAK1	interleukin-1 receptor-associated kinase
202854_at	1,79	0,0020414	2,02	1,85E-04	HPRT1	hypoxanthine phosphoribosyltransferas
202483_s_at	1,78	0,0006575	1,48	0,02	RANBP1	RAN binding protein 1
214838_at	1,78	3,52E-05	1,74	4,30E-06	LOC375035	hypothetical protein LOC375035
220011_at	1,77	0,0001222	1,41	1,46E-03	MGC2603	hypothetical protein MGC2603
204600_at	1,77	0,0002507	2,33	1,07E-05	EPHB3	EphB3
203298_s_at	1,76	0,001558	1,41	0,02	JARID2	Jumonji, AT rich interactive domain 2
218593_at	1,76	0,0006303	1,51	0,01	RBM28	RNA binding motif protein 28
207692_s_at	1,76	0,0006863	1,83	0,01	AGC1	aggrecan 1 (chondroitin sulfate proteog
211275_s_at	1,76	0,0002539	1,68	1,66E-03	GYG	glycogenin
205733_at	1,75	0,0002375	1,44	0,02	BLM	Bloom syndrome
213870_at	1,73	3,45E-05	1,88	4,82E-04		
212693_at	1,72	0,0016166	1,50	0,01	MDN1	MDN1, midasin homolog (yeast)
204558_at	1,72	0,0002214	1,77	3,28E-03	RAD54L	RAD54-like (S. cerevisiae)
203861_s_at	1,70	0,0015428	1,64	0,02	ACTN2	actinin, alpha 2
219509_at	1,70	9,75E-05	1,73	0,01	MYOZ1	myozenin 1
209313_at	1,69	0,0007572	1,56	0,03	XAB1	XPA binding protein 1
219336_s_at	1,69	0,0010334	1,63	0,01	ASCC1	activating signal cointegrator 1 complex
201819_at	1,68	0,0006578	2,35	3,02E-04	SCARB1	scavenger receptor class B, member 1
208854_s_at	1,68	0,0002326	1,94	3,46E-05	STK24	serine/threonine kinase 24 (STE20 hon
205595_at	1,67	1,40E-06	1,62	0,02	DSG3	desmoglein 3 (pemphigus vulgaris antig
201837_s_at	1,67	0,0004284	1,67	5,79E-03	STAF65(gamm	SPTF-associated factor 65 gamma
205743_at	1,67	4,10E-06	1,37	0,01	STAC	SH3 and cysteine rich domain
219698_s_at	1,66	0,0013482	1,61	0,02	METTL4	methyltransferase like 4
205080_at	1,66	0,001514	1,54	0,03	RARB	retinoic acid receptor, beta
215195_at	1,66	1,35E-05	1,55	1,63E-03	PRKCA	protein kinase C, alpha
205067_at	1,66	3,60E-06	1,50	5,54E-03	IL1B	interleukin 1, beta
222056_s_at	1,66	0,0013573	1,56	3,64E-03	FAHD2A	fumarylacetoacetate hydrolase domain
202134_s_at	1,65	0,0001678	1,53	0,01	TAZ	transcriptional co-activator with PDZ-bi
209715_at	1,65	0,0004136	1,67	0,01	CBX5	chromobox homolog 5 (HP1 alpha hom
218104_at	1,65	0,0004575	1,41	0,02	TEX10	testis expressed sequence 10
204593_s_at	1,64	0,0004222	1,35	0,01	FLJ20232	hypothetical protein FLJ20232
221908_at	1,63	0,0009105	1,86	1,54E-03	FLJ14627	hypothetical protein FLJ14627
205284_at	1,63	0,0006055	1,73	1,75E-03	KIAA0133	KIAA0133 gene product
212399_s_at	1,62	0,0004123	1,57	0,01	VGLL4	vestigial like 4 (Drosophila)
209042_s_at	1,62	0,0014153	1,40	0,03	UBE2G2	ubiquitin-conjugating enzyme E2G 2 (U
204117_at	1,60	0,0001216	1,31	0,01	PREP	prolyl endopeptidase
200830_at	1,60	0,0010246	1,76	2,76E-03	PSMD2	proteasome (prosome, macropain) 26S
202548_s_at	1,59	0,0014737	1,78	8,79E-04	ARHGEF7	Rho guanine nucleotide exchange facto

208964_s_at	1,59	0,000428	1,33	0,04	FADS1	fatty acid desaturase 1
218902_at	1,59	0,0009952	1,84	1,75E-03	NOTCH1	Notch homolog 1, translocation-associa
205637_s_at	1,58	7,70E-06	1,37	0,01	SH3GL3	SH3-domain GRB2-like 3
215947_s_at	1,58	0,0004806	1,27	0,04	FLJ14668	hypothetical protein FLJ14668
211594_s_at	1,58	0,000121	1,51	1,20E-03	MRPL9	mitochondrial ribosomal protein L9 /// m
200913_at	1,57	2,34E-05	1,38	0,04	PPM1G	protein phosphatase 1G (formerly 2C),
37950_at	1,56	2,49E-05	1,36	6,93E-03	PREP	prolyl endopeptidase
200792_at	1,56	0,00115	1,62	2,32E-03	G22P1	thyroid autoantigen 70kDa (Ku antigen)
205524_s_at	1,56	0,000121	1,51	7,52E-03	HAPLN1	hyaluronan and proteoglycan link prote
209410_s_at	1,56	6,51E-05	1,34	0,02	GRB10	growth factor receptor-bound protein 10
201463_s_at	1,55	0,0016213	1,52	9,83E-04	TALDO1	transaldolase 1
207508_at	1,54	0,0002307	1,37	0,01	ATP5G3	ATP synthase, H+ transporting, mitoch
213497_at	1,53	0,0001568	1,50	2,30E-03	ABTB2	ankyrin repeat and BTB (POZ) domain
204447_at	1,53	0,000196	1,36	0,04	ProSAPiP1	ProSAPiP1 protein
200957_s_at	1,52	0,0001042	1,49	0,02	SSRP1	structure specific recognition protein 1
204695_at	1,52	0,0007839	1,71	0,01	CDC25A	cell division cycle 25A
212144_at	1,51	0,0011655	1,67	3,05E-04	UNC84B	unc-84 homolog B (C. elegans)
205193_at	1,51	0,0004847	1,43	0,02	MAFF	v-maf musculoaponeurotic fibrosarcoma
214253_s_at	1,51	0,0003472	1,37	0,04	DTNB	dystrobrevin, beta
205008_s_at	1,50	5,72E-05	1,50	1,82E-03	CIB2	calcium and integrin binding family mer
219270_at	1,50	0,0004525	1,33	2,02E-03	MGC4504	hypothetical protein MGC4504
202244_at	1,48	0,0002526	1,79	3,58E-05	PSMB4	proteasome (prosome, macropain) sub
200677_at	1,48	2,16E-05	1,32	0,03	PTTG1IP	pituitary tumor-transforming 1 interactin
64474_g_at	1,47	3,34E-05	1,40	4,00E-03	DGCR8	DiGeorge syndrome critical region gene
209054_s_at	1,47	0,0016388	1,61	9,01E-03	WHSC1	Wolf-Hirschhorn syndrome candidate 1
210357_s_at	1,47	0,0001151	1,45	3,15E-04	SMOX	spermine oxidase
201306_s_at	1,45	0,0013659	1,38	4,60E-03	ANP32B	acidic (leucine-rich) nuclear phosphopr
204441_s_at	1,45	0,0004299	1,50	7,09E-04	POLA2	polymerase (DNA-directed), alpha (70k
203389_at	1,45	0,0004131	1,33	0,02	KIF3C	kinesin family member 3C
39402_at	1,45	4,59E-05	1,36	0,01	IL1B	interleukin 1, beta
219265_at	1,44	0,0005475	1,50	8,09E-03	MOBKL2B	MOB1, Mps One Binder kinase activato
203821_at	1,43	0,0003667	1,36	4,38E-03	DTR	diphtheria toxin receptor (heparin-bindi
219180_s_at	1,43	0,0012113	1,36	0,01	PEX26	peroxisome biogenesis factor 26
212528_at	1,43	0,0003912	1,33	5,74E-03	D15Wsu75e	Full-length cDNA clone CS0DI082YE05
205527_s_at	1,42	0,001569	1,64	6,54E-04	GEMIN4	gem (nuclear organelle) associated pro
212801_at	1,41	0,0001117	1,38	1,48E-05	CIT	citron (rho-interacting, serine/threonine
206838_at	1,40	1,77E-05	1,49	1,69E-04	TBX19	T-box 19
213654_at	1,39	0,0007462	1,21	0,01	TAF5L	TAF5-like RNA polymerase II, p300/CB
205521_at	1,39	8,79E-05	1,40	3,25E-03		endonuclease G-like 1
204073_s_at	1,36	0,0015862	1,44	3,09E-03	C11orf9	chromosome 11 open reading frame 9
36888_at	1,35	7,61E-05	1,37	2,35E-03	KIAA0841	KIAA0841
213889_at	1,35	6,90E-06	1,28	1,31E-03	PIGL	phosphatidylinositol glycan, class L
206498_at	1,35	2,32E-05	1,30	6,45E-03	OCA2	oculocutaneous albinism II (pink-eye di
209193_at	1,34	0,0002963	1,32	7,06E-03		
206034_at	1,29	0,0002984	1,18	0,02	SERPINB8	serine (or cysteine) proteinase inhibitor,
208212_s_at	1,26	0,0006316	1,33	3,35E-03	ALK	anaplastic lymphoma kinase (Ki-1)
209195_s_at	0,76	0,0006444	0,77	0,01	ADCY6	adenylate cyclase 6
211855_s_at	0,76	0,0009006	0,73	2,91E-03	SLC25A14	solute carrier family 25 (mitochondrial c
217549_at	0,73	0,002122	0,68	4,88E-03		
200663_at	0,70	0,0006914	0,70	0,02	CD63	CD63 antigen (melanoma 1 antigen)
208655_at	0,68	0,0010297	0,60	1,36E-04	CCNI	cyclin I
49485_at	0,68	0,0001328	0,76	6,89E-03	PRDM4	PR domain containing 4
207801_s_at	0,67	3,59E-05	0,69	0,02	RNF10	ring finger protein 10
214733_s_at	0,67	0,0010444	0,56	7,81E-04	DJ167A19.1	hypothetical protein DJ167A19.1
203144_s_at	0,66	0,0019749	0,83	0,03	KIAA0040	KIAA0040 gene product
217914_at	0,66	0,0009613	0,77	0,01		two pore segment channel 1
209110_s_at	0,65	0,0014455	0,72	0,03	RGL2	ral guanine nucleotide dissociation stim
201805_at	0,64	0,0006955	0,66	2,22E-03	PRKAG1	protein kinase, AMP-activated, gamma
219327_s_at	0,64	0,0020315	0,53	7,25E-03	GPRC5C	
209625_at	0,64	0,0012404	0,79	0,01	PIGH	phosphatidylinositol glycan, class H
219223_at	0,63	0,0019936	0,57	4,60E-03	C9orf7	chromosome 9 open reading frame 7
220319_s_at	0,63	0,0003635	0,74	5,98E-03	MYLIP	myosin regulatory light chain interacting
202596_at	0,62	0,0002086	0,76	0,01	ENSA	endosulfine alpha
50965_at	0,62	0,0004024	0,73	2,03E-03	RAB26	RAB26, member RAS oncogene family
213885_at	0,62	0,0003516	0,75	0,01	TRIM3	tripartite motif-containing 3
217726_at	0,61	5,03E-05	0,71	4,83E-03	COPZ1	coatomer protein complex, subunit zeta
211059_s_at	0,61	0,0004253	0,78	0,04	GOLGA2	golgi autoantigen, golgin subfamily a, 2
209049_s_at	0,60	0,0009248	0,49	3,03E-03	PRKCBP1	protein kinase C binding protein 1
203003_at	0,60	0,0001294	0,79	0,04	MEF2D	MADS box transcription enhancer facto
202105_at	0,59	0,000891	0,67	6,38E-03	IGBP1	immunoglobulin (CD79A) binding protei

200804_at	0,58	4,58E-05	0,65	0,02	TEGT	testis enhanced gene transcript (BAX ir
218693_at	0,57	0,0005017	0,67	0,01	TM4SF15	transmembrane 4 superfamily member
218218_at	0,57	0,0004393	0,50	6,23E-05	DIP13B	DIP13 beta
212181_s_at	0,57	0,0019529	0,40	7,23E-05	NUDT4	nudix (nucleoside diphosphate linked m
218509_at	0,56	8,00E-05	0,63	1,81E-03	LPPR2	lipid phosphate phosphatase-related pr
210720_s_at	0,56	0,0009724	0,62	7,45E-03	APBA2BP	amyloid beta (A4) precursor protein-bin
202908_at	0,56	0,0001065	0,61	2,96E-03	WFS1	Wolfram syndrome 1 (wolframin)
207105_s_at	0,55	4,10E-06	0,72	0,01	PIK3R2	phosphoinositide-3-kinase, regulatory s
218483_s_at	0,55	4,80E-06	0,70	0,02	FLJ21827	hypothetical protein FLJ21827
205766_at	0,53	0,0005532	0,53	1,99E-03	TCAP	titin-cap (telethonin)
216092_s_at	0,53	4,72E-05	0,61	7,52E-03	SLC7A8	solute carrier family 7 (cationic amino a
219734_at	0,53	3,21E-05	0,36	3,66E-05	SIDT1	SID1 transmembrane family, member 1
213846_at	0,53	0,0011658	0,61	1,75E-03	COX7C	cytochrome c oxidase subunit VIIc
211938_at	0,52	0,0020686	0,54	1,93E-03	EIF4B	eukaryotic translation initiation factor 4B
201061_s_at	0,52	0,0011679	0,72	3,40E-03	STOM	stomatin
221081_s_at	0,52	0,0001552	0,60	0,02	FLJ22457	hypothetical protein FLJ22457
219268_at	0,51	0,0009576	0,67	8,28E-03	ETNK2	ethanolamine kinase 2
211937_at	0,51	0,0002431	0,65	0,02	EIF4B	eukaryotic translation initiation factor 4B
51158_at	0,51	0,0009921	0,65	0,02	LOC400451	hypothetical gene supported by AK075
210831_s_at	0,50	0,00156	0,73	0,01	PTGER3	prostaglandin E receptor 3 (subtype EP
201236_s_at	0,48	0,0004511	0,64	0,02	BTG2	BTG family, member 2
212256_at	0,48	8,47E-05	0,56	0,01	GALNT10	UDP-N-acetyl-alpha-D-galactosamine:p
209747_at	0,47	3,22E-05	0,63	8,38E-03	TGFB3	transforming growth factor, beta 3
219648_at	0,45	0,0001786	0,69	0,01	FLJ10116	hypothetical protein FLJ10116
218035_s_at	0,44	0,0007095	0,48	7,49E-04	FLJ20273	RNA-binding protein
212960_at	0,43	0,0009082	0,66	9,69E-03	KIAA0882	KIAA0882 protein
217979_at	0,43	6,00E-07	0,47	9,70E-06	TM4SF13	transmembrane 4 superfamily member
219599_at	0,41	0,00061	0,71	0,04		
203143_s_at	0,41	8,10E-06	0,62	1,75E-03	KIAA0040	KIAA0040 gene product
211596_s_at	0,41	0,0012537	0,53	9,78E-04	LRIG1	leucine-rich repeats and immunoglobuli
209710_at	0,40	1,97E-05	0,46	1,82E-03	GATA2	GATA binding protein 2
219127_at	0,39	1,45E-05	0,47	4,97E-03	MGC11242	hypothetical protein MGC11242
200811_at	0,39	1,50E-06	0,55	6,55E-03	CIRBP	cold inducible RNA binding protein
201349_at	0,39	0,0005414	0,38	6,97E-03	SLC9A3R1	solute carrier family 9 (sodium/hydroge
206401_s_at	0,39	0,000126	0,71	0,01	MAPT	microtubule-associated protein tau
201508_at	0,39	0,0002697	0,54	0,02	IGFBP4	insulin-like growth factor binding protein
212510_at	0,38	6,70E-06	0,44	2,97E-05	GPD1L	glycerol-3-phosphate dehydrogenase 1
211896_s_at	0,38	0,0020062	0,38	3,55E-03	DCN	decorin
218086_at	0,38	0,0001239	0,40	8,16E-04	NPDC1	neural proliferation, differentiation and c
212736_at	0,37	0,0002499	0,62	5,30E-03	BC008967	hypothetical gene BC008967
218806_s_at	0,36	0,0010947	0,38	3,86E-03	VAV3	vav 3 oncogene
205186_at	0,36	1,25E-05	0,41	0,01	DNALI1	dynein, axonemal, light intermediate po
218309_at	0,34	0,0012788	0,29	1,89E-03	CaMKIINalpha	calcium/calmodulin-dependent protein k
218807_at	0,33	0,0008455	0,44	8,35E-03	VAV3	vav 3 oncogene
209696_at	0,30	8,90E-06	0,42	7,56E-03	FBP1	fructose-1,6-bisphosphatase 1
209114_at	0,29	0,0001194	0,42	0,03	TSPAN-1	tetraspan 1
203789_s_at	0,29	0,000191	0,37	5,61E-03	SEMA3C	sema domain, immunoglobulin domain
200670_at	0,29	p < 1e-07	0,27	1,85E-05	XBP1	X-box binding protein 1
211110_s_at	0,28	3,00E-06	0,46	8,31E-04	AR	androgen receptor (dihydrotestosterone
203453_at	0,27	0,0001422	0,39	0,01	SCNN1A	sodium channel, nonvoltage-gated 1 alp
206059_at	0,26	0,0009836	0,20	2,87E-05	ZNF91	zinc finger protein 91 (HPF7, HTF10)
204798_at	0,23	4,35E-05	0,45	0,03	MYB	v-myb myeloblastosis viral oncogene ho
219956_at	0,23	0,0007883	0,22	5,13E-05	GALNT6	UDP-N-acetyl-alpha-D-galactosamine:p
202089_s_at	0,21	0,0007288	0,46	0,02	SLC39A6	solute carrier family 39 (zinc transporter
205597_at	0,21	3,48E-05	0,30	0,01	C6orf29	chromosome 6 open reading frame 29
218976_at	0,21	3,30E-05	0,54	6,33E-03	DNAJC12	DnaJ (Hsp40) homolog, subfamily C, m
218211_s_at	0,18	p < 1e-07	0,31	1,33E-04	MLPH	melanophilin
204540_at	0,17	0,0006081	0,52	0,04	EEF1A2	eukaryotic translation elongation factor
208451_s_at	0,14	1,00E-07	0,34	4,08E-03	C4A /// C4B	complement component 4A
214440_at	0,11	0,000407	0,53	5,03E-03	NAT1	N-acetyltransferase 1 (arylamine N-ace
204667_at	0,08	p < 1e-07	0,10	p < 1e-07	FOXA1	forkhead box A1
205009_at	0,05	4,40E-05	0,29	1,62E-03	TFF1	trefoil factor 1 (breast cancer, estrogen-
206378_at	0,04	0,000546	0,06	8,02E-03	SCGB2A2	secretoglobin, family 2A, member 2
209173_at	0,03	p < 1e-07	0,06	1,21E-05	AGR2	anterior gradient 2 homolog (Xenopus l
