# Supplementary material for: Exquisite Sensitivity of TP53 Mutant and Basal Breast Cancers to a Dose-Dense Epirubicin−Cyclophosphamide Regimen
Source: PLoS Med. 2007 Mar 20;4(3):e90. doi: 10.1371/journal.pmed.0040090 (PMC1831731; doi:10.1371/journal.pmed.0040090)
Supplement: Table S5 — For details, see legend of Table S3. (59 KB PDF) [file pmed.0040090.st005.pdf]

Probe set	C3/C1	C1vC3 p-value	C3/C2	C2vC3 p-value	Gene Symbol	Description
202917_s_at	**19,93**	3,50E-06	**6,67**	0,01	S100A8	S100 calcium binding protein A8 (calgranulin A)
205916_at	**15,71**	0,0001588	**15,00**	7,25E-03	S100A7	S100 calcium binding protein A7 (psoriasin 1)
217528_at	**9,81**	1,00E-07	**11,42**	6,05E-04	CLCA2	chloride channel, calcium activated, family member 2
203535_at	**9,45**	0,0001029	**4,60**	0,03	S100A9	S100 calcium binding protein A9 (calgranulin B)
206165_s_at	**9,34**	p < 1e-07	**9,47**	5,90E-04	CLCA2	chloride channel, calcium activated, family member 2
207802_at	**7,82**	0,0002749	**11,28**	4,62E-03	CRISP3	cysteine-rich secretory protein 3
206166_s_at	**7,63**	p < 1e-07	**7,51**	7,74E-04	CLCA2	chloride channel, calcium activated, family member 2
216836_s_at	**7,23**	1,40E-06	**11,71**	8,27E-05	ERBB2	v-erb-b2 erythroblastic leukemia viral oncogene homolog 2
214777_at	**6,64**	0,0001627	**5,51**	4,94E-03		Immunoglobulin kappa light chain VKJ region mRNA, partia
215214_at	**6,18**	2,06E-05	**3,64**	0,02	IGLC2	immunoglobulin lambda constant 2 (Kern-Oz- marker)
206793_at	**6,15**	3,02E-05	**8,02**	5,40E-03	PNMT	phenylethanolamine N-methyltransferase
217388_s_at	**5,98**	2,10E-06	**3,65**	4,59E-03	KYNU	kynureninase (L-kynurenine hydrolase)
210930_s_at	**5,82**	4,34E-05	**7,58**	1,15E-03	ERBB2	v-erb-b2 erythroblastic leukemia viral oncogene homolog 2
219612_s_at	**5,59**	0,0017943	**7,88**	0,03	FGG	fibrinogen, gamma polypeptide
210761_s_at	**5,42**	1,00E-06	**4,98**	1,09E-03	GRB7	growth factor receptor-bound protein 7
208083_s_at	**5,07**	1,36E-05	**4,30**	9,31E-03	ITGB6	integrin, beta 6
211138_s_at	**4,07**	1,90E-05	**4,44**	5,15E-05	KMO	kynurenine 3-monooxygenase (kynurenine 3-hydroxylase)
204818_at	**3,98**	1,10E-05	**3,53**	0,01	HSD17B2	hydroxysteroid (17-beta) dehydrogenase 2
205267_at	**3,85**	7,05E-05	**2,97**	8,54E-03	POU2AF1	POU domain, class 2, associating factor 1
216829_at	**3,75**	3,01E-05	**3,39**	7,23E-03	IGKC	immunoglobulin kappa constant
206164_at	**3,63**	8,00E-07	**4,28**	1,15E-03	CLCA2	chloride channel, calcium activated, family member 2
204941_s_at	**3,21**	0,0016419	**4,41**	2,20E-03	ALDH3B2	aldehyde dehydrogenase 3 family, member B2
212311_at	**2,98**	1,03E-05	**1,88**	0,02	KIAA0746	KIAA0746 protein
203767_s_at	**2,89**	3,34E-05	**2,95**	5,14E-03	STS	steroid sulfatase (microsomal), arylsulfatase C, isozyme S
212325_at	**2,84**	6,70E-06	**2,67**	6,40E-03	KIAA1102	KIAA1102 protein
210663_s_at	**2,83**	4,50E-06	**2,84**	1,18E-03	KYNU	kynureninase (L-kynurenine hydrolase)
55616_at	**2,83**	0,0002087	**3,80**	1,50E-03	PERLD1	per1-like domain containing 1
221811_at	**2,79**	0,0003469	**4,19**	1,20E-03	PERLD1	per1-like domain containing 1
200730_s_at	**2,79**	0,0006252	**1,83**	0,04	PTP4A1	protein tyrosine phosphatase type IVA, member 1
208840_s_at	**2,73**	0,0002022	**2,07**	0,04	G3BP2	Ras-GTPase activating protein SH3 domain-binding proteir
201670_s_at	**2,69**	0,0003542	**2,18**	4,04E-04	MARCKS	myristoylated alanine-rich protein kinase C substrate
208084_at	**2,68**	5,69E-05	**2,46**	0,01	ITGB6	integrin, beta 6
200727_s_at	**2,67**	0,0008502	**1,96**	6,28E-03	ACTR2	ARP2 actin-related protein 2 homolog (yeast)
204942_s_at	**2,56**	0,0018467	**5,94**	7,39E-05	ALDH3B2	aldehyde dehydrogenase 3 family, member B2
203740_at	**2,54**	4,00E-07	**2,05**	3,77E-03	MPHOSPH6	M-phase phosphoprotein 6
202991_at	**2,36**	1,69E-05	**3,10**	1,22E-03	STARD3	START domain containing 3
219551_at	**2,33**	2,55E-05	**2,39**	4,95E-03	EAF2	ELL associated factor 2
201669_s_at	**2,32**	2,80E-06	**1,36**	0,02	MARCKS	myristoylated alanine-rich protein kinase C substrate
214446_at	**2,31**	0,0006656	**2,93**	9,18E-04	ELL2	elongation factor, RNA polymerase II, 2
215501_s_at	**2,24**	0,0015533	**2,60**	5,48E-05	DUSP10	dual specificity phosphatase 10
204132_s_at	**2,22**	4,53E-05	**1,54**	0,01	FOXO3A	forkhead box O3A
207734_at	**2,18**	9,88E-05	**2,08**	9,84E-03	LAX	hypothetical protein FLJ20340
201862_s_at	**2,17**	0,0006559	**2,02**	3,84E-03	LRRFIP1	leucine rich repeat (in FLII) interacting protein 1
204385_at	**2,14**	1,60E-06	**1,98**	4,81E-03	KYNU	kynureninase (L-kynurenine hydrolase)
203722_at	**2,06**	0,0004169	**2,44**	3,09E-03	ALDH4A1	aldehyde dehydrogenase 4 family, member A1
212611_at	**2,05**	0,0006428	**2,06**	0,01	DTX4	deltex 4 homolog (Drosophila)
211612_s_at	**2,03**	0,0007023	**2,50**	2,45E-04	IL13RA1	interleukin 13 receptor, alpha 1 /// interleukin 13 receptor, a
216607_s_at	**2,02**	0,0011431	**1,79**	0,01	CYP51A1	cytochrome P450, family 51, subfamily A, polypeptide 1
205248_at	**2,01**	0,0007228	**2,27**	2,18E-03	C21orf5	chromosome 21 open reading frame 5
218228_s_at	**2,01**	0,0001923	**2,02**	9,01E-04	TNKS2	tankyrase, TRF1-interacting ankyrin-related ADP-ribose pol
208997_s_at	**2,00**	0,0004579	**2,97**	3,84E-03	UCP2	uncoupling protein 2 (mitochondrial, proton carrier)
205150_s_at	**2,00**	0,0015589	**2,70**	1,12E-03	KIAA0644	KIAA0644 gene product
202478_at	**1,95**	3,90E-05	**1,60**	0,02	TRIB2	tribbles homolog 2 (Drosophila)
211317_s_at	**1,94**	0,000489	**1,91**	0,01	CFLAR	CASP8 and FADD-like apoptosis regulator
200890_s_at	**1,94**	0,0004676	**1,42**	6,88E-03	SSR1	signal sequence receptor, alpha (translocon-associated prc
212902_at	**1,93**	5,49E-05	**1,71**	0,01	SEC24A	SEC24 related gene family, member A (S. cerevisiae)
204562_at	**1,93**	0,0003998	**1,81**	0,02	IRF4	interferon regulatory factor 4
201204_s_at	**1,93**	0,0004024	**1,73**	4,43E-03	RRBP1	ribosome binding protein 1 homolog 180kDa (dog)
200825_s_at	**1,92**	0,0001201	**1,44**	0,01	HYOU1	hypoxia up-regulated 1
221563_at	**1,92**	0,0008559	**1,87**	2,37E-03	DUSP10	dual specificity phosphatase 10
202195_s_at	**1,92**	4,80E-05	**1,63**	3,61E-03	CGI-100	CGI-100 protein
211742_s_at	**1,90**	0,0013388	**1,62**	0,04	EVI2B	ecotropic viral integration site 2B /// ecotropic viral integratic
205351_at	**1,82**	8,95E-05	**1,58**	0,02	GGCX	gamma-glutamyl carboxylase
209186_at	**1,80**	0,0007813	**1,51**	0,01	ATP2A2	ATPase, Ca++ transporting, cardiac muscle, slow twitch 2
202067_s_at	**1,79**	0,0001686	**1,63**	7,37E-03	LDLR	low density lipoprotein receptor (familial hypercholesteroler
219155_at	**1,75**	0,0018147	**1,92**	0,01	PITPNC1	phosphatidylinositol transfer protein, cytoplasmic 1
209091_s_at	**1,74**	0,001123	**1,55**	4,83E-03	SH3GLB1	SH3-domain GRB2-like endophilin B1
208119_s_at	**1,73**	2,31E-05	**1,60**	0,02	ZNF505	zinc finger protein 505 /// zinc finger protein 505
200953_s_at	**1,73**	0,0003781	**2,66**	6,98E-04	CCND2	cyclin D2
218113_at	**1,71**	0,0004672	**1,51**	0,01	TMEM2	transmembrane protein 2
210555_s_at	**1,69**	1,54E-05	**1,32**	0,03	NFATC3	nuclear factor of activated T-cells, cytoplasmic, calcineurin-
219033_at	**1,66**	0,0003211	**1,89**	6,96E-05	PARP8	poly (ADP-ribose) polymerase family, member 8
217168_s_at	**1,65**	0,0002648	**1,58**	2,41E-03	HERPUD1	homocysteine-inducible, endoplasmic reticulum stress-indu
212415_at	**1,63**	0,0006001	**1,65**	2,80E-03	6-sep	septin 6
204362_at	**1,62**	0,000738	**1,51**	0,02	SCAP2	src family associated phosphoprotein 2

218960_at	**1,61**	0,0017381	**1,69**	0,02	TMPRSS4	transmembrane protease, serine 4
208033_s_at	**1,55**	0,0014384	**1,69**	1,71E-03	ATBF1	AT-binding transcription factor 1
221090_s_at	**1,54**	0,0001552	**1,33**	0,02	FLJ10826	hypothetical protein FLJ10826
208743_s_at	**1,53**	0,0007387	**1,29**	5,32E-03	YWHAB	tyrosine 3-monooxygenase/tryptophan 5-monooxygenase a
202702_at	**1,52**	0,0002541	**1,24**	0,04	TRIM26	tripartite motif-containing 26
219628_at	**1,52**	0,0003456	**1,45**	0,01	WIG1	p53 target zinc finger protein
212293_at	**1,51**	0,0004013	**1,36**	0,01	HIPK1	homeodomain interacting protein kinase 1
219865_at	**1,51**	0,0006032	**1,52**	0,02	HSPC157	HSPC157 protein
218324_s_at	**1,49**	0,0010441	**1,60**	0,01	SPATS2	spermatogenesis associated, serine-rich 2
219397_at	**1,48**	0,0008397	**1,70**	1,10E-03	FLJ13448	hypothetical protein FLJ13448
218562_s_at	**1,48**	0,0009339	**1,75**	7,76E-03	FLJ10747	hypothetical protein FLJ10747
215044_s_at	**1,47**	9,17E-05	**1,38**	9,31E-03	STAM2	signal transducing adaptor molecule (SH3 domain and ITAI
221937_at	**1,47**	0,0014225	**1,66**	0,01	AP1GBP1	AP1 gamma subunit binding protein 1
37831_at	**1,46**	0,0007042	**1,52**	8,55E-03	SIPA1L3	signal-induced proliferation-associated 1 like 3
218810_at	**1,45**	0,0007758	**1,43**	0,02	FLJ23231	hypothetical protein FLJ23231
217893_s_at	**1,42**	0,0006359	**1,58**	5,63E-03	FLJ12666	hypothetical protein FLJ12666
212072_s_at	**1,40**	0,0010705	**1,29**	3,45E-03	CSNK2A1	casein kinase 2, alpha 1 polypeptide
218076_s_at	**1,39**	9,70E-06	**1,41**	3,96E-03	ARHGAP17	Rho GTPase activating protein 17
219784_at	**0,84**	0,0005537	**0,77**	7,49E-03	FBXO31	F-box protein 31
208359_s_at	**0,82**	0,002498	**0,72**	6,01E-04	KCNJ4	potassium inwardly-rectifying channel, subfamily J, membe
205305_at	**0,81**	0,0014735	**0,83**	9,28E-03	FGL1	fibrinogen-like 1
213066_at	**0,81**	0,0008979	**0,76**	3,96E-04	RUSC2	RUN and SH3 domain containing 2
217205_at	**0,80**	0,0002861	**0,85**	0,03		
207690_at	**0,79**	0,0004309	**0,81**	6,00E-04	ALX3	aristaless-like homeobox 3
52741_at	**0,78**	5,61E-05	**0,83**	0,02	FLJ40452	hypothetical protein FLJ40452
217381_s_at	**0,78**	0,0005856	**0,83**	0,02		Full-length cDNA clone CS0DJ012YJ02 of T cells (Jurkat c
218372_at	**0,77**	9,10E-06	**0,78**	0,01	MED25	mediator subunit 25
211500_at	**0,77**	0,0003602	**0,78**	1,59E-03	MAPK11	mitogen-activated protein kinase 11
215367_at	**0,77**	0,0005108	**0,86**	5,17E-03	KIAA1614	KIAA1614 protein
202182_at	**0,76**	0,0010997	**0,71**	4,65E-03	GCN5L2	GCN5 general control of amino-acid synthesis 5-like 2 (yea
211028_s_at	**0,75**	0,0001049	**0,71**	1,23E-04	KHK	ketohexokinase (fructokinase) /// ketohexokinase (fructokin
221109_at	**0,75**	3,30E-06	**0,81**	3,65E-03		hypothetical protein DKFZp434C0923
202248_at	**0,74**	3,60E-06	**0,84**	0,04	E2F4	E2F transcription factor 4, p107/p130-binding
219364_at	**0,74**	0,0001176	**0,75**	9,86E-03	LGP2	likely ortholog of mouse D11lgp2
204706_at	**0,72**	0,0008932	**0,65**	1,68E-04	INPP5E	inositol polyphosphate-5-phosphatase, 72 kDa
203171_s_at	**0,72**	0,000137	**0,85**	0,01	KIAA0409	KIAA0409 protein
210373_at	**0,71**	1,08E-05	**0,84**	0,03	TUSC4	tumor suppressor candidate 4
206298_at	**0,71**	0,0001183	**0,49**	4,71E-04	ARHGAP22	Rho GTPase activating protein 22
206814_at	**0,71**	0,0001523	**0,78**	0,01	NGFB	nerve growth factor, beta polypeptide
209940_at	**0,71**	0,0002447	**0,74**	0,01	PARP3	poly (ADP-ribose) polymerase family, member 3
201470_at	**0,70**	0,0006444	**0,72**	3,50E-03	GSTO1	glutathione S-transferase omega 1
214674_at	**0,69**	9,73E-05	**0,83**	0,02	USP19	ubiquitin specific protease 19
52159_at	**0,69**	0,0001779	**0,81**	2,86E-03	HEMK1	HemK methyltransferase family member 1
212803_at	**0,69**	0,0001472	**0,72**	3,55E-03	NAB2	NGFI-A binding protein 2 (EGR1 binding protein 2)
202243_s_at	**0,68**	0,0013631	**0,58**	3,11E-04	PSMB4	proteasome (prosome, macropain) subunit, beta type, 4
206441_s_at	**0,68**	5,38E-05	**0,71**	0,01	COMMD4	COMM domain containing 4
214338_at	**0,67**	8,05E-05	**0,79**	0,01	DNAJB12	DnaJ (Hsp40) homolog, subfamily B, member 12
221096_s_at	**0,67**	6,26E-05	**0,59**	6,29E-04	PRO1580	hypothetical protein PRO1580
205905_s_at	**0,67**	2,50E-06	**0,76**	0,04	MICA /// MICB	MHC class I polypeptide-related sequence A /// MHC class
205086_s_at	**0,66**	0,0003889	**0,63**	3,10E-03	384D8-2	hypothetical protein 384D8_6
218664_at	**0,65**	0,0002926	**0,70**	0,02	CGI-63	nuclear receptor binding factor 1
203246_s_at	**0,65**	0,0016892	**0,81**	0,03	TUSC4	tumor suppressor candidate 4
218590_at	**0,64**	0,0009672	**0,58**	8,34E-04	PEO1	progressive external ophthalmoplegia 1
213256_at	**0,64**	0,0020944	**0,33**	3,60E-04	MARCH-III	hypothetical protein MGC48332
204121_at	**0,64**	0,0010808	**0,81**	9,86E-03	GADD45G	growth arrest and DNA-damage-inducible, gamma
213468_at	**0,64**	1,60E-05	**0,62**	6,39E-03	ERCC2	excision repair cross-complementing rodent repair deficien
205224_at	**0,64**	0,0004458	**0,77**	0,02	SURF2	surfeit 2
209718_at	**0,63**	1,30E-06	**0,72**	2,25E-03	384D8-2	hypothetical protein 384D8_6
218270_at	**0,62**	0,0012833	**0,74**	0,02	MRPL24	mitochondrial ribosomal protein L24
213053_at	**0,61**	1,03E-05	**0,66**	6,59E-03	KIAA0841	KIAA0841
218722_s_at	**0,61**	0,0001615	**0,72**	9,64E-03	FLJ12436	hypothetical protein FLJ12436
203488_at	**0,60**	2,36E-05	**0,79**	0,04	LPHN1	latrophilin 1
221649_s_at	**0,60**	6,00E-07	**0,72**	4,74E-03	PPAN	peter pan homolog (Drosophila)
203045_at	**0,59**	0,0004161	**0,76**	0,01	NINJ1	ninjurin 1
213520_at	**0,57**	2,80E-05	**0,59**	3,18E-03		
214919_s_at	**0,57**	0,0004196	**0,70**	0,01	EIF4EBP3 /// I	MASK-4E-BP3 alternate reading frame gene /// eukaryotic
203655_at	**0,55**	1,61E-05	**0,71**	0,03	XRCC1	X-ray repair complementing defective repair in Chinese har
202387_at	**0,51**	0,0003334	**0,73**	0,01	BAG1	BCL2-associated athanogene
204343_at	**0,48**	9,74E-05	**0,63**	0,01	ABCA3	ATP-binding cassette, sub-family A (ABC1), member 3
218862_at	**0,48**	0,0002035	**0,45**	7,63E-05	ASB13	ankyrin repeat and SOCS box-containing 13
204497_at	**0,47**	0,0004747	**0,63**	5,02E-03	ADCY9	adenylate cyclase 9
212551_at	**0,45**	3,41E-05	**0,50**	7,94E-03	CAP2	CAP, adenylate cyclase-associated protein, 2 (yeast)
209339_at	**0,45**	0,0009976	**0,63**	2,52E-03	SIAH2	seven in absentia homolog 2 (Drosophila)
203071_at	**0,44**	0,0001614	**0,66**	0,03	SEMA3B	sema domain, immunoglobulin domain (Ig), short basic dor
218094_s_at	**0,38**	4,00E-05	**0,63**	6,74E-03	C20orf35	chromosome 20 open reading frame 35
207414_s_at	**0,34**	0,0001331	**0,45**	3,53E-04	PCSK6	proprotein convertase subtilisin/kexin type 6

203439_s_at	**0,24**	1,64E-05	**0,68**	4,97E-03	STC2	stanniocalcin 2
203438_at	**0,19**	0,0002526	**0,43**	3,29E-04	STC2	stanniocalcin 2
206754_s_at	**0,14**	0,0002216	**0,81**	0,02	CYP2B6	cytochrome P450, family 2, subfamily B, polypeptide 6
202376_at	**0,13**	5,50E-06	**0,27**	7,03E-03	SERPINA3	serine (or cysteine) proteinase inhibitor, clade A (alpha-1 ar
205440_s_at	**0,13**	0,0009498	**0,51**	0,01		neuropeptide Y receptor Y1
