# Supplementary material for: Exquisite Sensitivity of TP53 Mutant and Basal Breast Cancers to a Dose-Dense Epirubicin−Cyclophosphamide Regimen
Source: PLoS Med. 2007 Mar 20;4(3):e90. doi: 10.1371/journal.pmed.0040090 (PMC1831731; doi:10.1371/journal.pmed.0040090)
Supplement: Table S6 — Probe sets associated with TP53 status are indicated. The false discovery rate in this experiment is 26%. (26 KB PDF) [file pmed.0040090.st006.pdf]

p53genes	Probe set	compl/incompl	compl/incompl p value	Gene Symbol
	206165_s_at	4,91	9,58E-04	CLCA2
yes	209016_s_at	4	9,22E-04	KRT7
	204268_at	2,62	8,68E-04	S100A2
	202218_s_at	2,36	1,21E-04	FADS2
yes	214953_s_at	2,33	2,39E-04	APP
yes	209916_at	2,13	4,33E-04	DHTKD1
	209373_at	1,85	6,09E-04	BENE
yes	218856_at	1,76	8,33E-04	TNFRSF21
	220230_s_at	1,65	4,73E-04	CYB5R2
yes	204799_at	1,59	7,73E-04	---
	202529_at	1,56	5,68E-04	PRPSAP1
yes	209214_s_at	1,55	2,23E-04	EWSR1
	214058_at	1,48	7,10E-04	---
	204124_at	1,45	8,43E-04	---
	203779_s_at	1,43	2,32E-04	EVA1
yes	202067_s_at	1,41	4,09E-04	LDLR
	206533_at	1,4	4,93E-04	CHRNA5
yes	210114_at	1,39	3,35E-04	INVS
yes	203664_s_at	1,39	1,65E-04	POLR2D
	210119_at	1,32	2,14E-04	KCNJ15
	209558_s_at	1,31	6,07E-04	HIP1R
yes	31637_s_at	1,28	4,51E-04	THRA /// NR1D1
	219820_at	1,22	4,82E-04	SLC6A16
	219925_at	0,85	9,15E-04	ZNF258
yes	215152_at	0,84	5,38E-04	MYB
	207381_at	0,83	5,25E-04	ALOX12B
	215830_at	0,8	7,30E-04	---
yes	210623_at	0,79	6,82E-04	LOC51035
yes	220849_at	0,78	2,03E-04	FLJ22659
yes	218978_s_at	0,78	5,41E-04	MSCP
yes	211591_s_at	0,78	8,67E-04	PDE4A
yes	202977_s_at	0,78	8,47E-04	ZF
	216427_at	0,77	9,51E-04	---
yes	219986_s_at	0,76	4,50E-06	ACAD10
yes	204582_s_at	0,76	9,02E-05	KLK3
yes	216711_s_at	0,76	2,79E-04	TAF1
yes	217413_s_at	0,76	9,85E-04	TNXB
	214579_at	0,76	8,74E-04	DJ462O23.2
	33850_at	0,75	6,27E-04	MAP4
	213987_s_at	0,75	9,51E-04	---
yes	214619_at	0,73	7,11E-04	CRHR1
yes	220907_at	0,73	9,17E-04	FLJ22684
yes	204889_s_at	0,73	3,62E-04	NEURL
	218705_s_at	0,73	4,47E-04	SNX24
yes	219830_at	0,72	9,02E-04	RAI1
yes	215676_at	0,71	7,40E-04	BRF1
	207123_s_at	0,71	5,88E-04	MATN4
	218965_s_at	0,71	8,04E-04	RBM21
yes	221309_at	0,7	8,05E-04	RBM17
yes	217585_at	0,69	9,21E-04	NEBL
yes	210161_at	0,68	9,78E-04	---
yes	207004_at	0,68	5,28E-04	BCL2
yes	207416_s_at	0,68	5,36E-04	NFATC3
yes	203280_at	0,68	1,18E-04	SAFB2
yes	222289_at	0,67	7,82E-04	---
yes	204954_s_at	0,67	5,30E-04	DYRK1B
yes	213731_s_at	0,66	9,15E-04	---

yes	216485_s_at	0,65	9,94E-04	---
yes	208474_at	0,65	3,73E-04	CLDN6
yes	207530_s_at	0,64	3,70E-04	CDKN2B
yes	216835_s_at	0,64	8,98E-04	DOK1
yes	205211_s_at	0,64	6,05E-04	RIN1
yes	221071_at	0,63	6,90E-04	---
yes	217660_at	0,63	3,26E-04	MYH14
yes	214105_at	0,62	3,59E-04	---
yes	217468_at	0,62	5,47E-04	---
yes	201452_at	0,62	4,95E-04	RHEB
yes	204876_at	0,61	5,91E-04	ZNF646
yes	206781_at	0,59	2,64E-04	DNAJC4
yes	213740_s_at	0,58	3,38E-04	ZFPL1
yes	221013_s_at	0,57	8,74E-04	APOL2
yes	213690_s_at	0,56	6,03E-04	---
yes	215616_s_at	0,56	1,56E-04	JMJD2B
yes	221065_s_at	0,55	5,79E-04	CHST8
yes	206416_at	0,53	2,83E-04	ZNF205
yes	206328_at	0,52	5,58E-04	CDH15
yes	205225_at	0,14	1,88E-04	ESR1
