# Supplementary material for: Exquisite Sensitivity of TP53 Mutant and Basal Breast Cancers to a Dose-Dense Epirubicin−Cyclophosphamide Regimen
Source: PLoS Med. 2007 Mar 20;4(3):e90. doi: 10.1371/journal.pmed.0040090 (PMC1831731; doi:10.1371/journal.pmed.0040090)
Supplement: Table S7 — Fold ratio and p-values are indicated. A total of 1,599 significant genes were identified. (46 KB PDF) [file pmed.0040090.st007.pdf]

Gene Title	Fold change (mut/nonmut)	Parametric p-value
prominin 1 /// prominin 1	6,25	5,54E-05
thymosin, beta, identified in neuroblastoma cells /// thymosin, beta, identified	5,56	2,11E-05
keratin 7 /// keratin 7	5,26	1,02E-05
Ras-induced senescence 1	4,00	1,39E-05
pleckstrin homology-like domain, family A, member 2 /// pleckstrin homology-	4,00	1,61E-05
v-maf musculoaponeurotic fibrosarcoma oncogene homolog F (avian)	3,85	1,22E-05
hypothetical protein FLJ10901	3,57	9,58E-05
adrenomedullin /// adrenomedullin	3,57	1,57E-05
TTK protein kinase /// TTK protein kinase	3,45	3,40E-06
CDC20 cell division cycle 20 homolog (S. cerevisiae) /// CDC20 cell division cy	3,33	1,90E-06
cyclin-dependent kinase inhibitor 2A (melanoma, p16, inhibits CDK4) /// cyclin	3,23	4,89E-04
karyopherin alpha 2 (RAG cohort 1, importin alpha 1) /// karyopherin alpha 2 (I	3,13	5,80E-06
apolipoprotein B mRNA editing enzyme, catalytic polypeptide-like 3B /// apolip	3,03	1,71E-04
ribonucleotide reductase M2 polypeptide	2,94	2,30E-04
cysteine and glycine-rich protein 2 /// cysteine and glycine-rich protein 2	2,94	4,17E-04
tenascin C (hexabrachion) /// tenascin C (hexabrachion)	2,86	1,54E-04
guanylate binding protein 1, interferon-inducible, 67kDa /// guanylate binding	2,78	3,01E-04
amyloid beta (A4) precursor protein (protease nexin-II, Alzheimer disease)	2,78	4,00E-07
gamma-glutamyl hydrolase (conjugase, folylpolygammaglutamyl hydrolase) /	2,70	5,72E-04
ubiquitin-conjugating enzyme E2E 3 (UBC4/5 homolog, yeast) /// ubiquitin-cor	2,63	5,42E-05
Mlx interactor	2,63	1,00E-09
hypothetical protein FLJ12442	2,56	2,32E-05
FAT tumor suppressor homolog 1 (Drosophila) /// FAT tumor suppressor hom	2,56	6,09E-05
ring finger and KH domain containing 1	2,50	1,40E-06
rhomboid, veinlet-like 2 (Drosophila)	2,50	3,60E-06
pleckstrin homology-like domain, family A, member 1	2,50	9,40E-04
centromere protein A, 17kDa /// centromere protein A, 17kDa	2,50	5,84E-05
cadherin 3, type 1, P-cadherin (placental) /// cadherin 3, type 1, P-cadherin (pla	2,50	9,66E-04
phosphoglycerate dehydrogenase /// phosphoglycerate dehydrogenase	2,44	1,56E-05
dehydrogenase E1 and transketolase domain containing 1	2,44	1,70E-06
TPX2, microtubule-associated protein homolog (Xenopus laevis) /// TPX2, mic	2,38	1,81E-05
transducin-like enhancer of split 1 (E(sp1) homolog, Drosophila)	2,38	9,98E-05
thrombospondin 1 /// thrombospondin 1	2,38	2,98E-04
synaptotagmin binding, cytoplasmic RNA interacting protein	2,38	1,02E-05
LIM domain only 4 /// LIM domain only 4	2,38	4,63E-04
intercellular adhesion molecule 1 (CD54), human rhinovirus receptor /// interc	2,38	4,49E-05
etoposide induced 2.4 mRNA	2,38	1,00E-09
DNA-damage-inducible transcript 4	2,38	4,88E-05
tubulin, beta polypeptide	2,33	6,16E-05
cysteine-rich, angiogenic inducer, 61 /// cysteine-rich, angiogenic inducer, 61	2,33	4,07E-05
caveolin 2	2,33	5,06E-04
transforming growth factor beta 1 induced transcript 1 /// transforming growth	2,27	2,12E-04
ornithine decarboxylase 1 /// ornithine decarboxylase 1	2,27	3,54E-04
nuclear factor, interleukin 3 regulated /// nuclear factor, interleukin 3 regulated	2,27	2,28E-04
Melanoma associated gene	2,27	1,22E-04
solute carrier family 6 (neurotransmitter transporter, creatine), member 8 /// sc	2,22	3,48E-04
molecule interacting with Rab13	2,22	1,63E-05
tubulin beta MGC4083	2,22	2,46E-04
hypothetical protein FLJ22222	2,22	2,53E-05
stromal membrane-associated protein 1	2,17	1,00E-07
NIMA (never in mitosis gene a)-related kinase 2 /// NIMA (never in mitosis gene	2,17	2,16E-04
N-myc downstream regulated gene 1 /// N-myc downstream regulated gene 1	2,17	7,08E-04
midline 1 (Opitz/BBB syndrome) /// midline 1 (Opitz/BBB syndrome)	2,17	8,83E-04
hypothetical protein MGC39900	2,17	9,36E-04

**kinesin family member 20A /// kinesin family member 20A**	**2,17**	2,40E-04
**insulin-like growth factor 2 receptor /// insulin-like growth factor 2 receptor**	**2,17**	2,70E-05
**FN5 protein**	**2,17**	5,70E-06
**cold shock domain protein A**	**2,17**	1,03E-04
**collagen, type XV, alpha 1 /// collagen, type XV, alpha 1**	**2,17**	1,07E-05
**CDC28 protein kinase regulatory subunit 2 /// CDC28 protein kinase regulator**	**2,17**	4,51E-04
**cyclin B2 /// cyclin B2**	**2,17**	5,32E-04
**bicaudal D homolog 2 (Drosophila)**	**2,17**	2,68E-05
**BCL2-associated X protein**	**2,17**	4,37E-05
**nidogen (enactin)**	**2,13**	2,10E-04
**lipin 1**	**2,13**	8,85E-04
**lysyl oxidase-like 2 /// lysyl oxidase-like 2**	**2,13**	9,17E-04
**LIM protein (similar to rat protein kinase C-binding enigma) /// LIM protein (sin**	**2,13**	2,11E-04
**putative G-protein coupled receptor GPCR41**	**2,13**	3,21E-04
**related RAS viral (r-ras) oncogene homolog 2**	**2,08**	3,43E-04
**ribosomal protein L39-like**	**2,08**	4,03E-04
**quaking homolog, KH domain RNA binding (mouse)**	**2,08**	1,09E-04
**prostaglandin E receptor 4 (subtype EP4)**	**2,08**	2,73E-04
**mitogen-activated protein kinase kinase 3**	**2,08**	1,00E-09
**protein predicted by clone 23627**	**2,08**	5,30E-05
**GM2 ganglioside activator**	**2,08**	2,00E-05
**growth arrest-specific 1 /// growth arrest-specific 1**	**2,08**	7,37E-04
**drebrin 1 /// drebrin 1**	**2,08**	1,04E-04
**chemokine (C-X3-C motif) ligand 1**	**2,08**	9,65E-04
**carbohydrate (N-acetylglucosamine 6-O) sulfotransferase 6 /// carbohydrate (N**	**2,08**	9,07E-05
**BUB1 budding uninhibited by benzimidazoles 1 homolog (yeast) /// BUB1 bud**	**2,08**	8,38E-04
**RAD23 homolog B (S. cerevisiae)**	**2,04**	2,61E-04
**myristoylated alanine-rich protein kinase C substrate /// myristoylated alanine**	**2,04**	2,63E-05
**KIAA0186 gene product**	**2,04**	5,93E-04
**HSPC163 protein**	**2,04**	9,29E-05
**H2A histone family, member X**	**2,04**	3,56E-05
**cyclin B1**	**2,04**	1,43E-04
**tumor necrosis factor receptor superfamily, member 21**	**2,00**	8,90E-06
**transducin-like enhancer of split 1 (E(sp1) homolog, Drosophila)**	**2,00**	6,74E-04
**syndecan 1**	**2,00**	4,59E-04
**quaking homolog, KH domain RNA binding (mouse)**	**2,00**	9,32E-04
**karyopherin alpha 2 (RAG cohort 1, importin alpha 1) /// karyopherin alpha 2 (I**	**2,00**	9,59E-04
**dihydropyrimidinase-like 3**	**2,00**	3,60E-04
**chromosome 10 open reading frame 38**	**2,00**	7,33E-04
**tyrosine 3-monooxygenase/tryptophan 5-monooxygenase activation protein, t**	**1,96**	8,46E-04
**Rac GTPase activating protein 1**	**1,96**	3,30E-04
**phosphoserine aminotransferase 1 /// phosphoserine aminotransferase 1**	**1,96**	8,57E-04
**KIAA0657 protein**	**1,96**	8,64E-04
**UDP-N-acetyl-alpha-D-galactosamine:polypeptide N-acetylgalactosaminyltran**	**1,96**	3,08E-04
**death-associated protein kinase 1 /// death-associated protein kinase 1**	**1,96**	2,04E-04
**cysteine-rich, angiogenic inducer, 61**	**1,96**	3,34E-04
**GNAS complex locus**	**0,50**	4,10E-06
**fibroblast growth factor 18**	**0,50**	4,00E-07
**B-factor, properdin /// B-factor, properdin**	**0,50**	7,00E-07
**bobby sox homolog (Drosophila)**	**0,50**	3,80E-06
**sema domain, immunoglobulin domain (Ig), short basic domain, secreted, (se**	**0,50**	4,90E-06
**sodium channel, nonvoltage-gated 1 alpha**	**0,50**	4,00E-07
**leukocyte tyrosine kinase /// leukocyte tyrosine kinase**	**0,50**	2,10E-06
**zinc finger protein-like 1**	**0,49**	3,80E-06
**carboxylesterase 4-like /// carboxylesterase 4-like**	**0,49**	4,00E-07
**phosphodiesterase 4A, cAMP-specific (phosphodiesterase E2 dunce homolog**	**0,49**	1,00E-09
**eukaryotic translation initiation factor 4E binding protein 2 pseudogene**	**0,48**	1,00E-09
**apolipoprotein E**	**0,48**	1,00E-09

**RAS guanyl releasing protein 2 (calcium and DAG-regulated)**	**0,48**	1,00E-09
**prostate tumor overexpressed gene 1**	**0,48**	1,00E-09
**prostate tumor overexpressed gene 1**	**0,48**	1,00E-09
**ataxin 2 related protein /// ataxin 2 related protein**	**0,48**	1,00E-09
**synaptojanin 2**	**0,47**	1,00E-09
**protein kinase C-like 2**	**0,47**	1,62E-04
**interleukin 6 signal transducer (gp130, oncostatin M receptor)**	**0,47**	5,64E-05
**GABA(A) receptor-associated protein like 1**	**0,47**	1,00E-09
**cytochrome c oxidase subunit VIc /// cytochrome c oxidase subunit VIc**	**0,47**	1,84E-04
**myosin light chain 2, precursor lymphocyte-specific**	**0,47**	1,00E-09
**laminin, alpha 2 (merosin, congenital muscular dystrophy) /// laminin, alpha 2**	**0,47**	1,16E-05
**interleukin 6 signal transducer (gp130, oncostatin M receptor)**	**0,47**	1,53E-04
**adenosine deaminase, RNA-specific, B1 (RED1 homolog rat)**	**0,46**	1,00E-09
**transportin 2 (importin 3, karyopherin beta 2b)**	**0,46**	1,00E-09
**PDZ and LIM domain 7 (enigma)**	**0,46**	1,00E-09
**ems1 sequence (mammary tumor and squamous cell carcinoma-associated (**	**0,46**	1,16E-05
**cytochrome c oxidase subunit Vb**	**0,46**	1,00E-09
**apolipoprotein L, 2 /// apolipoprotein L, 2 /// apolipoprotein L, 2**	**0,46**	1,00E-09
**myeloid/lymphoid or mixed-lineage leukemia (trithorax homolog, Drosophila);**	**0,46**	1,00E-09
**C-terminal binding protein 1**	**0,46**	4,00E-07
**zinc finger protein 205 /// zinc finger protein 205**	**0,45**	1,00E-09
**regulator of G-protein signalling 6**	**0,45**	2,00E-07
**protein phosphatase 3 (formerly 2B), catalytic subunit, gamma isoform (calcir**	**0,45**	2,00E-07
**MRNA; cDNA DKFZp564F053 (from clone DKFZp564F053)**	**0,45**	7,05E-04
**tyrosine 3-monooxygenase/tryptophan 5-monooxygenase activation protein,**	**0,44**	1,28E-05
**ankyrin repeat domain 19**	**0,44**	1,00E-09
**transportin 1**	**0,44**	1,52E-05
**centaurin, beta 1**	**0,44**	1,00E-09
**translocated promoter region (to activated MET oncogene)**	**0,44**	6,65E-04
**mitogen-activated protein kinase 8 interacting protein 2 /// mitogen-activated**	**0,44**	3,50E-06
**eukaryotic translation initiation factor 5A**	**0,44**	9,60E-05
**cadherin 15, M-cadherin (myotubule)**	**0,44**	1,00E-07
**zinc finger protein 148 (pHZ-52) /// zinc finger protein 148 (pHZ-52)**	**0,43**	1,43E-04
**B-cell CLL/lymphoma 2 /// B-cell CLL/lymphoma 2**	**0,43**	3,05E-04
**stromal antigen 2**	**0,43**	1,73E-05
**G protein-coupled receptor 144**	**0,43**	1,00E-09
**estrogen receptor 1**	**0,43**	9,80E-06
**BCL2 binding component 3 /// BCL2 binding component 3**	**0,43**	1,00E-09
**splicing factor, arginine/serine-rich 6**	**0,43**	6,19E-05
**fucosyltransferase 7 (alpha (1,3) fucosyltransferase)**	**0,43**	6,00E-07
**discs, large homolog 4 (Drosophila)**	**0,43**	1,00E-09
**chromosome 1 open reading frame 34**	**0,43**	6,60E-04
**KIAA0882 protein**	**0,42**	1,00E-09
**ATPase, H+ transporting, lysosomal 9kDa, V0 subunit e**	**0,42**	2,00E-07
**tubulin tyrosine ligase-like family, member 3**	**0,42**	1,00E-09
**ribosomal protein S20**	**0,41**	1,00E-07
**transcription factor 15 (basic helix-loop-helix) /// transcription factor 15 (basic**	**0,41**	1,00E-09
**amyotrophic lateral sclerosis 2 (juvenile) chromosome region, candidate 3**	**0,41**	9,00E-07
**hypothetical protein MGC40499**	**0,41**	1,00E-09
**eukaryotic translation initiation factor 1A, X-linked**	**0,41**	5,97E-05
**dachshund homolog 1 (Drosophila)**	**0,40**	4,00E-04
**androgen receptor (dihydrotestosterone receptor; testicular feminization; spi**	**0,40**	1,64E-04
**immunoglobulin heavy constant mu /// immunoglobulin heavy constant mu**	**0,40**	1,00E-09
**platelet-activating factor receptor /// platelet-activating factor receptor**	**0,39**	1,00E-09
**CD14 antigen**	**0,39**	1,00E-07
**MRNA; cDNA DKFZp564F053 (from clone DKFZp564F053)**	**0,39**	6,14E-04
**microtubule-associated protein, RP/EB family, member 3**	**0,38**	1,00E-09
**v-myb myeloblastosis viral oncogene homolog (avian) /// v-myb myeloblastos**	**0,37**	8,42E-04

**CDNA FLJ44318 fis, clone TRACH3000780**	**0,37**	3,15E-04
**fibroblast growth factor receptor 2 (bacteria-expressed kinase, keratinocyte g**	**0,37**	1,00E-09
**nebulette**	**0,36**	3,14E-04
**solute carrier family 39 (zinc transporter), member 8**	**0,36**	1,00E-09
**ELAV (embryonic lethal, abnormal vision, Drosophila)-like 3 (Hu antigen C) ///**	**0,35**	1,00E-09
**hypothetical protein PRO1843**	**0,34**	1,00E-09
**glycoprotein Ib (platelet), beta polypeptide**	**0,34**	1,00E-09
**ribosomal protein L14**	**0,33**	1,00E-09
**solute carrier family 39 (zinc transporter), member 6**	**0,32**	2,48E-05
**4-aminobutyrate aminotransferase**	**0,32**	1,04E-04
**serine (or cysteine) proteinase inhibitor, clade A (alpha-1 antiproteinase, antit**	**0,32**	3,84E-04
**UDP-glucose ceramide glucosyltransferase /// UDP-glucose ceramide glucosy**	**0,30**	1,08E-05
**GATA binding protein 3 /// GATA binding protein 3**	**0,30**	4,85E-04
**GATA binding protein 3**	**0,29**	4,54E-04
**KIAA0882 protein**	**0,29**	8,00E-07
**nuclear receptor interacting protein 1**	**0,28**	3,30E-06
**DnaJ (Hsp40) homolog, subfamily C, member 12 /// DnaJ (Hsp40) homolog, su**	**0,26**	2,28E-05
**ribosomal protein L38 /// ribosomal protein L38**	**0,26**	1,00E-09
**UDP-Gal:betaGlcNAc beta 1,4- galactosyltransferase, polypeptide 1 /// UDP-Ga**	**0,25**	1,00E-09
**solute carrier family 39 (zinc transporter), member 6**	**0,23**	2,28E-05
**MAX binding protein**	**0,23**	1,00E-09
**trefoil factor 3 (intestinal) /// trefoil factor 3 (intestinal)**	**0,22**	9,89E-04
**ribosomal protein S19**	**0,22**	1,00E-09
**solute carrier family 35, member F2**	**0,21**	1,00E-09
**ribosomal protein L27a**	**0,21**	1,00E-09
**ribosomal protein L27**	**0,20**	1,00E-09
**Similar to hypothetical protein FLJ25976 (LOC401082), mRNA**	**0,19**	1,00E-09
**insulin-like growth factor 1 receptor**	**0,16**	2,35E-05
**estrogen receptor 1 /// estrogen receptor 1**	**0,11**	5,50E-06
**ribosomal protein S11**	**0,07**	1,00E-09
