# Supplementary material for: Exquisite Sensitivity of TP53 Mutant and Basal Breast Cancers to a Dose-Dense Epirubicin−Cyclophosphamide Regimen
Source: PLoS Med. 2007 Mar 20;4(3):e90. doi: 10.1371/journal.pmed.0040090 (PMC1831731; doi:10.1371/journal.pmed.0040090)
Supplement: Table S8 — Note that several of the top genes (when ordered by decreasing fold-change values) were previously identified in the complete population (Table S7). Genes in common with Table S7 are highlighted. (31 KB PDF) [file pmed.0040090.st008.pdf]

Gene Symbol	Gene Title	FC mut vs wt (all samples)	p-value (all samples)	FC mut vs wt (ER+ only)	p-value (ER+ only)
KRT7	keratin 7	5,26	1,02E-05	5,14	0,0005314
PHLDA2	member 2	4,00	1,61E-05	4,96	9,63E-05
RIS1	Ras-induced senescence 1	4,00	1,39E-05	4,20	6,60E-06
CDC20	cerevisiae)	3,33	1,90E-06	3,65	6,50E-06
GPR172A	G protein-coupled receptor 172A	2,13	3,21E-04	3,36	7,89E-05
TGFB1I1	transcript 1	2,27	2,12E-04	3,33	9,31E-05
FLJ10901	hypothetical protein FLJ10901	3,57	9,58E-05	3,21	0,0030655
ADM	adrenomedullin	3,57	1,57E-05	3,14	0,0004888
FLJ12442	hypothetical protein FLJ12442	2,56	2,32E-05	3,07	6,80E-06
LOXL2	lysyl oxidase-like 2	2,13	9,17E-04	2,93	0,0001683
SLC6A8	solute carrier family 6 (neurotransmitter transporter, creatine), member 8	2,22	3,48E-04	2,85	6,37E-05
H2AFX	H2A histone family, member X	2,04	3,56E-05	2,74	0,0001165
BAX	BCL2-associated X protein	2,17	4,37E-05	2,65	0,0017637
MAFF	v-maf musculoaponeurotic fibrosarcoma oncogene homolog F (avian)	3,85	1,22E-05	2,54	0,0059437
SHD1	Sac3 homology domain 1 (S. cerevisiae)	2,08	5,30E-05	2,47	7,37E-05
RKHD1	ring finger and KH domain containing 1	2,50	1,40E-06	2,46	0,0017892
DPYSL3	dihydropyrimidinase-like 3	2,00	3,60E-04	2,38	0,0003074
PHLDA1	member 1	2,50	9,40E-04	2,36	8,79E-05
PTP4A3	3	1,92	1,89E-04	2,34	9,70E-06
ICAM1	intercellular adhesion molecule 1 (CD54), human rhinovirus receptor	2,38	4,49E-05	2,32	0,0020223
DBN1	drebrin 1	2,08	1,04E-04	2,29	9,55E-05
SPHK1	sphingosine kinase 1	1,89	2,43E-04	2,29	3,60E-06
APOBEC3B	apolipoprotein B mRNA editing enzyme, catalytic polypeptide-like 3B	3,03	1,71E-04	2,25	0,0053521
THBS1	thrombospondin 1	2,38	2,98E-04	2,25	0,0050724
PSMF1	proteasome (prosome, macropain) inhibitor subunit 1 (PI31)	1,54	9,41E-04	2,24	4,02E-05
SDC3	syndecan 3 (N-syndecan)	1,61	8,13E-04	2,22	0,000441
NXN	nucleoredoxin	2,50	3,60E-06	2,19	0,008867
KIAA1196	KIAA1196 protein	1,67	4,96E-04	2,19	0,0003049
GAS1	growth arrest-specific 1	2,08	7,37E-04	2,18	0,0072792
NEK2	NIMA (never in mitosis gene a)-related kinase 2	2,17	2,16E-04	2,17	0,0017947
ATP6V0D1	ATPase, H+ transporting, lysosomal 38kDa, V0 subunit d isoform 1	1,54	7,24E-04	2,16	0,0054999
MAP2K3	mitogen-activated protein kinase kinase 3 /// mitogen-activated protein kinase kinase 3	2,08	< 1.0E-07	2,15	6,50E-06
PHGDH	phosphoglycerate dehydrogenase	2,44	1,56E-05	2,15	0,0004072
PLAUR	plasminogen activator, urokinase receptor	1,89	5,50E-06	2,14	3,36E-05
HSPC163	HSPC163 protein	2,04	9,29E-05	2,14	0,0015983
SLC25A1	solute carrier family 25 (mitochondrial carrier; citrate transporter), member 1	1,67	2,25E-05	2,14	0,0002216
EHD1	EH-domain containing 1	1,69	3,53E-05	2,13	0,0017354
FLJ22222	hypothetical protein FLJ22222	2,22	2,53E-05	2,13	0,0006085
DDA3	differential display and activated by p53	1,89	5,25E-05	2,12	6,79E-05
AP2S1	subunit	1,47	9,70E-04	2,11	0,0041585
CD300A	CD300A antigen	1,67	1,50E-04	2,10	0,0007573
CSDA	cold shock domain protein A	2,17	1,03E-04	2,10	0,0061808
PACS1L	phosphofurin acidic cluster sorting protein 1-like	1,54	4,55E-04	2,08	5,20E-06
GTPBP1	GTP binding protein 1	1,69	4,20E-04	2,07	4,71E-05
CEBPB	CCAAT/enhancer binding protein (C/EBP), beta	1,72	5,04E-05	2,06	0,0038675
CDK9	kinase)	1,69	2,76E-04	2,06	0,0009006
DBN1	drebrin 1	1,89	2,31E-04	2,06	0,0004691
EFNA4	ephrin-A4	1,72	9,72E-04	2,05	0,0027032
SMTN	smoothelin	1,69	9,00E-07	2,04	4,00E-07
PLTP	phospholipid transfer protein	1,75	9,20E-04	2,03	0,0014745
SERPINE1	(nexin, plasminogen activator inhibitor type 1), member 1	1,64	1,59E-04	2,02	0,0001419
TUBA1	tubulin, alpha 1 (testis specific)	1,89	2,32E-04	2,02	0,0007936
