# Supplementary material for: Exquisite Sensitivity of TP53 Mutant and Basal Breast Cancers to a Dose-Dense Epirubicin−Cyclophosphamide Regimen
Source: PLoS Med. 2007 Mar 20;4(3):e90. doi: 10.1371/journal.pmed.0040090 (PMC1831731; doi:10.1371/journal.pmed.0040090)
Supplement: Table S9 — (42 KB PDF) [file pmed.0040090.st009.pdf]

Probe Set ID	Fold change (Cytokeratine 5/6 17 pos/neg)	p-value	Entrez Gene	Gene Symbol	Gene Title
209351_at	12,37	1,96E-04	3861	KRT14	keratin 14 (epidermolysis bullosa simp
213680_at	8,31	2,11E-04	3854	KRT6B	keratin 6B
205157_s_at	8,22	1,03E-05	3872	KRT17	keratin 17
202274_at	6,51	2,29E-04	72	ACTG2	actin, gamma 2, smooth muscle, enter
204855_at	6,38	9,10E-05	5268	SERPINB5	serine (or cysteine) proteinase inhibito
222242_s_at	5,47	8,06E-04	25818	KLK5	kallikrein 5
205475_at	4,71	1,73E-04	11341	SCRG1	scrapie responsive protein 1
209283_at	4,69	6,89E-04	1410	CRYAB	crystallin, alpha B
209842_at	4,54	1,57E-04	6663	SOX10	SRY (sex determining region Y)-box 1
201820_at	4,06	9,83E-04	3852	KRT5	keratin 5 (epidermolysis bullosa simple
215034_s_at	3,64	1,89E-05	4071	TM4SF1	transmembrane 4 superfamily membe
209387_s_at	3,60	7,71E-05	4071	TM4SF1	transmembrane 4 superfamily membe
213456_at	3,54	7,66E-05	25928	SOSTDC1	sclerostin domain containing 1
209386_at	3,11	2,20E-04	4071	TM4SF1	transmembrane 4 superfamily membe
204437_s_at	2,99	4,47E-04	2348	FOLR1	folate receptor 1 (adult)
214240_at	2,99	9,62E-04	51083	GAL	galanin
206032_at	2,86	3,04E-04	1825	DSC3	desmocollin 3
202267_at	2,64	1,78E-04	3918	LAMC2	laminin, gamma 2
206033_s_at	2,48	8,07E-04	1825	DSC3	desmocollin 3
210605_s_at	2,34	9,78E-04	4240	MFGE8	milk fat globule-EGF factor 8 protein
55081_at	2,29	2,88E-04	85377	MICAL-L1	MICAL-like 1
204401_at	2,18	6,22E-05	3783	KCNN4	potassium intermediate/small conduct
219615_s_at	2,05	2,37E-05	8645	KCNK5	potassium channel, subfamily K, mem
205595_at	1,98	9,60E-06	1830	DSG3	desmoglein 3 (pemphigus vulgaris ant
219225_at	1,97	9,11E-04	79605	PGBD5	piggyBac transposable element derive
219735_s_at	1,90	7,97E-04	29842	TFCP2L1	transcription factor CP2-like 1
221779_at	1,75	9,77E-04	85377	MICAL-L1	MICAL-like 1
201751_at	1,67	1,68E-04	9929	KIAA0063	KIAA0063 gene product
210098_s_at	1,46	9,05E-04	---	---	---
207183_at	1,45	5,18E-04	2842	GPR19	G protein-coupled receptor 19
205938_at	1,37	4,34E-04	22843	PPM1E	protein phosphatase 1E (PP2C domai
215195_at	1,35	8,31E-05	5578	PRKCA	protein kinase C, alpha
206421_s_at	1,29	8,17E-04	8710	SERPINB7	serine (or cysteine) proteinase inhibito
209559_at	0,73	5,83E-04	9026	HIP1R	huntingtin interacting protein-1-related
204621_s_at	0,52	2,11E-04	4929	NR4A2	nuclear receptor subfamily 4, group A,
213068_at	0,50	9,55E-04	1805	DPT	dermatopontin
218205_s_at	0,46	6,63E-05	2872	MKNK2	MAP kinase interacting serine/threonir
